# Perioperative tislelizumab plus chemotherapy for locally advanced gastroesophageal junction adenocarcinoma (NEOSUMMIT-03): a prospective, nonrandomized, open-label, phase 2 trial

**DOI:** 10.1038/s41392-025-02160-8

**Published:** 2025-02-05

**Authors:** Run-Cong Nie, Shu-Qiang Yuan, Ya Ding, Yong-Ming Chen, Yuan-Fang Li, Cheng-Cai Liang, Mu-Yan Cai, Guo-Ming Chen, Wei Wang, Xiao-Wei Sun, De-Sheng Weng, Dan-Dan Li, Jing-Jing Zhao, Xiao-Jiang Chen, Yuan-Xiang Guan, Zhi-Min Liu, Yao Liang, Ma Luo, Jun Chi, Hai-Bo Qiu, Zhi-Wei Zhou, Xiao-Shi Zhang, Ying-Bo Chen

**Affiliations:** 1 Department of Gastric Surgery, Sun Yat-sen University Cancer Center, State Key Laboratory of Oncology in South China, Collaborative Innovation Center for Cancer Medicine, Guangzhou, PR China; 2 Department of Biotherapy, Sun Yat-sen University Cancer Center, State Key Laboratory of Oncology in South China, Collaborative Innovation Center for Cancer Medicine, Guangzhou, PR China; 3 Department of Pathology, Sun Yat-sen University Cancer Center, State Key Laboratory of Oncology in South China, Collaborative Innovation Center for Cancer Medicine, Guangzhou, PR China; 4 Department of Medical Imaging, Sun Yat-sen University Cancer Center, State Key Laboratory of Oncology in South China, Collaborative Innovation Center for Cancer Medicine, Guangzhou, PR China; 5 Department of Endoscopy, Sun Yat-sen University Cancer Center, State Key Laboratory of Oncology in South China, Collaborative Innovation Center for Cancer Medicine, Guangzhou, PR China

**Keywords:** Gastrointestinal cancer, Drug development

## Abstract

This prospective, nonrandomized, open-label phase 2 trial (Chinese Clinical Trial Registry, ChiCTR2200061906) aimed to evaluate the effectiveness of adding the PD-1 antibody tislelizumab to perioperative chemotherapy in patients with locally advanced gastroesophageal junction adenocarcinoma (GEJA). This study enrolled patients with GEJA clinically staged as cT3-4aNanyM0 or cT1-2N+M0 from October 2022 to June 2023. Eligible patients were administered three preoperative and five postoperative 3-week cycles of treatment with PD-1 antibody tislelizumab plus SOX (S-1 and oxaliplatin) regimen. The primary endpoint was major pathological response (MPR) rate. Thirty-two patients were enrolled. The median age was 60 years (range: 28–74 years), and 53.1% (17/32) patients were Siewert III type. All patients received at least one cycle of assigned preoperative treatment, and 93.8% (30/32) patients completed three cycles of assigned preoperative tislelizumab and SOX. The R0 resection rate was 96.9% (31/32). MPR, pathological complete response (pCR) of primary tumors and ypT0N0 rates were 50.0% (16/32, 95% CI: 31.9–68.1%), 28.1% (9/32, 95% CI: 13.7–46.7%) and 25.0% (8/32, 95% CI: 11.5–43.4%), respectively. The surgical morbidity rate was 15.6% (5/32), and no 30-day mortality was observed. In the preoperative and postoperative treatment periods, the rate of treatment-related grade 3–4 adverse events was 31.2% (10/32). At the date of 7^th^ Jan 2025, 8 (25.0%) patients occurred recurrence. Therefore, perioperative tislelizumab plus chemotherapy demonstrated significantly improved pathological regression and might be a promising option for patients with locally advanced resectable GEJA.

## Introduction

Gastric and gastroesophageal junction adenocarcinoma ranks fifth among malignancies in terms of both incidence and mortality worldwide.^1^ While the overall prevalence of gastric adenocarcinoma has steadily decreased, an increase has been observed in the occurrence of gastroesophageal junction adenocarcinoma (GEJA) in the past 30 years.^2,3^ Moreover, about 61% of all GEJA case are diagnosed in China.^3^ GEJA cases are frequently diagnosed at a locally advanced stage,^4^ leading to a generally grim prognosis.^5,6^

Besides anatomical location, GEJA possesses unique molecular characteristics, such as a less common association with *H. pylori* infection, a more frequent association with chromosomal instability (CIN) subtype, and amplification of ERBB2 and EGFR.^7^ Patients diagnosed with GEJA should be categorized as having a distinct type of tumor and should receive specialized treatment. However, the scarcity of randomized controlled trials (RCTs) focusing on GEJA has resulted in a lack of globally accepted standard of care for these cases.^8–11^ Given that a substantial proportion of GEJA cases were included in RCTs assessing both esophageal carcinoma.^12,13^ and gastric carcinoma,^14,15^ the standard of care for patients with resectable GEJA corresponds to that of esophageal carcinoma or gastric carcinoma cases.^8,9^ Based on the successful outcomes of the FLOT4 study,^14^ perioperative fluorouracil, leucovorin, oxaliplatin and docetaxel (FLOT) is recommended for patients with locally advanced GEJA in non-Asian countries.^8,11^ In Japan and Korea,^16,17^ adjuvant chemotherapy following D2 gastrectomy is preferred in locally advanced GEJA due to the promising results of the CLASSIC and ACTS-GC studies.^18,19^ In 2021, the RESOLVE study reported a clinically meaningful improvement in disease-free survival after treatment with perioperative S-1 and oxaliplatin (SOX) versus adjuvant capecitabine and oxaliplatin (CapOx) in individuals with locally advanced gastric carcinoma or GEJA.^15^ These findings supported the adoption of perioperative SOX as the standard of care for localized GEJA cases in China.^10^ Although perioperative chemoradiotherapy is considered the standard care for patients with esophageal cancer,^12^ the recent TOPGEAR study indicated that incorporating radiotherapy into chemotherapy during the perioperative phase failed to improve survival outcomes for patients with locally advanced gastric cancer and GEJA.^20^ Therefore, there is an urgent need to develop effective treatment strategies for these patients.

Therapies including programmed cell death 1 (PD-1) or programmed death-ligand 1 (PD-L1) blockade, when combined with chemotherapy, improve patient survival in gastric carcinoma or GEJA and are thus endorsed for the first-line treatment of these cases.^21–27^ The RATIONALE-305 study demonstrated significantly improved overall survival (OS) after treatment with tislelizumab in combination with chemotherapy compared to placebo plus chemotherapy.^24^ In addition, tislelizumab also showed promising antitumor activity in the perioperative setting in individuals with localized tumors.^28^ Several RCTs have reported that addition of perioperative PD-1/PD-L1 inhibitors to chemotherapy substantially improves pathological response.^29–31^ However, little is known about the perioperative application of PD-1 inhibitors in individuals with locally advanced GEJA.

In this study, we report the results of the prospective, nonrandomized, open-label, phase 2 NEOSUMMIT-03 study that evaluated the efficacy of perioperative tislelizumab plus chemotherapy for locally advanced GEJA.

## Results

### Baseline patient characteristics

**Fig. 1:**
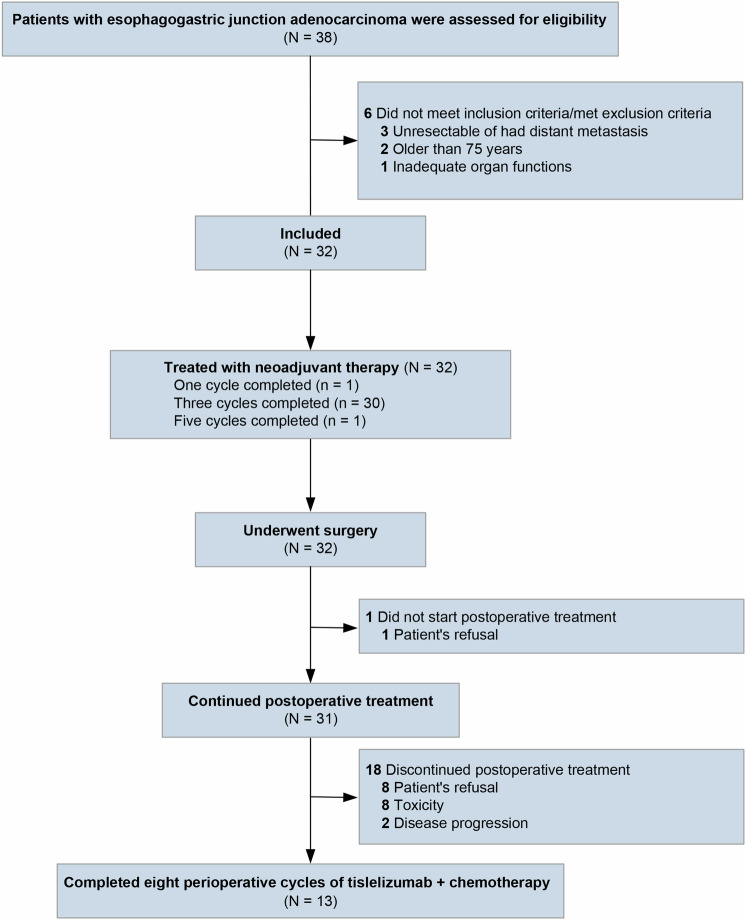
CONSORT diagram

Between October 10, 2022 and June 15, 2023, 38 patients with GEJA were screened for eligibility (Fig. 1). Of these patients, 6 not meeting the inclusion criteria were excluded; therefore, 32 patients were included. The baseline patient characteristics are shown in Table 1. The median age was 60 years (range: 28–74 years), and 78.1% (25/32) patients were male. Of the 32 cases included, 17 (53.1%) were Siewert type III, 12 (37.5%) had the intestinal type, and 18 (56.3%) had a PD-L1 combined positive score (CPS) of 5 or more. All the 32 cases were mismatch repair-proficient (pMMR), and 2 (6.2%) were confirmed to be Epstein-Barr virus (EBV) positive. Diagnostic laparoscopic exploration was carried out in 22 (68.8%) of the 32 patients.

**Table 1:** Baseline characteristics of the included patients (*N* = 32)

Characteristic	Patients, No. (%)
Age, years	
Median (IQR)	60 (52–66)
<65	21 (65.6%)
Sex	
Male	25 (78.1%)
Female	7 (21.9%)
ECOG performance status	
0	32 (100%)
Siewert classification	
Type II	15 (46.9%)
Type III	17 (53.1%)
Clinical T stage	
cT3	20 (62.5%)
cT4a	12 (37.5%)
Clinical N stage	
cN0–1	11 (34.4%)
cN2–3	21 (65.6%)
Lauren’s classification	
Diffuse	9 (28.1%)
Intestinal	12 (37.5%)
Mixed	11 (34.4%)
PD-L1 CPS	
<1	2 (6.2%)
≥1	30 (93.8%)
<5	14 (43.8%)
≥5	18 (56.3%)
<10	22 (68.8%)
≥10	10 (31.2%)
MMR status	
pMMR	32 (100%)
EBV status	
Positive	2 (6.3%)
Negative	30 (93.8%)
Laparoscopic exploration	
Yes	22 (68.8%)
No	10 (31.2%)

Data are *n* (%) or median (IQR), unless otherwise indicated. Percentages may not add up to 100 because of rounding. *ECOG* Eastern Cooperative Oncology Group, *PD-L1* programmed cell death-ligand 1, *CPS* combined positive score, *MMR* mismatch repair, *pMMR* mismatch repair-proficient, *EBV* Epstein-Barr virus

### Neoadjuvant tislelizumab and chemotherapy

All the included 32 patients received at least one cycle of the assigned neoadjuvant tislelizumab and chemotherapy. Of these patients, 30 (93.8%) completed the planned 3 cycles of neoadjuvant therapy. One patient (3.1%) only completed one cycle of neoadjuvant tislelizumab and chemotherapy because of grade 3 diarrhea and per the patient’s request. One patient (3.1%) had grade 3 hypothyroidism after the third cycle of neoadjuvant tislelizumab and chemotherapy. Due to the high anesthetic risk at that time, the patient was deemed unsuitable for surgery, was subsequently administered two additional cycles of tislelizumab and chemotherapy, and started thyroid hormone replacement therapy. RECIST 1.1 evaluation was performed in 32 cases after completion of neoadjuvant treatment prior surgery: complete response, *n* = 1 (3.1%); partial response, *n* = 10 (31.3%); stable disease, *n* = 1 (3.1%); not evaluable, *n* = 20 (62.5%).

### Surgical findings

After neoadjuvant treatment, all the 32 patients underwent surgical resection. The median time span from the end of the final round of neoadjuvant therapy to surgical intervention was 2.8 weeks. (range: 2.1–12.1 weeks). One patient had delayed surgery per the patient’s request. The surgery type and overall morbidity are shown in Supplementary Table 1. Total gastrectomy was carried out in 27 (84.4%) cases, whereas R0 resection was performed in 31 (96.9%) individuals. Five (15.6%) cases reported at least one serious adverse event associated with perioperative morbidity. There was no mortality reported within 30-day.

**Fig. 2:**
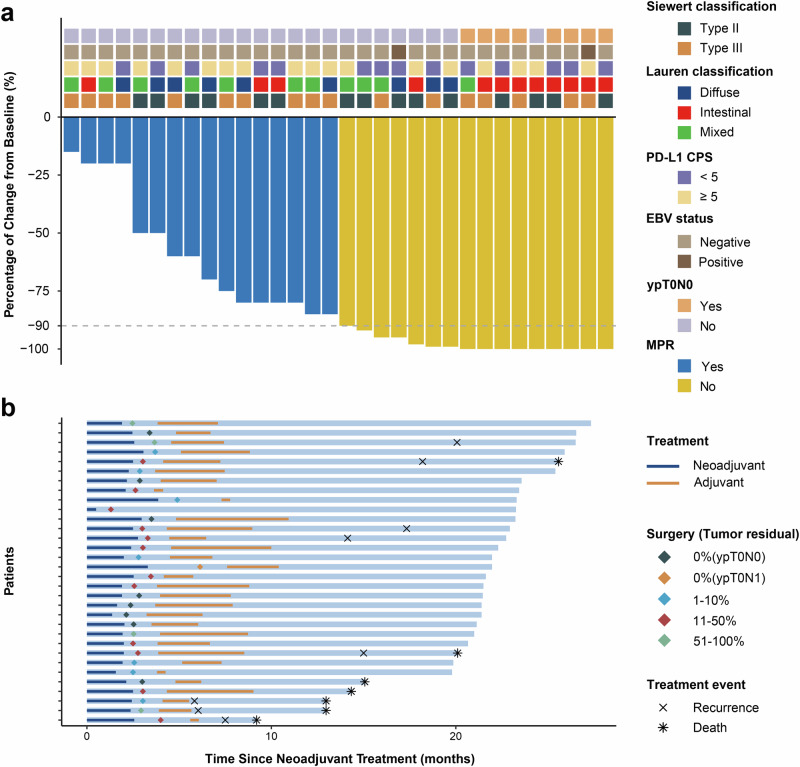
Tumor response to neoadjuvant therapy and follow-up. **a** Waterfall plot of tumor regression rates after neoadjuvant therapy based on pathology. **b** Swimmer plot showing treatment events and follow-up in the population of all included patients. PD-L1, programmed cell death-ligand 1, CPS, combined positive score; EBV, Epstein-Barr virus; MPR, major pathological response

Pathological and tumor regression data are shown in Table 2 and Fig. 2a. The median tumor residual rate was 12.5% (range: 0–85%, Fig. 2a). The primary endpoint was major pathological response (MPR) of the included patients. Of the 32 patients, 16 (50.0%, 95% CI: 31.9–68.1) had MPR. The TRG results are summarized in Table 2. Nine (28.1%, 95% CI: 13.7–46.7) cases achieved pathological complete response (pCR) in the primary tumor, one of whom was T0 but with tumor cells in two nodes and was staged as ypT0N1. Therefore, eight (25.0% (8/32, 95% CI: 11.5–43.4%) cases achieved ypT0N0. Fourteen (43.8%, 95% CI: 26.4–62.3) cases achieved TRG 0/1.

**Table 2:** Pathological and tumor regression (*N* = 32)

Characteristic	Patients, No. (%)
Pathological T stage (ypT)	
ypT0	9 (28.1%)
ypT1	3 (9.4%)
ypT2	6 (18.8%)
ypT3	11 (34.4%)
ypT4	3 (9.4%)
Combined ypT0–2	18 (56.3%)
Combined ypT3–4	14 (43.8%)
Pathological N stage	
ypN0	23 (71.9%)
ypN1	6 (18.8%)
ypN2	2 (6.2%)
ypN3	1 (3.1%)
Combined ypN1–3	9 (28.1%)
TRG	
TRG 0	9 (28.1%)
TRG 1	5 (15.6%)
TRG 2	14 (43.8%)
TRG 3	4 (12.5%)
Combined TRG 0–1	14 (43.8%)
Tumor residual percentage	
0%	9 (28.1%)
1–10%	7 (21.9%)
11–50%	12 (37.5)
51–100%	4 (12.5%)
MPR (0–10%)	16 (50.0%)

Data are n (%). Percentages may not add up to 100 because of rounding. TRG, tumor regression grade; MPR, major pathological response

### Adjuvant tislelizumab and chemotherapy

The adjuvant treatment is described in Fig. 1 and Fig. 2b. Of the 32 patients administered surgery, 31 (96.9%) cases were administered adjuvant tislelizumab and chemotherapy. One patient did not receive adjuvant treatment per the patient’s request. The median time span from surgery to the initiation of adjuvant therapy was 5.6 weeks (range: 3.0–11.1 weeks). Thirteen (40.6%) cases completed the planned 8 perioperative treatments with tislelizumab and chemotherapy.

### Survival

**Fig. 3:**
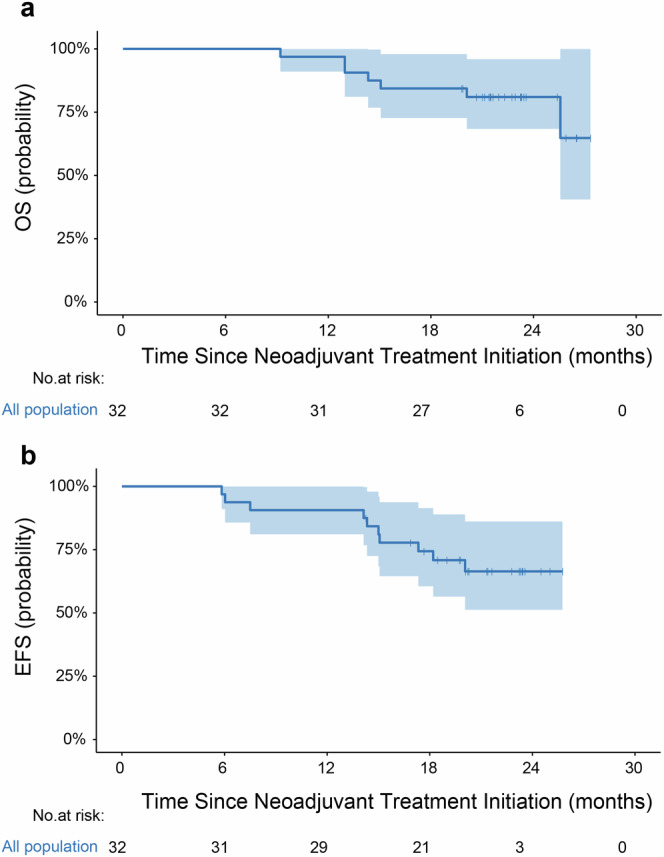
Kaplan-Meier plots for (**a**) OS and (**b**) EFS. OS, overall survival; EFS, event-free survival

At the data cutoff on January 7, 2025, the median follow-up time was 21.9 months (range: 11.2–27.3). Of the 32 cases, 22 (68.8%) were alive and free of recurrence. Six cases had peritoneal metastasis, two suffered from distant lymph node metastasis. Among 8 patients with recurrent disease, 6 patients were alive, 2 patients died of progressive disease. Two patient without recurrent disease died of non-tumor-related causes. Kaplan-Meier curves for overall survival (OS) and event-free survival (EFS) are shown in Fig. 3, respectively. The 18-month OS and EFS rates were 84.4% (95% CI: 72.7–97.9%) and 74.4% (95% CI: 60.5–91.4%), respectively.

### Adverse events

Treatment-related adverse events (TRAEs) of any cause occurred in all patients during perioperative therapy (Supplementary Table 2). Grade 3 or 4 TRAEs were observed in 31.2% (10/32) of patients. Of these, thrombocytopenia (*n* = 3, 9.4%) was the most common grade 3 or 4 TRAE. Immune-relative AEs (irAEs) occurred in 8 (25.0%) patients, with most being grade 1 or 2. One patient experienced grade 3 hypothyroidism. There was no death considered to be treatment related.

### Biomarker analysis

**Fig. 4:**
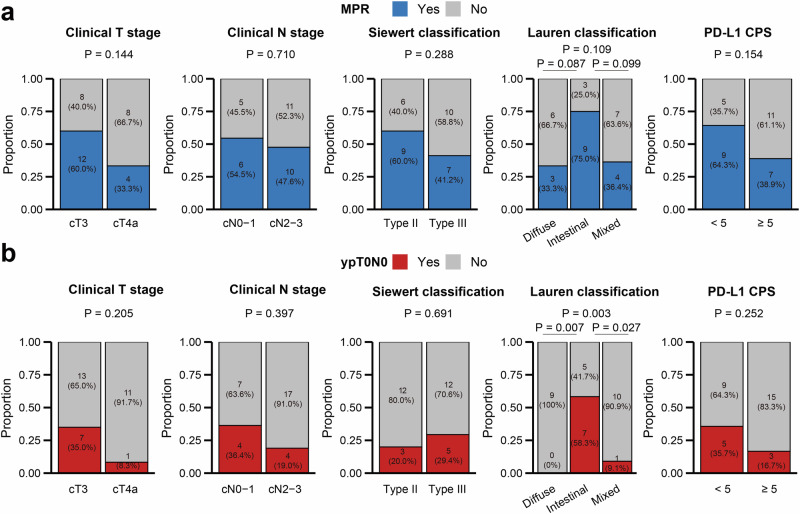
Histopathological tumor regression rates by clinicopathological characteristics. **a** MPR; **b** ypT0N0. MPR major pathological response, PD-L1 programmed cell death-ligand 1, CPS combined positive score

In post hoc analysis, subgroup analyses were carried out to assess tumor regression by clinical T stage, clinical N stage, Siewert type, Lauren classification, and PD-L1 CPS status (Fig. 4). Of note, a larger percentage of patients with intestinal type had MPR (9/12, 75.0% [95% CI: 42.8–94.5]) compared with cases with diffuse (3/9, 33.3% [95% CI: 7.5–70.1]; *P* = 0.087) or mixed (4/11, 36.4% [95% CI: 10.9–69.2]; *P* = 0.099) type. A higher ypT0N0 rate was observed in patients with intestinal type (7/12, 66.7% [95% CI: 30.4–86.2]) compared with cases with diffuse (0/9, 0% [95% CI: 0–33.6]; *P* = 0.007) or mixed (1/11, 9.1% [95% CI: 0.2–41.3]; *P* = 0.027) type. There were no significant differences in MPR or ypT0N0 rates between patients with a high CPS and those with a low CPS (Fig. 4b and Supplementary Fig. 1). We also performed subgroup analyses to assess the survival by PD-L1 CPS status (Supplementary Fig. 2). Interestingly, patients with a CPS of 5 or more appear to have shorter EFS and OS than those with a CPS of less than 5.

**Fig. 5:**
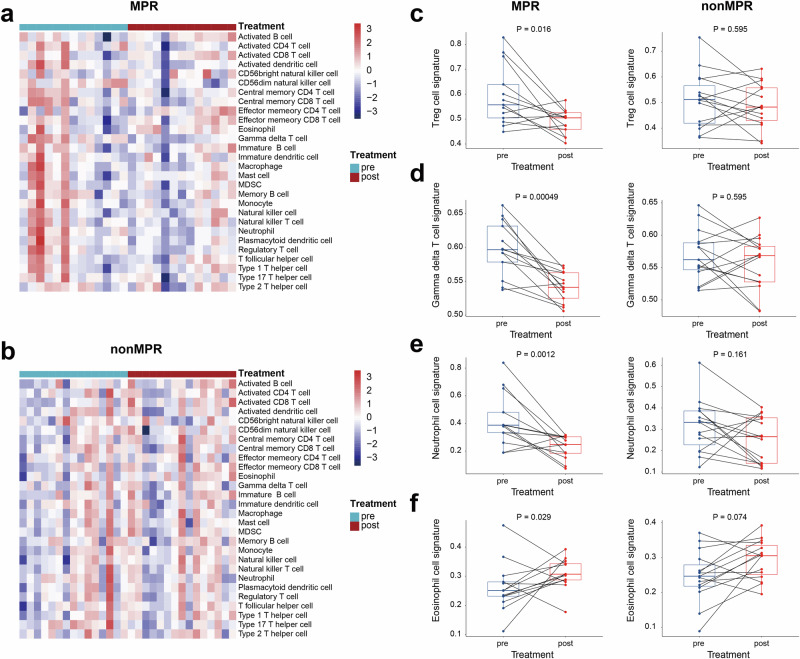
Immune cell landscape of patients at baseline and after neoadjuvant treatment. **a**, **b** Heatmaps depicting 28 subpopulations of tumor-infiltrating lymphocytes at baseline and after neoadjuvant treatment in patients who achieved a MPR (**a**, top panel) and those who did not (**b**, bottom panel); (**c**–**f**) Paired analysis of Treg (**c**), γδT (**d**), neutrophil (**e**) and eosinophils (**f**) cell infiltration at baseline and after neoadjuvant treatment in patients who achieved a MPR (left panel) and those who did not (right panel). MPR major pathological response. Wilcoxon signed-rank test

Subsequently, we conducted RNA sequencing on both paired baseline tumor biopsies and post-treatment samples from 28 patients to evaluate whether distinct immune cell components within the tumor immune microenvironment (TIME) control the treatment response (Fig. 5a, b). Of note, the baseline immune components could not predict the treatment response of neoadjuvant tislelizumab and chemotherapy. However, after neoadjuvant treatment, decreased levels of Treg, γδT and neutrophil cell infiltration were found in patients achieving MPR than in patients not achieving MPR (Fig. 5c–e). Besides, enhanced eosinophils infiltration was found in patients achieving MPR than in patients not achieving MPR (Fig. 5f).

## Discussion

This NEOSUMMIT-03 phase 2 study evaluated the application of a PD-1 inhibitor perioperatively for locally advanced GEJA and reached its primary objective for MPR (50% *vs* the planned primary endpoint of 33%). In addition, perioperative tislelizumab plus chemotherapy resulted in a promising pCR in primary tumors of 28.1% and a rate of 25.0% for ypT0N0, without increasing TRAEs and perioperative morbidity.

In patients diagnosed with Siewert Type I GEJA, the staging and treatment strategy is aligned with that of esophageal carcinoma. For Siewert Type III cases, the staging and treatment approach refers to gastric carcinoma, while in Siewert Type II cases, the staging and treatment routine remains unspecified. In this study, we included patients with Siewert type II and III GEJA and adopted SOX as the perioperative chemotherapy regimen because of the high proportion of GEJA (36.5%) cases in the RESOLVE study.^15^

The effect of neoadjuvant immunotherapy on gastric cancer has been examined in several studies. The randomized phase 2 DANTE study of perioperative atezolizumab plus FLOT initially reported an improved pathological response (Becker TRG 1a/b: 49% *vs* 44%).^27^ At the 2023 American Society of Clinical Oncology (ASCO) annual meeting, we reported the results of the randomized phase 2 NEOSUMMIT-01 study, which revealed improved major (44.4% *vs* 20.4%) or complete pathological (22.2% *vs* 7.4%) response.^29^ Subsequently at the European Society for Medical Oncology (ESMO) 2023 congress, improved pathological response was also reported for the phase 3 KEYNOTE-585.^30^ (pCR: 12.9% *vs* 2.0%) and MATTERHORN (pCR: 19% *vs* 7%) studies.^31^ In our NEOSUMMIT-03 study, we specifically focused on GEJA, and all eligible cases were pMMR. Patients with locally advanced GEJA administered neoadjuvant tislelizumab plus chemotherapy had promising MPR (50.0%), pCR (28.1%) and ypT0N0 (25.0%), surpassing the pathological response rate previously observed in the NEOSUMMIT-01 study examining similar cases administered neoadjuvant chemotherapy.^29^ Intriguingly, corroborating previous studies,^29,32^ one case had pCR of the primary tumor with tumor cells in lymph nodes in this study, indicating heterogeneous immune responses between the primary tumor and metastatic lymph node metastasis.

In previous studies of first-line PD-1/PD-L1 inhibitors plus chemotherapy for advanced gastric carcinoma, enhanced survival benefits corresponding to the PD-L1 expression status were detected based on the CPS.^21–25,27^ However, in post hoc analysis of the NEOSUMMIT-03 study, no significant difference was found in pathological response rate between patients with a CPS ≥ 5 and cases with a CPS below 5, which was also reported by NEOSUMMIT-01.^29^ and KEYNOTE-585.^30^ In our study, the identification of PD-L1 status was reliant on pretreatment biopsies, and the potential reason for CPS’s inability to effectively predict patient outcomes may lie in the spatial heterogeneity of PD-L1 expression.^33,34^ Of note, a post hoc analysis revealed a more pronounced pathological response in cases with the intestinal type compared with those with the diffuse or mixed type. This suggests that Lauren histological classification could be more important in exhibiting the benefits of perioperative anti-PD-1 therapy compared with PD-L1 status based on CPS. However, this result should be interpreted with caution given the limited sample size and the phase 2 nature of this study.

Currently, there are limited reports concerning the alterations in the TIME following neoadjuvant treatment with PD-1 inhibitors and chemotherapy for locally advanced resectable gastric cancer. In this study, we did not observe apparent baseline differences in TIME composition between patients with MPR and those without, consistent with findings reported in the advanced gastric cancer.^35^ Eosinophils are granulocytes derived from the bone marrow that play crucial roles in maintaining tissue homeostasis and repair, eliminating parasites, and contributing to the pathophysiology of various conditions, such as allergic asthma and autoimmune disorders.^36^ A recent study reported that an increase in intratumoral eosinophils was observed in breast cancer patients responding to anti-PD-1 therapy.^37^ Similarly, we observed that following neoadjuvant treatment with tislelizumab in combination with chemotherapy, an intensified infiltration of eosinophils was noted in patients who achieved a MPR. Therefore, eosinophils, an innate immune cell type, deserve greater more focus regarding their role in anti-tumor immunity.

Perioperative safety is of great importance for operable tumors. In this NEOSUMMIT-03 study, the frequency of grade 3 or 4 TRAEs was 31.2%, corroborating previous findings.^27,29,30^ No new safety signal was detected in this study, with acceptable perioperative morbidity (15.6%) and no 30-day mortality. However, despite the high completion rate of preoperative tislelizumab plus chemotherapy (93.8%), only 40.6% of all included patients completed the 8 scheduled perioperative treatments with tislelizumab and chemotherapy, which was lower than reported for NEOSUMMIT-01.^29^ This finding also called for the possibility of long-course or total neoadjuvant anti-PD1 therapy plus chemotherapy for such patients (Chinese Clinical Trial Registry, identifier: ChiCTR2300069000).

The limitations of this study should be acknowledged. Given that this was a single-arm phase 2 trial with a small sample size, these results should be interpreted with caution. Therefore, a large-scale RCT has been conducted to validate the effectiveness of perioperative tislelizumab combined with chemotherapy in patients with GEJA (Chinese Clinical Trial Registry, identifier: ChiCTR2400081098). In addition, although pathological response (MPR or pCR) has been demonstrated to predict survival outcomes in operable patients administered perioperative anti-PD-1/PD-L1 therapy,^38^ pCR enhancement did not translate into a survival benefit based on EFS in KEYNOTE-585.^30^ Therefore, it is necessary to continue follow-up until maturation to evaluate EFS, recurrence-free survival (RFS) and OS in this study.

In conclusion, the present study demonstrated that perioperative tislelizumab plus chemotherapy significantly improves pathological regression and might be a promising option for patients with locally advanced resectable GEJA.

## Materials and methods

### Study population and trial design

NEOSUMMIT-03 is a single-arm, open-label, phase 2 trial performed in China. Patients were eligible if they had histological confirmed GEJA of Siewert type II or III and clinical stage cT3-4aNanyM0 or cT1-2N+M0 based on Siewert classification.^39^ and the 8th Edition of the International Union against Cancer tumor-node-metastasis classification. The clinical stage was assessed via physical examination, oesophagogastroduodenoscopy, and CT or MRI scan of the thoracic, abdominal, and pelvic regions. Diagnostic laparoscopy was recommended to exclude occult peritoneal seeding. Patients were also required to be aged between 18 and 75 years, with an Eastern Cooperative Oncology Group (ECOG) performance status of 0–1, life expectancy ≥ 3 months, no contraindications for surgery, and adequate organ function. Patients with a history of anti-tumor drug therapy, or surgical treatment for gastric cancer, those with a history of other malignancies within the past 5 years, or those allergic to the investigational drug were excluded. Eligibility criteria are available in the study protocol (Additional information).

The protocol and its amendments were approved by the institutional review board or independent ethics committee of Sun Yat-sen University Cancer Center, and the study was performed in accordance with Good Clinical Practice guidelines and the Declaration of Helsinki. The results were reported in compliance with the CONSORT statement guidelines. All patients provided written informed consent.

### Treatment

In the neoadjuvant phase, patients were administered intravenously tislelizumab at 200 mg on day 1 of each cycle every 3 weeks for three cycles. Oxaliplatin at 130 mg/m^2^ was administered intravenously on day 1 of each cycle plus S-1 at 40–60 mg orally twice daily for 2 weeks followed by a one-week rest. Surgery was performed within 2–4 weeks after the last cycle of the neoadjuvant phase. In the adjuvant phase, between 4 and 6 weeks after radical gastrectomy, patients were scheduled for tislelizumab treatment at 200 mg plus chemotherapy for five cycles. Dose reduction for tislelizumab was not permitted and the drug could only be interrupted or discontinued for adverse events. Patients could have a maximum of two chemotherapy dose reductions; the detailed modifications of chemotherapy are provided in the protocol (Additional information).

### Assessments and outcomes

Imaging assessments by CT or MRI were carried out within 4 weeks before treatment initiation, after completion of cycle three of neoadjuvant treatment. Patients who had surgery and received adjuvant treatment were scheduled for repeat imaging every three months during the first three years, every six months until five years, and annually thereafter. The surgical procedures were performed by proficient abdominal surgeons with at least 100 gastric surgeries annually. In type II and III gastroesophageal junction adenocarcinoma cases, total or proximal gastrectomy with transhiatal distal esophagectomy was required. In this study, D2 lymphadenectomy was applied for total gastrectomy, and D1+lymphadenectomy for proximal gastrectomy. The quality of all surgeries was reviewed and evaluated by an experienced surgeon (YBC).

At baseline, Lauren histological classification, MMR status, PD-L1 status and EBV status were examined. The MMR status was determined by immunohistochemical (IHC) assessment of MLH1, PMS2, MSH2 and MSH6. PD-L1 expression was assessed using the 22C3 pharmDx assay (Agilent Technologies, Santa Clara, CA, USA) based on CPS., defined as the total number of PD-L1-positive tumor cells (partial or complete membrane staining), lymphocytes, and macrophages (membrane staining and/or intracellular staining) divided by the total number of viable tumor cells multiplied by 100. The EBV status was evaluated by EBER with an in-situ hybridization (ISH) kit (ISH-7001, Zhongshan Jinqiao Biotechnology Co., Ltd., Beijing, China/Leica Biosystems, Newcastle, UK).

The primary endpoint was MPR, defined as ≤ 10% residual viable tumor cells at the time of definitive surgery.^40^ Secondary endpoints included pCR of the primary tumor (ypT0), pathological regression response (TRG 0) or 1 as per the NCCN Guidelines for Gastric Cancer,^8^ margin-free resection (R0), RFS (duration from the initial treatment cycle to disease recurrence, metastasis, or death from any cause), EFS (time from the initial treatment cycle to disease progression, local or distant recurrence, or death from any cause), OS (time from initial treatment cycle to death from any cause), objective response rate (percentage of patients with partial or complete response as determined by investigators using RECIST version 1.1), surgical safety, treatment-related adverse events evaluated according to the CTCAE version 5.0, and quality of life.The pathological response was reviewed by a senior pathologist (MYC), whereas the radiological evaluation was carried out by a seasoned radiologist (ML).

### Biomarker analysis

We conducted RNA sequencing on both paired baseline tumor biopsies and post-treatment samples from 28 patients to evaluate whether distinct immune cell components within the TIME control the treatment response. Using the Gene Set Enrichment Analysis (GSEA) strategy.^41,42^ alongside previously defined immune cell markers,^43^ we estimated a total of 28 subpopulations of tumor-infiltrating lymphocytes, including major types associated with adaptive immunity.

### Statistical methods

The sample size was determined according to the assumption that addition of tislelizumab to chemotherapy would improve the MPR rate from the historical control rate of 13%.^44^ to 33%, with a one-sided α of 0.025 and a 80% power, accounting for a dropout rate of 10%. Therefore, it was expected that 32 patients would be included in the present study.

The population of all included patients was used to analyze the primary endpoint, surgical and pathological outcomes, non-surgical adverse events, serious adverse events, and survival outcomes. The primary efficacy endpoint (MPR rate) was reported as a proportion with 95% confidence interval (CI), determined by the Clopper-Pearson method. Other categorical variables were presented as frequency (percentage); continuous variables were reported as median (range). Survival data were estimated by the Kaplan-Meier method. Post hoc analyses were performed to evaluate tumor regression based on clinical T stage, clinical N stage, Siewert type, Lauren classification, and PD-L1 CPS status. We also performed subgroup analyses to assess the survival by PD-L1 CPS status. All P values were two-sided. SAS (version 9.4) was used for data analysis.

## Supplementary information

Supplementary Table 1 and 2, Figure 1 and 2

Protocol

## Data Availability

The raw sequencing data reported in this paper have been deposited in the Genome Sequence Archive in National Genomics Data Center, China National Center for Bioinformation/Beijing Institute of Genomics, Chinese Academy of Sciences (GSAHuman: HRA008806) that are publicly accessible at https://ngdc.cncb.ac.cn/gsa-human. All requests for data will be reviewed by the leading clinical site, Sun Yat-Sen University Cancer Center, to verify whether the request is subject to any intellectual property or confidentiality obligations. Requests for access to the patient-level data from this study can be submitted via email to nierc@sysucc.org.cn with detailed proposals for approval. A signed data access agreement with the sponsor is required before accessing shared data. Source data are provided with this paper.
