# Supplementary material for: Perioperative tislelizumab plus chemotherapy for locally advanced gastroesophageal junction adenocarcinoma (NEOSUMMIT-03): a prospective, nonrandomized, open-label, phase 2 trial
Source: Signal Transduct Target Ther. 2025 Feb 5;10:60. doi: 10.1038/s41392-025-02160-8 (PMC11799164; doi:10.1038/s41392-025-02160-8)
Supplement: Supplementary file 1 — Supplementary Table 1 and 2, Figure 1 and 2 [file 41392_2025_2160_MOESM1_ESM.docx]

Supplementary Materials for

Perioperative tislelizumab plus chemotherapy for locally advanced gastroesophageal junction adenocarcinoma (NEOSUMMIT-03): a prospective, nonrandomized, open-label, phase 2 trial

Authors: Run-Cong Nie^1†*^, Shu-Qiang Yuan^1†^, Ya Ding^2†^, Yong-Ming Chen^1†^, Yuan-Fang Li^1†^, Cheng-Cai Liang^1^, Mu-Yan Cai^3^, Guo-Ming Chen^1^, Wei Wang^1^, Xiao-Wei Sun^1^, De-Sheng Weng^2^, Dan-Dan Li^2^, Jing-Jing Zhao^2^, Xiao-Jiang Chen^1^, Yuan-Xiang Guan^1^, Zhi-Min Liu^1^, Yao Liang^1^, Ma Luo^4^, Jun Chi^5^, Hai-Bo Qiu^1^, Zhi-Wei Zhou^1^, Xiao-Shi Zhang^2*^, Ying-Bo Chen^1*^

Correspondence to: [chenyb@sysucc.org.cn;](mailto:chenyb@sysucc.org.cn;) [zhangxsh@sysucc.org.cn](mailto:zhangxsh@sysucc.org.cn); [nierc@sysucc.org.cn](mailto:nierc@sysucc.org.cn)

This PDF file includes:

Supplementary Table 1 and 2

Supplementary Fig. 1 and 2

**Supplemental Tables**

**Supplementary Table 1. Surgical outcomes and overall morbidity (N = 32)**

| Type of surgery | Patients, No. (%) |
| --- | --- |
| Type of surgery |  |
| Total gastrectomy | 27 (84.4%) |
| Proximal gastrectomy | 5 (15.6%) |
| Lymphadenectomy |  |
| D2 | 27 (84.4%) |
| D1+ | 5 (15.6%) |
| No. of nodes examined |  |
| Median (IQR) | 32 (28-42) |
| Resection grade |  |
| R0 | 31 (96.9%) |
| R1-2 | 1 (3.1%) |
| Clavien-Dindo grade, max/patients |  |
| No complication | 27 (84.4%) |
| I-II | 5 (15.6%) |
| III-IV | 0 |
| Overall postoperative complications ^a^ |  |
| Abdominal bleeding | 1 (3.1%) |
| Abdominal infection | 1 (3.1%) |
| Pancreatic fistula | 2 (6.3%) |
| Hydropneumothorax | 1 (3.1%) |
| Pneumonia | 3 (9.4%) |

Data are n (%) or median (IQR), unless otherwise indicated. IQR, interquartile range

a, For some patients more than one.

**Supplementary Table 2. Summary of adverse events (N = 32)**

| TRAE | No. (%) | | | | |
| --- | --- | --- | --- | --- | --- |
|  | Any grade | Grade 1 | Grade 2 | Grade 3 | Grade 4 |
| Any events | 32 (100.0%) | 4 (12.5%) | 18 (56.2%) | 9 (28.1%) | 1 (3.1%) |
| Thrombocytopenia | 11 (34.4%) | 0 | 8 (25.0%) | 2 (6.2%) | 1 (3.1%) |
| Leukopenia | 14 (43.8%) | 5 (15.6%) | 8 (25.0%) | 1 (3.1%) | 0 |
| Neutropenia | 13 (40.6%) | 3 (9.4%) | 9 (28.1%) | 1 (3.1%) | 0 |
| Anemia | 4 (12.5%) | 1 (3.1%) | 3 (9.4%) | 0 | 0 |
| ALT or AST or bilirubin increased | 10 (31.2%) | 4 (12.5%) | 6 (18.8%) | 0 | 0 |
| Vomit | 17 (53.1%) | 5 (15.6%) | 10 (31.2%) | 2 (6.2%) | 0 |
| Nausea | 17 (53.1%) | 5 (15.6%) | 10 (31.2%) | 2 (6.2%) | 0 |
| Diarrhea | 13 (40.6%) | 6 (18.8%) | 5 (15.6%) | 2 (6.2%) | 0 |
| Dysphagia | 2 (6.2%) | 1 (3.1%) | 1 (3.1%) | 0 | 0 |
| Decreased appetite | 7 (21.9%) | 6 (18.8%) | 1 (3.1%) | 0 | 0 |
| Constipation | 2 (6.2%) | 1 (3.1%) | 1 (3.1%) | 0 | 0 |
| Abdominal pain |  |  |  |  |  |
| Peripheral neuropathy | 4 (12.5%) | 1 (3.1%) | 2 (6.2%) | 1 (3.1%) | 0 |
| Immune-related AE | 8 (25.0%) | 5 (15.6%) | 2 (6.2%) | 1 (3.1%) | 0 |
| Rash | 5 (15.6%) | 5 (15.6%) | 0 | 0 | 0 |
| Hypothyroidism | 3 (9.4%) | 2 (6.2%) | 0 | 1 (3.1%) | 0 |
| Pruritus | 3 (9.4%) | 3 (9.4%) | 0 | 0 | 0 |
| Hypophysitis | 1 (3.1%) | 0 | 1 (3.1%) | 0 | 0 |

Data are n (%). Percentages may not add up to 100 because of rounding. TRAE, treatment related adverse event; AE, adverse event; ALT, Alanine aminotransferase; AST, Aspartate aminotransferase.

**Supplemental Figure**


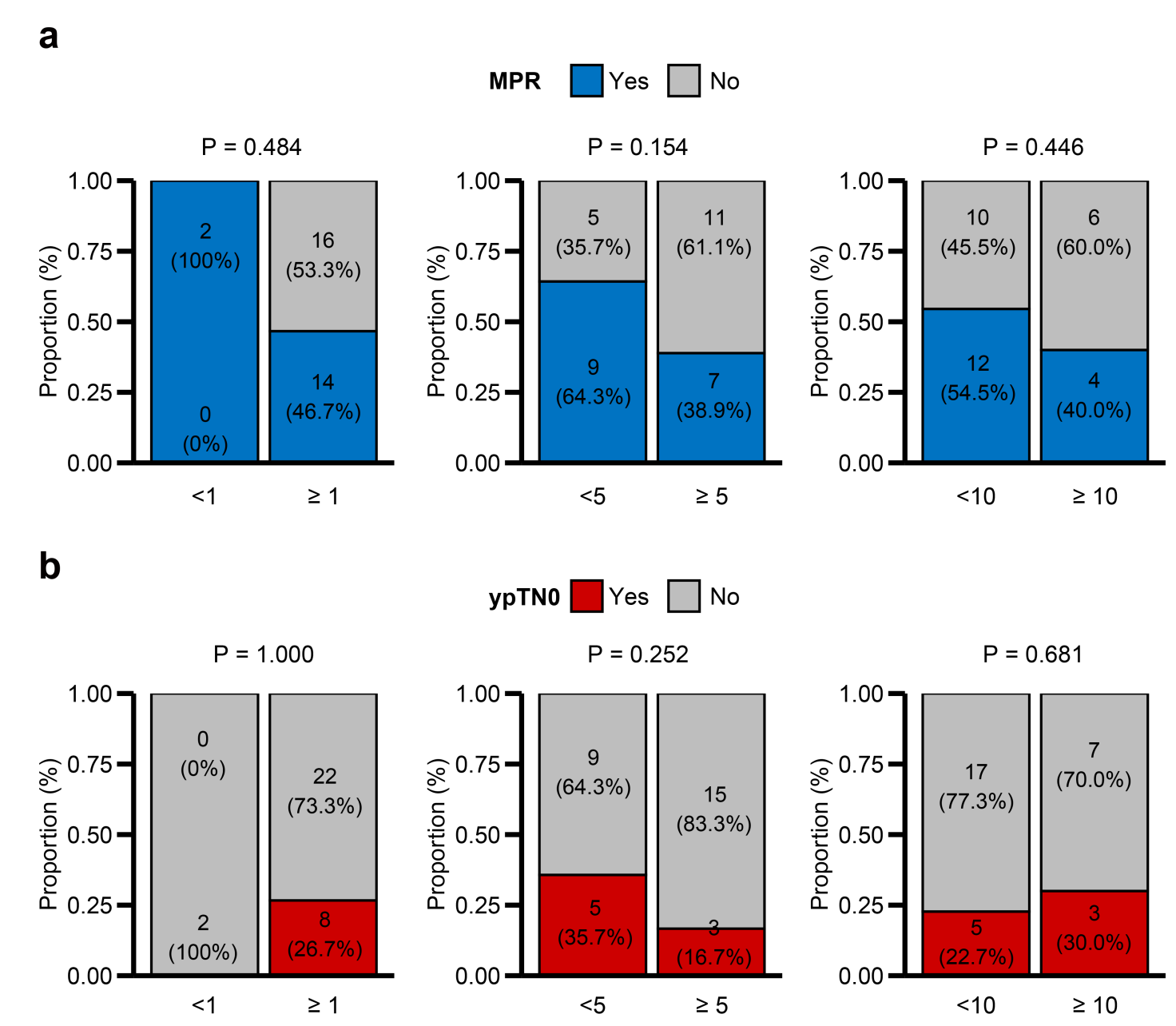


**Supplementary Fig. 1** **Histopathological tumor regression rates by PD-L1 CPS.** (a) MPR; (b) ypT0N0. MPR, major pathological response; PD-L1, programmed cell death-ligand 1, CPS, combined positive score.

**
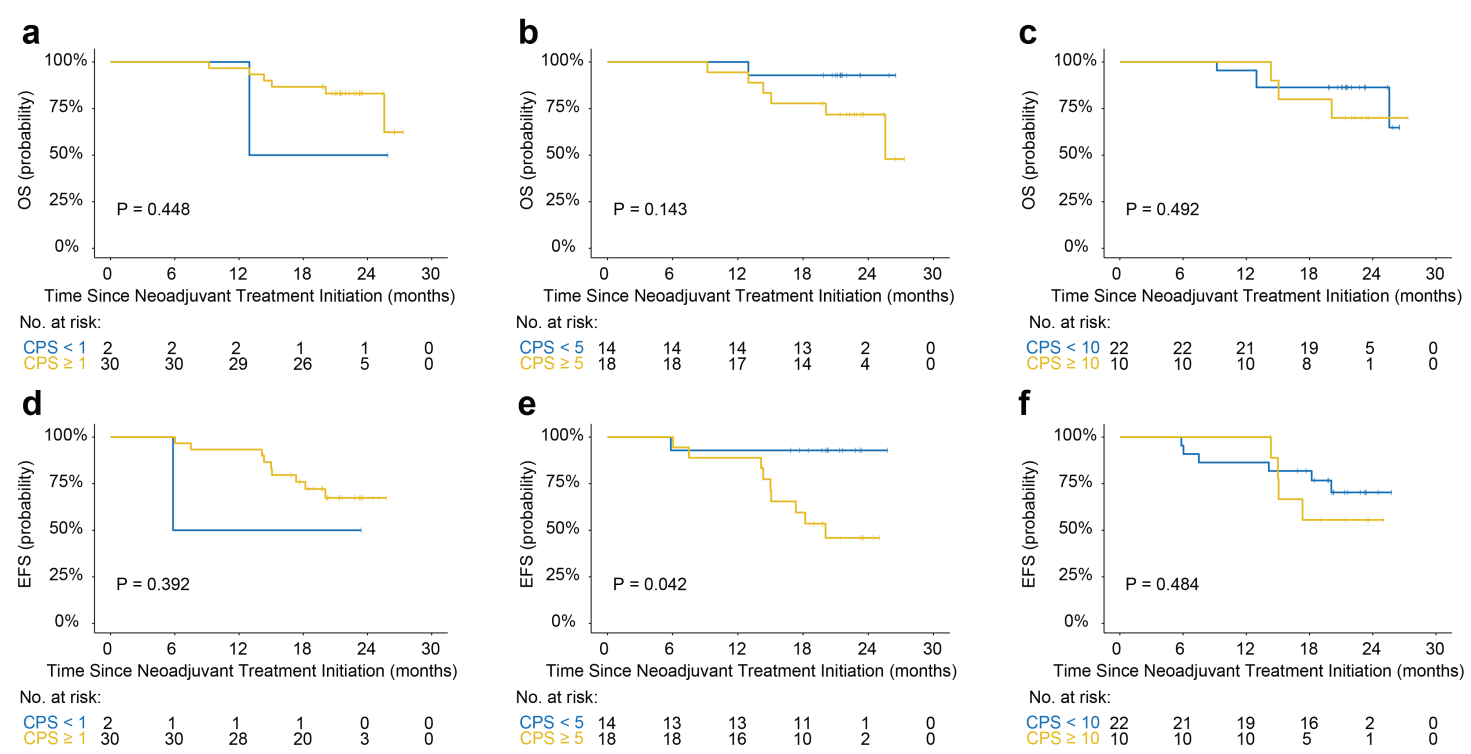
**

**Supplementary Fig. 2 Kaplan-Meier plots for OS and EFS stratified by PD-L1 CPS.** (a-c) OS according to PD-L1 CPS cutoffs of 1 (a), 5 (b), and 10 (c); (d-f) EFS according to PD-L1 CPS cutoffs of 1 (d), 5 (e), and 10 (f). OS, overall survival; EFS, event-free survival; PD-L1, programmed cell death-ligand 1, CPS, combined positive score.
