# Supplementary material for: Perioperative tislelizumab plus chemotherapy for locally advanced gastroesophageal junction adenocarcinoma (NEOSUMMIT-03): a prospective, nonrandomized, open-label, phase 2 trial
Source: Signal Transduct Target Ther. 2025 Feb 5;10:60. doi: 10.1038/s41392-025-02160-8 (PMC11799164; doi:10.1038/s41392-025-02160-8)
Supplement: Supplementary file 2 — Protocol [file 41392_2025_2160_MOESM2_ESM.docx]

**Perioperative chemotherapy plus PD-1 antibody in locally advanced Siewert II/III type of gastro-esophageal junction adenocarcinoma: an open-label, phase 2 trial**

**Study Protocol**

**Principal Investigators: Yingbo Chen**

**Sponsor: Sun Yat-sen University Cancer Center**

**Version Date: March 29, 2023**

**Version No: 2.2**

**Synopsis**

| Title | Perioperative chemotherapy plus PD-1 antibody in locally advanced Siewert II/III type of gastro-esophageal junction adenocarcinoma: an open-label, phase 2 trial |
| --- | --- |
| Clinical Research Unit | Sun Yat-sen University Cancer Center |
| Study Phase | Prospective, open-label, single arm clinical trial |
| **Study Objectives** | **Primary Objective**   - To evaluate the safety and anti-tumor activity of perioperative chemotherapy plus PD-1 antibody in locally advanced Siewert II/III type of gastro-esophageal junction adenocarcinoma   **Exploratory Objectives**   - To evaluate the correlation between programmed death-ligand 1 (PD-L1) expression, Epstein-Barr virus (EBV) expression and microsatellite instability (MSI) status in tumor tissue and anti-tumor response - To evaluate the correlation between ctDNA in blood and the anti-tumor activity of chemotherapy plus PD-1 antibody in locally advanced Siewert II/III type of gastro-esophageal junction adenocarcinoma - To explore the correlation between gene mutation, RNA expression and proteomics in tumor tissue and the anti-tumor activity - To explore other potential immune-related predictors of anti-tumor response |
| **Study Endpoints** | **Primary Endpoint**   - Major Pathological regression defined as residual tumor cells ≤10%   **Secondary Endpoints**   - Pathological complete response (pCR) - Pathological complete regression/moderate regression rate (TRG 0/1) - R0 resection rate - Objective response rate (ORR) as assessed by Response Evaluation Criteria in Solid Tumors (RECIST 1.1) - Recurrence-free survival (RFS) - Event-free survival (EFS) - Overall survival (OS) - Safety: incidence and severity of adverse events, clinically significant abnormal laboratory findings; surgical complications occurring within 30 days after surgery - Quality of Life evaluation including European Organization for Research and Treatment of Cancer Quality of Life Questionnaire-Core 30 Score (EORTC QLQ-C30 (version 3)); European Organization for Research and Treatment of Cancer Quality of Life Questionnaire Gastric Cancer Module QLQ-STO22 Score (EORTC QLQ-STO22) and European Quality of Life 5-Dimensions 5-Levels Health Questionnaire Score (EQ-5D-5L) |
| **Study Design** | **Overall Design:**  This study is a prospective, single arm Phase 2 clinical trial of perioperative chemotherapy plus PD-1 antibody Tislelizumab in locally advanced Siewert II/III type of gastro-esophageal junction adenocarcinoma. Eligible subjects will be selected based on the inclusion and exclusion criteria: i.e., treatment-naive patients with locally advanced Siewert II/III type of gastro-esophageal junction adenocarcinoma (cT1-2N+M0, Ct3-4aNanyM0). Subjects will receive preoperative chemotherapy plus PD-1 antibody Tislelizumab, followed by standardized radical gastrectomy (D2 lymph node dissection), and finally receive adjuvant chemotherapy plus Tislelizumab.  **Study Treatments:**  1) 3 courses of neoadjuvant chemotherapy of SOX + PD-1 antibody Tislelizumab administered once every 3 weeks (Q3W); 2) tumor response assessment after 3 courses of neoadjuvant therapy; 3) conventional surgery; 4) 5 courses of adjuvant chemotherapy of XELOX/SOX + PD-1 antibody Tislelizumab 4-6 weeks after surgery, and Tislelizumab treatment may be continued for 1 year as appropriate.  The PD-1 antibody treatment may be continued if the subject can still benefit from the treatment after disease progression at the discretion of the investigator, with permission from the investigator and the sponsor, or the medical monitor of the sponsor-authorized Contract Research Organization (CRO), but the original chemotherapy must be terminated. If a subject experiences re-progression after continuing investigational product treatment, he/she should withdraw from the study permanently.  **Tumor Assessment:**  During the study, tumor response will be assessed according to RECIST 1.1 and iRECIST after 3 courses of neoadjuvant therapy, every 3 months for the first 2 years after surgery, every 6 months for the first 3-5 years, and yearly thereafter for tumor recurrence until disease recurrence, death, or loss to follow-up (whichever occurs first).  **Acquisition of Tumor Tissue Specimens:**  Subjects must provide eligible tumor tissue specimens before enrollment (fresh tumor tissue specimens biopsied before enrollment are preferred) for use in subsequent exploratory studies.  The subject consents to the investigator to obtain the surgical tissue specimen for the subsequent exploratory study.  Subjects with response evaluation as progressive disease (PD) may be encouraged to voluntarily participate in the optional biomarker study if tumor lesion tissue is available and provide tumor tissue for exploratory study on the correlation between tumor markers and anti-tumor response.  **End of Study:**  The study will be ended at 12 months after enrolling the last subject and the final analysis will be performed. |
| **Inclusion Criteria** | Patients enrolled in the study must meet all of the following criteria:   1. Fully informed about the study and voluntarily sign an informed consent form (ICF); 2. Histologically CT/MRI confirmed clinical stage II/III (cT1-2N+M0 or cT3-4aNanyM0) Siewert II/III type of gastro-esophageal junction adenocarcinoma; 3. Consent to send previously-stored tumor tissue or perform a biopsy to collect tumor lesion tissues for PD-L1, EBV and MSI immunohistochemistry (IHC) detection at the central laboratory; 4. Men or women, 18-75 years; 5. Good general condition with ECOG 0-1, and no surgery contraindications; 6. Physical condition and adequate organ function to ensure the success of abdominal surgery; 7. Expected survival ≥ 3 months; 8. Laboratory values must meet the following criteria within 7 days prior to enrollment: 9. WBC > 4.0 × 10^9^/L and < 15 × 10^9^/L, ANC > 1.5 × 10^9^/L, Hb ≥ 90 g/L, PLT ≥ 100 × 10^9^/L; 10. Serum bilirubin ≤ 1.5 × upper limit of normal, AST and ALT ≤ 2.5 × upper limit of normal; 11. Creatinine ≤ 1.5 × upper limit of normal or serum clearance > 60 ml/min, based on the estimated glomerular filtration rate by Cockcroft-Gault:   (140 − age) × (weight in kg) × (0.85 if female)  72 × (serum creatinine, mg/dl)  or:  (140 − age) × (weight in kg) × (0.85 if female)  0.818 × (serum creatinine, μmol/l)   1. INR and aPTT ≤ 1.5 × ULN only for subjects not receiving anticoagulant therapy; Subjects receiving anticoagulant therapy should be administrated at a stable dose 2. Have good compliance, and cooperate with the laboratory, auxiliary examination and corresponding specimen collection specified in this protocol; 3. Females of childbearing potential (including those who are chemically or medically postmenopausal) must agree to use contraception from signing the informed consent until at least 5 months after the last dose of study treatment or concomitant chemotherapy (whichever is later). Such females should also agree to not breastfeed from signing the informed consent until at least 5 months after the last dose of the study treatment or concomitant chemotherapy (whichever is later);   Males should agree to use contraception from the time of investigational drug administration until at least 7 months after the administration of the investigational drug or concomitant chemotherapy (whichever is later). |
| **Exclusion Criteria** | Subjects who meet any of the following criteria will be excluded from this clinical study:   1. Known allergy to citric acid monohydrate, sodium citrate dihydrate, mannitol, and polysorbate (components of the investigational drug); 2. Previous or concurrent other malignancies (except completely resected basal cell carcinoma, stage I squamous cell carcinoma, carcinoma in situ, intramucosal carcinoma, superficial bladder cancer or any other cancer that has not recurred for at least 5 years); 3. Uncontrolled pericardial effusion, pleural effusion or ascites, hemorrhage of the digestive tract, or high risk of hemorrhage in 2 weeks before recruitment; 4. Weight loss >20% within 2 weeks before recruitment; 5. Unable to swallow study drug; 6. Having a history of chemotherapy, radiotherapy, immunotherapy or surgical treatment for gastric cancer; 7. Prior therapy with PD-1 inhibitors, PD-L1 inhibitors, PD-L2 inhibitors, or CTLA-4 inhibitors (or other inhibitors in T cell co-stimulatory signals or checkpoint pathways); 8. Prior therapy with tyrosine kinase inhibitor within 2 weeks before recruitment; 9. Concurrent medical condition requiring chronic therapy with immunosuppressive drugs, or systemic or topical corticosteroids at immunosuppressive doses (> 10 mg/day of prednisone or equivalent dose); 10. Have vaccination with any anti-infection vaccine (e.g. influenza vaccine, chickenpox vaccine) within 4 weeks prior to enrollment; 11. Uncontrolled diabetes, hypertension and other systemic diseases; 12. Receiving or requiring anticoagulant therapy (except antiplatelet therapy with low-dose aspirin); 13. With any active autoimmune diseases or history of autoimmune diseases (including but not limited to interstitial pneumonia, uveitis, enteritis, hepatitis, hypophysitis, nephritis, hyperthyroidism, hypothyroidism; subjects with vitiligo or who had a complete response of asthma in childhood and do not require any intervention after adulthood can be included; asthma requiring medical intervention with a bronchodilator cannot be included); 14. Patients with active tuberculosis (TB) who are receiving anti-tuberculosis therapy or have received anti-tuberculosis therapy within 1 year prior to screening; 15. With histories of the following lung diseases confirmed by imaging (preferably CT) or clinical findings: interstitial pneumonia, noninfectious pneumonitis, pulmonary fibrosis, acute lung disease; 16. Contraindication to oxaliplatin or capecitabine or tegafur/gimeracil/oteracil; 17. Pregnant or lactating women or those who may become pregnant; 18. Positive test for HbsAg and HBV-DNA copy numbers (≥ 1000 cps/ml); 19. Positive results for any of the following tests: human immunodeficiency virus-1 (HIV-1) antibodies, human immunodeficiency virus-2 (HIV-2) antibodies, human T-cell lymphotropic virus 1 (HTLV-1) antibodies, or hepatitis C virus (HCV) antibodies; 20. Any other clinically significant disease or condition that, in the opinion of the investigator, may affect the compliance or the signing of the informed consent form (ICF), or is inappropriate for participation in this clinical trial. |
| **Eliminate Criteria** | 1. Concurrent use of other chemotherapy drugs outside the trial protocol during the trial; 2. Those who were not given the drug according to the dose and course specified in the study plan, and those who were given the drug for less than two cycles (non-tumor progression factors), were not counted for the efficacy statistics but were counted for the toxicity and side effects; 3. Other violations of the experimental protocol. |
| **Study Drug, Dose and Route of Administration** | **PD-1 antibody Treatment Regimen and Drug**  Drug: Tislezumab injection (BGB-A317)  Strength: 100 mg/10 ml/vial  Method of administration: Tislezumab is administered at 200 mg once every 3 weeks. The chemotherapy treatment will be started 1 hour after the end of the Tislezumab infusion and close monitoring of vital signs. The patients will be treated with Tislezumab for 3 courses, and then receive routine surgery. After 4-6 weeks, the patients are treated with Tislezumab for 5 courses (a total of 8 courses). Tislelizumab treatment may be continued for 1 year as appropriate.  **Chemotherapy Regimens and Drugs**   - **SOX regimen:**   **Oxaliplatin:** 130 mg/m^2^, iv drip over 3 hours on d1, once every 3 weeks;  **Tegafur/gimeracil/oteracil**: initial dose is 40 mg/m^2^/dose (40 mg/dose for body surface area < 1.25 m^2^; 50 mg/dose for body surface area of 1.25 to 1.5 m^2^; 60 mg/dose for body surface area ≥ 1.5 m^2^). It will be administered bid, p.o on d1~14, once every 3 weeks (refer to the package insert).  Each cycle consists of 21 days. The patients will be treated with SOX chemotherapy for 3 courses, and then receive routine surgery. After 4-6 weeks, the patients will receive SOX chemotherapy for 5 courses, until disease recurrence, any unacceptable toxicity, withdrawal of consent, or treatment discontinuation.  Tegafur/gimeracil/oteracil will be considered not administered if a patient vomits within 30 minutes after dosing, and it will be recorded in the patient diary card and eCRF. Patients who experience vomiting must not receive replacement therapy. Tegafur/gimeracil/oteracil should not be administered more frequently than twice daily in any case. |
| **Specific Efficacy and Evaluation** | The investigator will evaluate the histopathological changes of the surgical specimens from the subjects, including Major Pathological regression (MPR); Pathological complete response (pCR); tumor regression grade (TRG) and R0 resection; The tumor response based on the imaging examination will be evaluated per RECIST 1.1, including objective response rate (ORR); the 1-, 3-, and 5-year EFS, RFS and OS will be calculated. |
| **Safety Evaluation** | Safety evaluation mainly includes monitoring and recording adverse events (AEs) and serious adverse events (SAEs), laboratory tests (including hematology, blood chemistry and urinalysis) specified in the protocol, 12-lead electrocardiogram, and vital signs.  All subjects will return to the hospital within 30 days after the last dose of the study drug for an End of Study/Early Termination Visit. During the study, all adverse events and serious adverse events will be recorded until 60 days after the last dose of the study drug or initiation of new anticancer therapy, whichever occurs first. Thereafter, the investigator will be asked to report all serious adverse events related to the study treatment, regardless of whether they occur after the study medication. In addition, if there is an unresolved adverse event or abnormal laboratory finding that is considered related to study treatment, the subject will continue to be followed until the event is resolved or returns to baseline and is stable assessed by the investigator, subject is lost to follow-up, withdraws consent, or it is determined that adverse event is not attributed to study treatment or study participation. Adverse events will be graded based on the National Cancer Institute Common Terminology Criteria for Adverse Events version 5.0 (NCI CTCAE V5.0).  According to the Clavien-Dindo Classification of Complications, the investigator will evaluate the surgical complications within 30 days after surgery, mainly including hemorrhage of the digestive tract, anastomotic leakage, pancreatic fistula and incision complications (including infection, bleeding and dehiscence). |
| **Sample Size** | The main study endpoint is major pathological regression rate (MPR). According to the previous study results and the practical experience of Sun Yat-sen University Cancer Hospital, the MPR rate in the history control group is about 13% and is expected to reach 33% in the group, with Alpha (α) of 0.025 (one-sided) and test power of 0.80. The sample size calculation formula for the a single sample rate shows that 28 subjects are needed for each group. Considering the drop-out rate of 10%, it is expected that 32 subjects will be included in total. |
| **Analysis Sets** | **Full analysis set:** Subjects who have received at least one perioperative treatment and have follow-up data after medication.  **Efficacy evaluable analysis set:** Subjects who have received at least one perioperative treatment and had baseline tumor evaluation data and at least one post-baseline tumor evaluation data.  **Safety analysis set:** Subjects who have received at least one perioperative treatment. This analysis set will be used for safety analysis.  **Per protocol analysis set:** Subjects included in the efficacy evaluable analysis set with no significant protocol violations. The dataset included subjects who were in good compliance, did not take any prohibited drugs during the trial, and completed the trial as required. |
| **Efficacy Analysis** | The primary efficacy endpoint in this study is major pathological regression rate (MPR). The efficacy analysis will be mainly based on the full analysis set and the efficacy evaluable analysis set. A Chi-square test method is used to compare the proportions of MPR and the 95% confidence intervals for the difference between the test group and the history control group. |
| **Safety Analysis** | The safety analysis set will be used for safety analyses. Safety endpoints are detailed in Protocol Section 8. The safety of Tisleizumab will be evaluated by summarizing the type, incidence, severity, outcome, and causal relationship with the investigational drug for AEs and SAEs. The final safety analysis will be performed based on subject safety information collected during the study. Specific statistical methods will be detailed in the statistical analysis plan. |

Table of Contents

[1. Background 19](#_Toc164213301)

[1.1 Introduction 19](#_Toc164213302)

[1.2 Perioperative Chemotherapy for Gastric Cancer 21](#_Toc164213303)

[1.3 Current Status of Adjuvant Chemotherapy for Gastric Cancer 22](#_Toc164213304)

[1.4 Radical Surgery for Gastric Cancer 23](#_Toc164213305)

[1.5 Current Status of PD-1 Inhibitors for Gastric Cancer 23](#_Toc164213306)

[1.6 Study Rationale 27](#_Toc164213307)

[1.6.1 Rationale for Perioperative Chemotherapy Plus PD-1 Antibody in Locally Advanced AEG 27](#_Toc164213308)

[1.6.2 Theoretical Rationale for Collection of Archival and Fresh Tumor Specimen and Biomarker Blood Samples 28](#_Toc164213309)

[2. Study Objectives and Endpoints 28](#_Toc164213310)

[2.1 Primary Objective 29](#_Toc164213311)

[2.2 Exploratory Objectives 29](#_Toc164213312)

[2.3 Primary Endpoint 29](#_Toc164213313)

[2.4 Secondary Endpoints 29](#_Toc164213314)

[3. Study Design 30](#_Toc164213315)

[3.1 Overall Design 30](#_Toc164213316)

[3.2 Study Method 32](#_Toc164213317)

[3.2.1 PD-1inhibitor Treatment Regimen and Drug 32](#_Toc164213318)

[3.2.2 Chemotherapy Regimens and Drugs 33](#_Toc164213319)

[3.2.3 End of Study 33](#_Toc164213320)

[4. Study Population 33](#_Toc164213321)

[4.1 Inclusion Criteria 33](#_Toc164213322)

[4.2 Exclusion Criteria 35](#_Toc164213323)

[4.3 Eliminate Criteria 36](#_Toc164213324)

[4.4 Discontinuation Criteria 37](#_Toc164213325)

[4.5 Handling of Treatment Discontinuation 38](#_Toc164213326)

[5. Schedule of Activities 38](#_Toc164213327)

[5.1 Screening/Baseline Period (Day -28 to Day -1) 38](#_Toc164213328)

[5.2 Preoperative and Postoperative Treatment Period Visits 41](#_Toc164213329)

[5.3 Operative Period 42](#_Toc164213330)

[5.4 End-of-Treatment Visit 43](#_Toc164213331)

[5.5 Treatments after Tumor Recurrence/Metastasis 43](#_Toc164213332)

[5.6 Survival Follow-up 44](#_Toc164213333)

[6. Study Drugs and Study Method 45](#_Toc164213334)

[6.1 Study Drug Supply 45](#_Toc164213335)

[6.1.1 Name: Tislelizumab Injection 45](#_Toc164213336)

[6.1.2 Chemotherapeutic drugs 45](#_Toc164213337)

[6.2 Management of Investigational Drug 45](#_Toc164213338)

[6.2.1 Receipt and Storage 45](#_Toc164213339)

[6.2.2 Disposition 46](#_Toc164213340)

[6.2.3 Preparation Method and Record 46](#_Toc164213341)

[6.3 Medication Considerations 48](#_Toc164213342)

[6.4 Concomitant Medications and Concurrent Treatments 49](#_Toc164213343)

[6.5 Chemotherapy Dose Modifications 50](#_Toc164213344)

[6.6 Overdose 51](#_Toc164213345)

[7. Biomarker Tests 51](#_Toc164213346)

[8. Safety Assessments 52](#_Toc164213347)

[8.1 Overall Plan to Manage Tirilizumab Safety Issues 52](#_Toc164213348)

[8.1.1 Monitoring 53](#_Toc164213349)

[8.1.2 Tirilizumab Dose Modification 53](#_Toc164213350)

[8.1.3 Management of Special Adverse Events 54](#_Toc164213351)

[8.2 Chemotherapy Safety Management and Dose Modification 58](#_Toc164213352)

[8.2.1 Dose Modification of SOX Regimen 58](#_Toc164213353)

[8.3 Safety Parameters and Definitions 62](#_Toc164213354)

[8.3.1 Definition of Adverse Events (AEs) 63](#_Toc164213355)

[8.3.2 Laboratory Abnormalities 64](#_Toc164213356)

[8.3.3 Serious Adverse Events (SAEs) 64](#_Toc164213357)

[8.4 Causality Assessment 66](#_Toc164213358)

[8.5 Assessment of Severity 67](#_Toc164213359)

[8.6 Recording and Reporting of AEs 68](#_Toc164213360)

[8.6.1 Recording and Reporting of AEs 68](#_Toc164213361)

[8.6.2 Reporting of SAEs 69](#_Toc164213362)

[8.6.3 Reporting and Follow-up of Pregnancies 70](#_Toc164213363)

[8.7 Recording Procedures for AEs 70](#_Toc164213364)

[8.7.1 Infusion-related Adverse Reactions 70](#_Toc164213365)

[8.7.2 Diagnosis versus Signs and Symptoms 71](#_Toc164213366)

[8.7.3 Adverse Events Secondary to Other Events 71](#_Toc164213367)

[8.7.4 Persistent or Recurrent Adverse Events 72](#_Toc164213368)

[8.7.5 Abnormal Laboratory Values 72](#_Toc164213369)

[8.7.6 Abnormal Vital Sign Values 73](#_Toc164213370)

[8.7.7 Abnormal Liver Function Test 74](#_Toc164213371)

[8.7.8 Death 74](#_Toc164213372)

[8.7.9 Pre-existing Diseases 75](#_Toc164213373)

[8.7.10 Lack of Efficacy 75](#_Toc164213374)

[8.7.11 In-patient Hospitalization or Prolongation of Existing Hospitalization 75](#_Toc164213375)

[8.7.12 Adverse Events Associated with Overdose or Drug Administration Errors 76](#_Toc164213376)

[9. Data Management 76](#_Toc164213377)

[9.1 Data Entry 76](#_Toc164213378)

[9.2 Database Lock 77](#_Toc164213379)

[10. Statistical Analysis 77](#_Toc164213380)

[10.1 Calculation of Sample Size 77](#_Toc164213381)

[10.2 Data Analysis Sets 78](#_Toc164213382)

[10.3 Analytical Methods 78](#_Toc164213383)

[10.3.1 Safety 78](#_Toc164213384)

[10.3.2 Efficacy 79](#_Toc164213385)

[10.3.3 Exploratory Analysis 80](#_Toc164213386)

[10.4 Interim Analysis 81](#_Toc164213387)

[11. Study Management 81](#_Toc164213388)

[11.1 Ethical Considerations 81](#_Toc164213389)

[11.2 Informed Consent 81](#_Toc164213390)

[11.3 Compensation for Health Damage 82](#_Toc164213391)

[11.4 Recording and Retention of Study Data 82](#_Toc164213392)

[11.5 Return or Destruction of Study Drugs/Therapeutic Supplies 82](#_Toc164213393)

[11.6 Quality Control and Quality Assurance 83](#_Toc164213394)

[11.6.1 Monitoring and Audit 83](#_Toc164213395)

[11.7 Amendments to the Study Protocol 84](#_Toc164213396)

[11.8 Protocol Violation 84](#_Toc164213397)

[11.9 Study Termination 84](#_Toc164213398)

[11.10 Study Summary Report 85](#_Toc164213399)

[11.11 Confidentiality and Publication of Study Results 85](#_Toc164213400)

[12. References 85](#_Toc164213401)

[Appendix 1 AJCC/UICC TNM Staging Criteria Version 8 for Gastric Cancer 88](#_Toc164213402)

[Appendix 2 CT Staging of Gastric Cancer 90](#_Toc164213403)

[Appendix 3 NCI Response Evaluation Criteria in Solid Tumors (RECIST Version 1.1) 91](#_Toc164213404)

[Appendix 4 Schedule of Activities (SOA) 94](#_Toc164213405)

[Appendix 5 Patient Performance Status (ECOG) Scoring Criteria 97](#_Toc164213406)

[Appendix 6 Evaluation of Preoperative Adjuvant Therapy for Tumor (Tumor Regression Grade) 98](#_Toc164213407)

[Appendix 7 Clavien-Dindo Classification of Complications 98](#_Toc164213408)

[Appendix 8 Recommendations for Replacement Therapy for Hypothyroidism 99](#_Toc164213409)

[Appendix 9 Blood Volume Estimation 100](#_Toc164213410)

1. Background

## 1.1 Introduction

Gastric cancer (GC) is a worldwide serious health problem, being one of the most common malignant tumors worldwide, with the fifth incidence (fourth in men and seventh in women), the third mortality (third in men and fifth in women), and more than 782,685 gastric cancer deaths in 2018 ^[^[^1^](#_ENREF_1)^]^.About 1/2 of gastric cancer in the world occurs in China, and gastric cancer is the second leading cause of cancer death in China ^[^[^2^](#_ENREF_2)^]^.

The incidence has significant regional differences. It is most common in East Asia: accounting for almost 60% of all new cases and 56% of deaths each year in Korea, Japan, and China. In the world, the incidence of gastric cancer is highest in Japan, with an age-standardized mortality rate of 69.20 per 100,000 cases in men and 28.64 per 100,000 cases in women. It is high in China and the population-adjusted mortality rate in men and women in China (men: 27.88/100,000, women: 11.55/100,000, 2.4 times that of women) is 4.2 ~ 7.9 times and 3.8 ~ 8.0 times that of developed countries in Europe and the United States, respectively. The overall incidence of gastric cancer in China accounts for about 46% of the world, and there are significant regional and urban-rural differences, with urban of 17.29/100,000, and rural of 22.82/100,000, which is 1.3 times of urban ^[^[^2^](#_ENREF_2)^]^.

From a global perspective, the incidence of gastric cancer has been decreasing year by year ^[^[^3^](#_ENREF_3)^]^. However, accompanying this trend, adenocarcinoma of the esophagogastric junction (AEG) has shown a clear upward trend in its proportion relative to gastric cancer including in some East Asian countries such as China ^[^[^4^](#_ENREF_4)^]^. Based on global data, it has been found that the majority of new AEG cases occur in East Asia and Southeast Asia, accounting for 59% of the total global incidence ^[^[^5^](#_ENREF_5)^]^.

The esophagogastric junction refers to a virtual anatomical line where the esophagus and stomach meet. It is also referred to as the "z-line" or anatomically known as the "cardia." From an anatomical perspective, this is the area that connects the esophagus and stomach. From a histological perspective, it is the area where the squamous epithelium of the esophagus transitions into the glandular epithelium of the stomach. Adenocarcinoma occurring in this area is called adenocarcinoma of the esophagogastric junction (AEG). According to the World Health Organization's definition, AEG refers to adenocarcinomas with tumor centers within 5 centimeters above or below the esophagogastric junction, crossing or involving the junction between the esophagus and stomach. Currently, it is widely recognized that AEG has anatomical and biological characteristics distinct from gastric cancer and esophageal cancer. For example, different subtypes of AEG have significantly different probabilities of lymph node metastasis in the mediastinum and abdominal cavity, which greatly impacts the surgical approach ^[^[^6^](#_ENREF_6)^,^ [^7^](#_ENREF_7)^]^. Therefore, an increasing number of experts and scholars believe that AEG should be regarded as a separate cancer ^[^[^8^](#_ENREF_8)^]^。

Currently, the classification of AEG is based on the Siewert classification proposed by German scholar Siewert in 1987 which is the also widely used classification system internationally for AEG. It mainly consists of three types: Type I, with the tumor center located within 1 to 5 centimeters above the Z-line; Type II, with the tumor center located within 1 centimeter above the Z-line to 2 centimeters below the Z-line; and Type III, with the tumor center located within 2 to 5 centimeters below the Z-line. However, there are still ongoing debates and lack of consensus regarding the staging, classification, and treatment modalities for AEG. Previous studies have often included AEG as part of esophageal or gastric cancer research, so the National Comprehensive Cancer Network (NCCN) in the United States recommends referring to the staging and treatment modalities for esophageal or gastric cancer for AEG ^[^[^9^](#_ENREF_9)^]^. Specifically, for Siewert Type I AEG, the recommended treatment approach is based on esophageal cancer, while for Siewert Type II/III AEG, the recommended treatment approach is based on gastric cancer.

It is well known that surgery is currently the only possible cure option for gastric cancer. Due to the lack of large-scale gastric cancer screening like in Japan and South Korea, the proportion of advanced gastric cancer in China is about 80-90%, **^[^**[**^10^**](#_ENREF_10)**^]^** so the treatment level and strategy for gastric cancer in China are different from those in Japan and South Korea. Therefore, for locally advanced Siewert II/III type of gastro-esophageal junction adenocarcinoma (AEG), how to downstage to improve the surgical resection rate or radical resection rate, thereby improving patient survival rate, is a severe topic for gastric cancer researchers and clinicians.

## 1.2 Perioperative Chemotherapy for Gastric Cancer

Considering that radical surgery is difficult and risky for patients with locally advanced gastric cancer, and surgery including extended surgery is limited for controlling micrometastatic lesions, patients with locally advanced gastric cancer have a high postoperative recurrence rate and poor prognosis. In recent years, domestic and foreign scholars have been exploring the significance of perioperative chemotherapy in locally advanced gastric cancer. Theoretically, preoperative neoadjuvant chemotherapy can verify chemosensitivity, shrink tumor lesions, downstage, improve R0 resection rate, control micrometastases, reduce postoperative recurrence and metastasis, and prolong DFS and OS.

The MAGIC study ^[^[^11^](#_ENREF_11)^]^ adopted a new treatment regimen of perioperative neoadjuvant chemotherapy combined with adjuvant chemotherapy for advanced gastric cancer. Subjects in this study received an ECF regimen (epirubicin + cisplatin + 5-FU) for 3 cycles, then received surgery and 3 cycles of ECF chemotherapy regimen after surgery. Compared with surgery alone, 5-year DFS and 5-year OS were significantly prolonged in the combined treatment group, with a 34% decrease in the risk of recurrence-free metastasis (HR 0.66, P < 0.001) and a 25% decrease in the risk of death (HR 0.75, P = 0.009). The FFCD study ^[^[^12^](#_ENREF_12)^]^ further validated the superiority of perioperative chemotherapy over surgery alone. In this study, patients received 2-3 cycles of the PLF regimen (cisplatin combined with 5-FU) before surgery, followed by surgery and an additional 3-4 cycles of PLF chemotherapy after surgery. Compared to the surgery alone group, the perioperative chemotherapy group had a higher R0 resection rate (84% vs. 74%, P = 0.04), longer 5-year DFS (34% vs. 19%, P = 0.003), and 5-year OS (38% vs. 24%, P = 0.02).

Both of these results demonstrate that neoadjuvant chemotherapy for locally advanced gastric cancer can reduce T and N staging, and improve the radical surgery rate; perioperative chemotherapy can improve the long-term survival rate for patients with advanced gastric cancer. Therefore, the NCCN guidelines recommend cT2-4aNanyM0 patients with advanced gastric cancer receive perioperative chemotherapy as a level 1 recommendation. Although the MAGIC study and the FFCD study became successful precedents for the perioperative treatment of locally advanced gastric cancer, no more than 50% of patients in the test group completed all postoperative adjuvant chemotherapy (MAGIC: 42%; FFCD: 37.6%), and the 5-year survival rate in the control group was much lower than that in the same population from Asian countries. Therefore, these two studies have been criticized by Oriental scholars. For gastric cancer patients with local invasion and lymph node metastasis, how to provide surgery-based comprehensive treatment is still unresolved.

## 1.3 Current Status of Adjuvant Chemotherapy for Gastric Cancer

Considering the positive efficacy of chemotherapy for advanced gastric cancer compared with the best supportive care, many relevant studies (including randomized controlled studies and meta-analyses) have been conducted in European and American and Asian countries to investigate the value of adjuvant chemotherapy after radical resection for locally advanced gastric cancer. The results showed that adjuvant chemotherapy had a survival benefit compared with surgery alone. The most classic randomized clinical trials are the ACTS-GS study and the Classic study.

The ACTS-GS study ^[^[^13^](#_ENREF_13)^]^ was a phase 3 clinical trial conducted in Japan involving over 100 clinical centers. The results were published in the New England Journal of Medicine in 2007. ACTS-GS enrolled 1059 patients who were randomly assigned to receive S-1 orally at a standard dose for 12 months or radical surgery alone. The 5-year results of the ACTS-GS study showed that the overall survival rates were 71.7% and 61.1% in the S-1 adjuvant treatment group and surgery alone group, respectively, indicating that S-1 adjuvant chemotherapy can significantly reduce the relative risk of death by 33% in patients with locally advanced gastric cancer after curative resection compared with curative surgery alone, which is one of the most important advances in the treatment of gastric cancer in the past 50 years. The ACTS-GC study also showed a 35% reduction in the relative risk of recurrence and a 12% improvement in the 5-year recurrence-free survival in the S-1 group (65.4% vs. 53.1% in the surgery alone group). The most common Grade 3 or 4 side effects in the S-1 group were nausea, vomiting, diarrhea, appetite absence, and hematologic symptoms, all occurring in ≤ 6% of patients and were well tolerated.

The Classic Study ^[^[^14^](#_ENREF_14)^]^ was a randomized phase 3 clinical trial that included patients with stage II/IIIA/IIIB gastric cancer who underwent D2 radical surgery. A total of 1035 patients were enrolled in the study and received D2 radical surgery. They were randomly assigned to XELOX adjuvant chemotherapy group and the surgery alone group. Patients in the XELOX adjuvant chemotherapy group received a total of 8 cycles of XELOX chemotherapy (6 months). The median follow-up period was 62.4 months, and the 5-year DFS was 68% and 53% in the two groups, respectively (HR = 0.58, P < 0.0001). This survival advantage was shown in patients with stage II, IIIA, and IIIB disease. The 5-year OS was 78% and 69%, respectively (HR = 0.66, P = 0.0015), which confirmed that adjuvant chemotherapy with XELOX significantly reduces the risk of postoperative recurrence and prolongs DFS, thereby prolonging the overall survival. This study further established the value of XELOX regimen as a standard postoperative adjuvant chemotherapy for patients with stage II or III gastric cancer.

## 1.4 Radical Surgery for Gastric Cancer

At present, surgical treatment is the main option for gastric cancer. Ib or above gastric cancer requires surgical treatment, except for some early gastric cancers (Ia) with lesions less than 3 cm and well-differentiated lesions that can undergo endoscopic intramucosal cancer resection. In 2010, DUTCH study ^[^[^15^](#_ENREF_15)^]^ a large phase 3 clinical trial, suggested that D2 surgery could effectively reduce the incidence of tumor-related death after surgery for advanced gastric cancer compared with D1 surgery. In addition, the JCOG9501 study ^[^[^16^](#_ENREF_16)^]^ also suggested that prophylactic D2 + surgery did not improve overall survival compared with D2 surgery. So far, Eastern and Western scholars tend to have a consistent view in the understanding of lymph node dissection methods for advanced gastric cancer; At present, D2 surgery has been used as the standard surgical procedure for locally advanced gastric cancer in Eastern countries and is initially recommended in most Western countries. It requires adequate resection distance and standard regional lymph node dissection. In addition, for the number of resected lymph nodes, D2 surgery requires that the number should not be less than 15.

## 1.5 Current Status of PD-1 Inhibitors for Gastric Cancer

Due to the high complexity, diversity, and variability of tumor biology, it has become a huge challenge for scientists to understand the mechanism of tumor development and find the treatments. For a long time, the treatment route for tumors mainly follows surgery-radiotherapy-chemotherapy-disease deterioration-radiotherapy-chemotherapy, which is currently a relatively mature treatment procedure agreed upon by the medical community. However, there are some shortcomings in these treatment schemes. Surgical resection not only causes great damage to the patient's body during the operation but also is difficult to completely achieve the goal of radical cure after malignant metastasis occurs. Tumor escape variants generated after chemoradiotherapy usually can tolerate further chemoradiotherapy, and develop rapidly, accelerating the death of patients. In addition, some targeted therapies targeting key receptors, genes, regulatory molecules in the process of tumor development can achieve a certain degree of high efficacy and low toxicity. However, targeted drugs only have an effect on tumors with specific mutant genotypes, which means that some tumors cannot be effectively treated by targeted drugs, and still have a high incidence of adverse reactions; At the same time, the long-term therapeutic effects can be reduced when there are also tumor gene mutations, or drug resistance occurs due to the heterogeneity of tumor tissue. Most patients treated with targeted therapy develop drug resistance 6 to 9 months after treatment. Only a few patients can maintain drug resistance for more than a year.

Scientists explored antibody therapy and cytokine therapy from the 1970s to the 1980s. Among them, the discovery of immune checkpoints CTLA-4 and PD-1 is a breakthrough in tumor therapy in the past 10 years ^[^[^17-19^](#_ENREF_17)^]^, especially the discovery of PD-1-PD-L1 signaling pathway and the emergence of inhibitors against PD-1 and PD-L1 are changing the current treatment strategies for cancer. In recent years, PD-1 inhibitors have also shown good efficacy in advanced gastric cancer.

KEYNOTE-012 is a global multicenter, single-arm phase 1b clinical trial that enrolled 3 cohorts: advanced gastric cancer, urothelial cancer, and triple-negative breast cancer ^[^[^20^](#_ENREF_20)^]^. Thirty-six patients with PD-L1-positive (PD-L1 expression by immunohistochemistry in at least 1% of tumor cells or lymphocytes) advanced or recurrent gastric cancer were enrolled in the advanced gastric cancer cohort and treated with pembrolizumab. The results showed that 8 (22%) patients achieved tumor response (ORR) and 5 (13%) patients developed Grade 3-4 treatment-related adverse events.

KEYNOTE-059 ^[^[^21^](#_ENREF_21)^]^ is a global multicenter single-arm phase 2 clinical trial to further validate the efficacy of pembrolizumab in patients with advanced gastric cancer. The sample size was expanded to 259 in this study. The results showed that the ORR of pembrolizumab in patients with advanced gastric cancer was 11.6% and 15.5% in PD-L1-positive patients. Based on this study, the US FDA approved pembrolizumab as a second-line treatment for PD-L1-positive advanced gastric cancer.

ATTRACTION-2 was a double-blind phase 3 randomized controlled trial conducted in 49 centers in Japan, Korea, and Taiwan (China) ^[^[^22^](#_ENREF_22)^]^. A total of 493 patients with PD-L1-positive advanced gastric or esophagogastric junction cancer who had failed 2 or more lines of therapy were enrolled in the study and randomized in a 2:1 ratio to nivolumab or placebo group. After a median follow-up of 8.8 months, the nivolumab group showed a higher ORR (11% vs. 0%, P < 0.001) and longer 1-year OS (26.2% vs. 10.9%) compared with the placebo group, with median OS of 5.26 months and 4.14 months, respectively (HR = 0.63, P < 0.0001), which confirmed the value of PD-1 inhibitors in the treatment of advanced gastric cancer with 3 or more lines of treatment. Nivolumab was also approved for the third-line treatment of advanced gastric cancer in Japan.

KEYNOTE-061 was a phase 3 randomized controlled trial ^[^[^23^](#_ENREF_23)^]^ conducted in 148 centers worldwide to compare the efficacy of pembrolizumab and paclitaxel in the second-line treatment of advanced gastric cancer. The primary study endpoints were OS and PFS in PD-L1-positive patients. A total of 592 patients with advanced gastric cancer who failed first-line therapy were enrolled in this study, including 395 PD-L1-positive patients, 196 patients treated with pembrolizumab, and 199 patients treated with paclitaxel monotherapy. After a median follow-up of 7.9 months, the pembrolizumab group had a longer median OS (9.1 months vs. 8.3 months; HR = 0.82, 1-sided P = 0.042) and a lower incidence of Grade 3-5 treatment-related adverse events (14% vs. 35%) compared with the paclitaxel monotherapy with no statistical difference. In subgroup analysis, the PD-L1 expression rate was more than 10%, and pembrolizumab treatment showed an advantage (HR = 0.64, 95% CI 0.41 – 1.02), which also suggested the potential value of PD-1 inhibitors in the second-line treatment of advanced gastric cancer, especially for patients with high PD-L1 expression.

CheckMate 649 is a global, multicenter Phase III randomized controlled clinical trial designed to explore the efficacy of nivolumab in combination with chemotherapy and nivolumab in combination with ipilimumab as first-line treatment for advanced gastric cancer. The primary endpoints of the study were overall survival (OS) and progression-free survival (PFS) in patients with a PD-L1 CPS score ≥ 5. In 2021, the result of CheckMate 649 published in The Lancet reported the efficacy of nivolumab in combination with chemotherapy compared to palliative chemotherapy ^[^[^24^](#_ENREF_24)^]^ **.** A total of 1,581 patients were enrolled in the study (789 patients in nivolumab combination with chemotherapy group, 792 patients in palliative chemotherapy group). After a median follow-up of 13.1 months, in the CPS ≥ 5 population, nivolumab in combination with chemotherapy showed longer OS (median OS: 14.4 months compared to 11.1 months; HR: 0.71 [0.59-0.86]) and PFS (median PFS: 7.7 months compared to 6.0 months; HR: 0.68 [0.56-0.81]) compared to chemotherapy alone. In the overall population, nivolumab in combination with chemotherapy also demonstrated better efficacy. This suggests the value of nivolumab in combination with chemotherapy in the first-line treatment of gastric cancer.

ATTRACTION-4 is a multicenter Phase III randomized controlled clinical trial conducted in the Asian population, aiming to explore the efficacy of nivolumab in combination with chemotherapy as first-line treatment for advanced gastric cancer. The primary endpoints of the study were overall survival (OS) and progression-free survival (PFS). The results reported at the 2020 ASCO conference ^[^[^25^](#_ENREF_25)^]^ showed A total of 724 patients were enrolled in the study (362 in each group). Compared to chemotherapy alone, nivolumab in combination with chemotherapy resulted in further improvement PFS (median PFS: 10.45 months compared to 8.34 months; HR: 0.68 [0.51-0.90]), but did not significantly improve OS (median OS: 17.45 months compared to 17.15 months; HR: 0.90 [0.75-1.08]).

ORIENT-16 is a multicenter Phase III randomized controlled clinical trial conducted in the Chinese population, aiming to explore the efficacy of sintilimab in combination with chemotherapy as first-line treatment for advanced gastric cancer. The primary endpoints of the study were overall survival (OS) and progression-free survival (PFS).The results reported at the 2021 ASCO conference ^[^[^25^](#_ENREF_25)^]^ showed the following: A total of 650 patients were enrolled in the study (sintilimab in combination with chemotherapy: 327 patients; chemotherapy alone: 323 patients). Compared to chemotherapy alone, the combination of sintilimab and chemotherapy further improved patients' PFS (median PFS: 5.7 months compared to 5.5 months; HR: 0.58 [0.46-0.74]) and OS (median OS: 15.2 months compared to 12.3 months; HR: 0.76 [0.63-0.94]).

## 1.6 Study Rationale

### 1.6.1 Rationale for Perioperative Chemotherapy Plus PD-1 Antibody in Locally Advanced AEG

Perioperative chemotherapy for gastric cancer has received support from several clinical randomized controlled trials (RCTs) ^[^[^11^](#_ENREF_11)^,^ [^12^](#_ENREF_12)^]^. Platinum-and 5-FU-based regimens are still the standard chemotherapeutic option for perioperative chemotherapy. However, the survival benefits obtained from cytotoxic chemotherapy can reach a plateau due to its chemotherapy resistance, so the efficacy still needs to be further improved. Exposure of the immune system to high levels of tumor antigens reasonably expected tumor cell (TC) killing by cytotoxic chemotherapy, and restoration of tumor-specific T-cell immunity in this setting by inhibiting the PD-L1/PD-1 signaling pathway may receive deeper and more durable responses than standard chemotherapy alone.

In theory, PD-1 inhibitors function by mobilizing tumor-specific T-cell immune responses to achieve anti-tumor activity. Their application in the perioperative treatment of non-advanced tumors may better control micrometastases, thereby providing patients with longer disease-free survival (DFS) or even overall survival (OS). A recent study ^[^[^26^](#_ENREF_26)^]^ on the use of neoadjuvant PD-1 inhibitors in resectable stage I/II/IIIA lung cancer has attracted widespread attention. The study included a group of 21 patients who received two cycles of preoperative nivolumab treatment. The results showed that 20 patients underwent curative surgery, with no delays in surgery due to nivolumab treatment. Pathological results from the surgical specimens of these 20 patients showed a major pathological response (<10% tumor residual) in 9 cases (45%), and the 18-month recurrence-free survival (RFS) was 73%. On the other hand, KEYNOTE-585 is a randomized, double-blind, global multicenter Phase III clinical trial investigating the use of perioperative chemotherapy with or without pembrolizumab as neoadjuvant/adjuvant therapy for gastric cancer. The study is currently enrolling participants. Recent research has shown that PD-1 inhibitors generally have good efficacy in esophageal cancer ^[^[^27-29^](#_ENREF_27)^]^.

The CheckMate 577 study, published in the New England Journal of Medicine in 2021, explored the efficacy of immunotherapy in tumors of the esophagus and gastroesophageal junction. The study enrolled patients with esophageal or gastroesophageal junction tumors who had undergone neoadjuvant chemoradiotherapy, R0 resection, and had residual disease on pathology. A total of 794 patients were enrolled in the study, with a 2:1 randomization to receive postoperative adjuvant nivolumab or placebo. After a median follow-up of 24.4 months, the results showed that compared to the placebo group, patients who received adjuvant nivolumab had a significant prolongation in disease-free survival (median: 22.4 months compared to 11.0 months; HR=0.69, P<0.001). Subgroup analysis demonstrated a benefit in both adenocarcinoma and squamous cell carcinoma, with a trend towards benefit in the adenocarcinoma of the esophagogastric junction (HR=0.87, 95% CI: 0.63-1.21). These findings suggest the effectiveness of PD-1 inhibitor adjuvant therapy in adenocarcinomas of the esophagogastric junction.

In summary, the efficacy of PD-1 inhibitors in adenocarcinomas of the esophagogastric junction (AEG) is worth further exploration. Therefore, in this study, PD-1 inhibitors are introduced into the perioperative treatment of AEG to investigate the safety and anti-tumor activity of perioperative chemotherapy combined with PD-1 inhibitors in locally advanced Siewert II/III-type AEG.

### 1.6.2 Theoretical Rationale for Collection of Archival and Fresh Tumor Specimen and Biomarker Blood Samples

The development of predictive diagnostic assays that can prospectively identify subjects who are likely to respond to PD-1 inhibitor treatment may help to screen out patients who are more likely to benefit from this treatment ^[^[^30^](#_ENREF_30)^,^ [^31^](#_ENREF_31)^]^. Analysis of tumor lesion tissue collected before treatment, at the time of tumor response, surgical resection and/or progressive disease (PD) can help to identify the potential predictors for response and efficacy-related resistance to PD-1 inhibitor treatment. The corresponding conclusions will contribute to the development of methods to improve anti-tumor immunotherapy for patients with malignancies.

This study will also evaluate the potential disease-related biomarkers such as PD-L1, EBV, MSI expression, RNA expression, tumor mutation, proteome to explore their relationship with the anti-tumor response; moreover, it will further evaluate other potential pharmacodynamic indicators based on the latest progress. This study will provide more evidence for investigating the biological activity of PD-1 inhibitor in the human body.

1. Study Objectives and Endpoints

The purpose of this study is to preliminarily evaluate the efficacy and safety of perioperative chemotherapy plus PD-1 antibody in the treatment of locally advanced Siewert II/III type of gastro-esophageal junction adenocarcinoma (cT1-2N+M0, cT3-4aNanyM0), and provide sufficient evidence for further expansion of the sample size to conduct a multicenter phase 3 clinical trial.

## 2.1 Primary Objective

- To evaluate the safety and anti-tumor activity of perioperative chemotherapy plus PD-1 antibody in locally advanced Siewert II/III type of gastro-esophageal junction adenocarcinoma

## 2.2 Exploratory Objectives

- To evaluate the correlation between programmed death-ligand 1 (PD-L1) expression, Epstein-Barr virus (EBV) expression and microsatellite instability (MSI) status in tumor tissue and anti-tumor response
- To evaluate the correlation between ctDNA in blood and the anti-tumor activity of chemotherapy plus PD-1 antibody in locally advanced Siewert II/III type of gastro-esophageal junction adenocarcinoma
- To explore the correlation between gene mutation, RNA expression and proteomics in tumor tissue and the anti-tumor activity
- To explore other potential immune-related predictors of anti-tumor response

## 2.3 Primary Endpoint

- Major Pathological regression (MPR) defined as residual tumor cells ≤10%

## 2.4 Secondary Endpoints

- Pathological complete response (pCR)
- Pathological complete regression/moderate regression rate (TRG 0/1)
- R0 resection rate
- Objective response rate (ORR) as assessed by Response Evaluation Criteria in Solid Tumors (RECIST 1.1)
- Recurrence-free survival (RFS)
- Event-free survival (EFS)
- Overall survival (OS)
- Safety: incidence and severity of adverse events, clinically significant abnormal laboratory findings; surgical complications occurring within 30 days after surgery
- Quality of Life evaluation including European Organization for Research and Treatment of Cancer Quality of Life Questionnaire-Core 30 Score (EORTC QLQ-C30 (version 3)); European Organization for Research and Treatment of Cancer Quality of Life Questionnaire Gastric Cancer Module QLQ-STO22 Score (EORTC QLQ-STO22) and European Quality of Life 5-Dimensions 5-Levels Health Questionnaire Score (EQ-5D-5L)

1. Study Design

## 3.1 Overall Design

This study is a prospective, single arm Phase 2 clinical trial of perioperative chemotherapy plus PD-1 antibody Tislelizumab in locally advanced Siewert II/III type of gastro-esophageal junction adenocarcinoma. Eligible subjects will be selected based on the inclusion and exclusion criteria: i.e., treatment-naive patients with locally advanced Siewert II/III type of gastro-esophageal junction adenocarcinoma (cT1-2N+M0, cT3-4aNanyM0). Subjects will receive preoperative chemotherapy plus PD-1 antibody Tislelizumab, followed by standardized radical gastrectomy (D2 lymph node dissection), and finally receive adjuvant chemotherapy plus Tislelizumab.

Subjects will be treated as followings: 1) 3 courses of neoadjuvant chemotherapy of SOX + PD-1 antibody Tislelizumab administered once every 3 weeks (Q3W); 2) tumor response assessment after 3 courses of neoadjuvant therapy; 3) conventional surgery; 4) 5 courses of adjuvant chemotherapy of SOX + PD-1 antibody Tislelizumab 4-6 weeks after surgery, and Tislelizumab treatment may be continued for 1 year as appropriate.

The PD-1 antibody treatment may be continued if the subject can still benefit from the treatment after disease progression at the discretion of the investigator, with permission from the investigator and the sponsor, or the medical monitor of the sponsor-authorized Contract Research Organization (CRO), but the original chemotherapy must be terminated. If a subject experiences re-progression after continuing investigational product treatment, he/she should withdraw from the study permanently.

During the study, tumor response will be assessed according to RECIST 1.1 (Appendix 3) after 3 courses of neoadjuvant therapy, every 3 months for the first 2 years after surgery, every 6 months for the first 3-5 years, and yearly thereafter for tumor recurrence until disease recurrence, death, or loss to follow-up (whichever occurs first).

Subjects must provide eligible tumor tissue specimens before enrollment (fresh tumor tissue specimens biopsied before enrollment are preferred) blood, stool and saliva specimens before enrollment, and blood specimens during treatment for use in subsequent exploratory studies. The subject consents to the investigator to obtain the surgical tissue specimen for the subsequent exploratory study. The specific specimen collection is shown in the table below.

**Table 1.1 Specimen Collection for Exploratory Studies**

| Sampling | | Time Point | | | | | | |
| --- | --- | --- | --- | --- | --- | --- | --- | --- |
|  |  | Before PD1 treatment | After 3 courses of PD1 and before surgery | Surgery | Day 3-5 after surgery | Month 3 after surgery | Month 6 after surgery | Month 12 after surgery |
| Unstained slides (20 pieces) | PD-L1 | √ |  |  |  |  |  |  |
|  | EBER | √ |  |  |  |  |  |  |
| Feces | | √ | √ |  |  | √ |  |  |
| Saliva | | √ | √ |  |  |  |  |  |
| Blood | Circulating tumor cells, DNA (ctcPD-L1, ctDNA) | √ | √ |  | √ | √ | √ | √ |
|  | Peripheral blood leukocyte WES | √ |  |  |  |  |  |  |
|  | PMBC scRNAseq | √ |  | √ |  |  |  |  |
| Fresh tissue | RNA-seq | √  (Gastroscopy) |  | √  (Macroscopic) |  |  |  |  |
|  | WES | √  (Gastroscopy) |  | √  (Macroscopic) |  |  |  |  |
|  | Proteomics | √  (Gastroscopy) |  | √  (Macroscopic) |  |  |  |  |
|  | scRNA-seq | √  (Gastroscopy) |  | √  (Macroscopic) |  |  |  |  |
|  | Spatial metabolomics | √  (Gastroscopy) |  | √  (Macroscopic) |  |  |  |  |

After the completion of the study, the remaining specimens will be transferred to Guangdong Provincial Center for Harmless Treatment of Living Environment Co., Ltd. for disposal, and it is committed that the research samples will only be used for this study and not for any other purposes.

Subjects with response evaluation as progressive disease (PD) may be encouraged to voluntarily participate in the optional biomarker study if tumor lesion tissue is available and provide tumor tissue for exploratory study on the correlation between tumor markers and anti-tumor response.

## 3.2 Study Method

### 3.2.1 PD-1 inhibitor Treatment Regimen and Drug

**Drug**: Tisleizumab injection (BGB-A317)

**Strength**: 100 mg/10 ml/vial

**Method of administration**: This drug is administered in combination with chemotherapy. Tisleizumab is administered at 200 mg once every 3 weeks. The chemotherapy treatment will be started 1 hour after the end of the Tisleizumab infusion and close monitoring of vital signs. The patients will be treated with Tisleizumab for 3 courses, and then receive routine surgery. After 4-6 weeks, the patients are treated with Tisleizumab for 5 courses (a total of 8 courses).

### 3.2.2 Chemotherapy Regimens and Drugs

- **SOX regimen**:

**Oxaliplatin**: 130 mg/m^2^, iv drip over 3 hours on d1, once every 3 weeks;

**Tegafur/gimeracil/oteracil**: initial dose is 40 mg/m^2^/dose (40 mg/dose for body surface area < 1.25 m^2^; 50 mg/dose for body surface area of 1.25 to 1.5 m^2^; 60 mg/dose for body surface area ≥ 1.5 m^2^). It will be administered bid, p.o on d1~14, once every 3 weeks (refer to the package insert).

Each cycle consists of 21 days. The patients will be treated with SOX chemotherapy for 3 courses, and then receive routine surgery. After 4-6 weeks, the patients will receive SOX chemotherapy for 5 courses, until disease recurrence, any unacceptable toxicity, withdrawal of consent, or treatment discontinuation.

Tegafur/gimeracil/oteracil will be considered not administered if a patient vomits within 30 minutes after dosing, and it will be recorded in the patient diary card and eCRF. Patients who experience vomiting must not receive replacement therapy. Tegafur/gimeracil/oteracil should not be administered more frequently than twice daily in any case.

### 3.2.3 End of Study

The study will be ended 6 months after enrolling the last subject and the final analysis will be performed.

1. Study Population

Treatment-naive patients with locally advanced Siewert II/III type of gastro-esophageal junction adenocarcinoma (cT1-2N+M0, cT3-4aNanyM0) will be selected for this study. Specific inclusion/exclusion criteria are as follows:

## 4.1 Inclusion Criteria

Patients enrolled in the study must meet all of the following criteria:

1. Fully informed about the study and voluntarily sign an informed consent form (ICF);
2. Histologically CT/MRI confirmed clinical stage II/III (cT1-2N+M0 or cT3-4aNanyM0) Siewert II/III type of gastro-esophageal junction adenocarcinoma;
3. Consent to send previously-stored tumor tissue or perform a biopsy to collect tumor lesion tissues for PD-L1, EBV and MSI immunohistochemistry (IHC) detection at the central laboratory;
4. Men or women, 18-75 years;
5. Good general condition with ECOG 0-1, and no surgery contraindications;
6. Physical condition and adequate organ function to ensure the success of abdominal surgery;
7. Expected survival ≥ 3 months;
8. Laboratory values must meet the following criteria within 7 days prior to enrollment:
9. WBC > 4.0 × 10^9^/L and < 15 × 10^9^/L, ANC > 1.5 × 10^9^/L, Hb ≥ 90 g/L, PLT ≥ 100 × 10^9^/L;
10. Serum bilirubin ≤ 1.5 × ULN (upper limit of normal), AST and ALT ≤ 2.5 × ULN;
11. Creatinine ≤ 1.5 × ULN and serum clearance > 60 mL/min, based on the estimated glomerular filtration rate by Cockcroft-Gault:

(140 − age) × (weight in kg) × (0.85 if female)

72 × (serum creatinine, mg/dl)

or:

(140 − age) × (weight in kg) × (0.85 if female)

- 1. (serum creatinine, umol/L)

1. INR and aPTT ≤ 1.5 × ULN only for subjects not receiving anticoagulant therapy; Subjects receiving anticoagulant therapy should be administrated at a stable dose
2. Have good compliance, and cooperate with the laboratory, auxiliary examination and corresponding specimen collection specified in this protocol;
3. Females of childbearing potential (including those who are chemically or medically postmenopausal) must agree to use contraception from signing the informed consent until at least 5 months after the last dose of study treatment or concomitant chemotherapy (whichever is later). Such females should also agree to not breastfeed from signing the informed consent until at least 5 months after the last dose of the study treatment or concomitant chemotherapy (whichever is later);

Males should agree to use contraception from the time of investigational drug administration until at least 7 months after the administration of the investigational drug or concomitant chemotherapy (whichever is later).

## 4.2 Exclusion Criteria

Subjects who meet any of the following criteria will be excluded from this clinical study:

1. Known allergy to citric acid monohydrate, sodium citrate dihydrate, mannitol, and polysorbate (components of the investigational drug);
2. Previous or concurrent other malignancies (except completely resected basal cell carcinoma, stage I squamous cell carcinoma, carcinoma in situ, intramucosal carcinoma, superficial bladder cancer or any other cancer that has not recurred for at least 5 years);
3. Uncontrolled pericardial effusion, pleural effusion or ascites, hemorrhage of the digestive tract, or high risk of hemorrhage in 2 weeks before recruitment;
4. Weight loss >20% within 2 weeks before recruitment;
5. Unable to swallow study drug;
6. Having a history of chemotherapy, radiotherapy, immunotherapy or surgical treatment for gastric cancer;
7. Prior therapy with PD-1 inhibitors, PD-L1 inhibitors, PD-L2 inhibitors, or CTLA-4 inhibitors (or other inhibitors in T cell co-stimulatory signals or checkpoint pathways);
8. Prior therapy with tyrosine kinase inhibitor within 2 weeks before recruitment;
9. Concurrent medical condition requiring chronic therapy with immunosuppressive drugs, or systemic or topical corticosteroids at immunosuppressive doses (> 10 mg/day of prednisone or equivalent dose);
10. Have vaccination with any anti-infection vaccine (e.g. influenza vaccine, chickenpox vaccine) within 4 weeks prior to enrollment;
11. Uncontrolled diabetes, hypertension and other systemic diseases;
12. Receiving or requiring anticoagulant therapy (except antiplatelet therapy with low-dose aspirin);
13. With any active autoimmune diseases or history of autoimmune diseases (including but not limited to interstitial pneumonia, uveitis, enteritis, hepatitis, hypophysitis, nephritis, hyperthyroidism, hypothyroidism; subjects with vitiligo or who had a complete response of asthma in childhood and do not require any intervention after adulthood can be included; asthma requiring medical intervention with a bronchodilator cannot be included);
14. Patients with active tuberculosis (TB) who are receiving anti-tuberculosis therapy or have received anti-tuberculosis therapy within 1 year prior to screening;
15. With histories of the following lung diseases confirmed by imaging (preferably CT) or clinical findings: interstitial pneumonia, noninfectious pneumonitis, pulmonary fibrosis, acute lung disease;
16. Contraindication to oxaliplatin or capecitabine or tegafur/gimeracil/oteracil;
17. Pregnant or lactating women or those who may become pregnant;
18. Positive test for HbsAg and HBV-DNA copy numbers (≥ 1000 cps/ml);
19. Positive results for any of the following tests: human immunodeficiency virus-1 (HIV-1) antibodies, human immunodeficiency virus-2 (HIV-2) antibodies, human T-cell lymphotropic virus 1 (HTLV-1) antibodies, or hepatitis C virus (HCV) antibodies;
20. Any other clinically significant disease or condition that, in the opinion of the investigator, may affect the compliance or the signing of the informed consent form (ICF), or is inappropriate for participation in this clinical trial.

## 4.3 Eliminate Criteria

1. Concurrent use of other chemotherapy drugs outside the trial protocol during the trial;
2. For those who did not administer the drug according to the dose and course specified in the study plan, and those who did not use the drug for less than two cycles (non-tumor progression factors), the efficacy statistics were not made, but the toxicity and side effects were counted;
3. Other violations of the experimental protocol.

## 4.4 Discontinuation Criteria

Subjects have the right to withdraw from the study at any stage of the study. The investigator should ask the subject about the reason for withdrawal from the study and, if possible, ask the subject to return to the study site for a final visit and to be followed up on any unresolved adverse events as much as possible. If the subject fails to attend the visit at the scheduled time, the investigator should make every effort to contact the subject and urge the subject to return to the study site for the corresponding visit as soon as possible. The investigator also has the right to prematurely discontinue the treatment for the following or other reasons:

1. Withdrawal of informed consent by the subject or his/her legally acceptable representative;
2. Progressive disease per RECIST 1.1 criteria, and the investigator judges that the subject cannot benefit from continued treatment of Tisleizumab;
3. Recurrence of disease progression after continued treatment of Tisleizumab;
4. Death;
5. Intolerable toxicity;
6. The study drug is discontinued for more than 56 days due to any reason and the investigator judges that the risk of continued treatment with Tisleizumab outweighs the benefit;
7. General deterioration of health status, or study termination at the discretion of the investigator in the best interest of the subject;
8. Serious protocol violation;
9. Pregnant (if applicable);
10. Lost to follow-up (defined as the subject failing to receive a specified visit and the investigator failing to contact the subject or his/her family for at least 3 times through 2 or more routes within 3 months after the visit).

## 4.5 Handling of Treatment Discontinuation

The reasons for premature discontinuation should be recorded in the original medical records and eCRF. The subsequent anticancer therapy and survival status after discontinuation should also be recorded in the original medical records and eCRF.

1. Schedule of Activities

The flow chart is shown in the figure below, and the specific Schedule of Activities (SOA) is shown in Appendix 4.


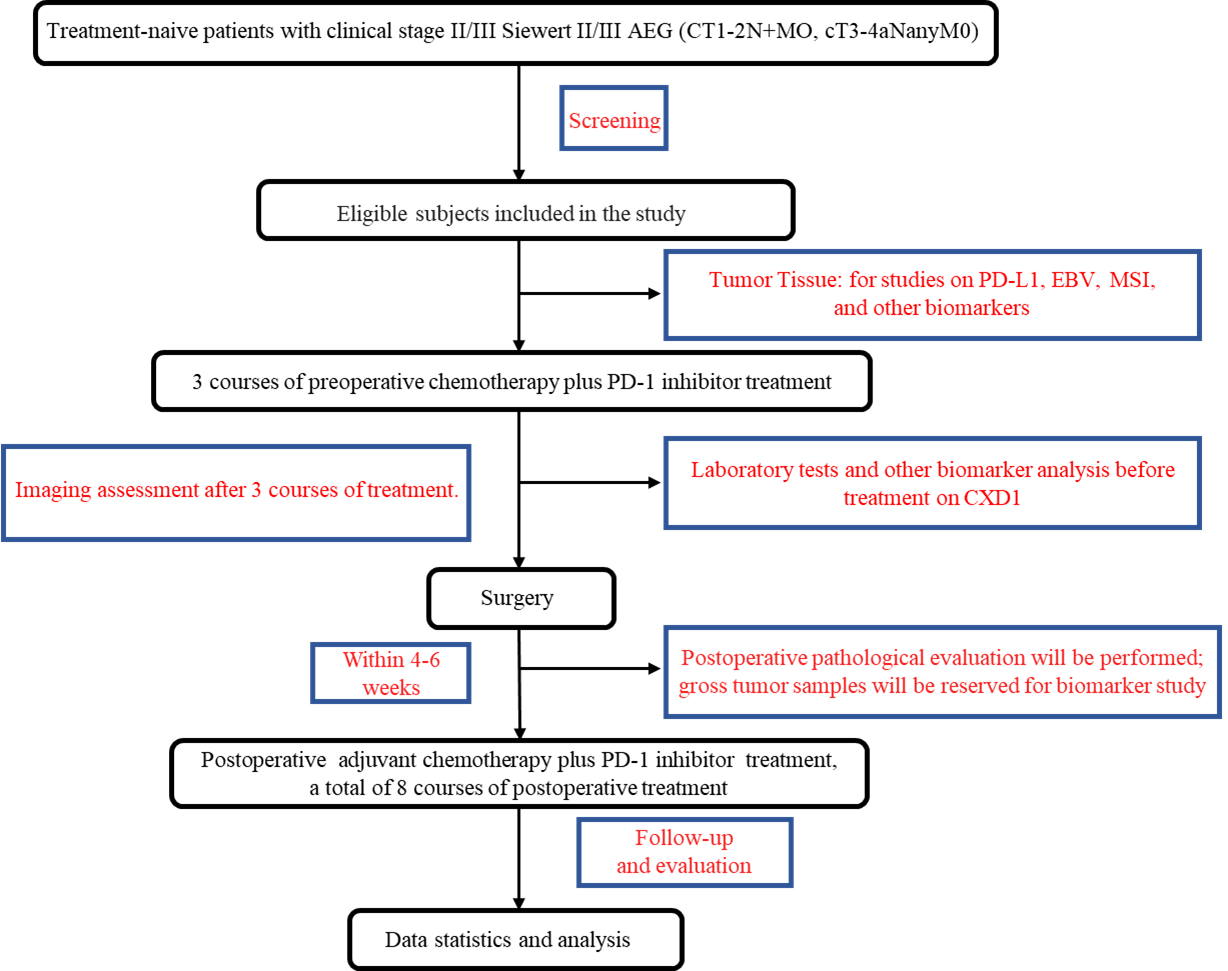


## 5.1 Screening/Baseline Period (Day -28 to Day -1)

- All subjects must provide a signed written ICF prior to any specific study assessments and procedures;
- Demographic data: including date of birth, gender, race/ethnicity;
- History of drinking and smoking;
- Previous medical history (collect all previous medical history that started before signing the ICF, and considered relevant to the study, other than for this indication);
- Previous tumor history: including the diagnosis date, and start/stop date of the previous treatment regimen. Previous significant procedures (such as gastroscopy, puncture biopsy and other diagnostic or therapeutic invasive procedures) should be recorded in the case report form, including start and end dates, name and site of operation;
- Obtaining tumor tissue specimens: All subjects should provide at least 5 tumor tissue sections for PD-L1 detection and 6 tumor tissue sections for EBV and/or MSI detection. A gastroscopic biopsy (if applicable) will be performed to obtain a fresh tissue specimen for subsequent exploratory research.
- Physical examination: including height, weight, head, eyes, ears, nose, throat, neck, heart, chest (including lungs), abdomen, extremities, skin, lymph nodes, nervous system and general condition.
- Vital signs;
- ECOG Performance Status: This evaluation shall be performed by the same investigator during the study. See Appendix 5 for details;
- Laboratory tests: the laboratory tests will be completed in the local laboratory of the study center and should be performed within 7 days prior to the first dose. Specific laboratory test indicators include hematology, blood chemistry and urinalysis (the same as below).
- Hematology: red blood cell count, hemoglobin, hematocrit, white blood cell count and differential [neutrophils, lymphocytes, eosinophils, monocytes, basophils, and other cells] and platelet count;
- Blood chemistry: total protein, albumin, globulin, blood glucose, total cholesterol, low-density lipoprotein, high-density lipoprotein, triglyceride, urea, creatinine, alkaline phosphatase, lactate dehydrogenase, total bilirubin, direct bilirubin, indirect bilirubin, AST, ALT, calcium, phosphorus, magnesium, potassium, sodium, chloride, serum amylase, and uric acid;
- Urinalysis: specific gravity, PH, urine glucose, protein, cast, ketone body, blood cells.
- Serum pregnancy test (females of childbearing age only);
- INR and aPTT;
- Cardiac ultrasonography (Especially the evaluation of left ventricular ejection function);
- 12‑Lead ECG;
- Virology test: 5 markers of Hepatitis B (HBsAg, anti-HBs, HBeAg, anti-HBe, anti-HBc) (HBV DNA copies should be detected in case of positive HBsAg), HCV antibody, HIV antibody;
- Thyroid function: thyroid stimulating hormone (TSH), serum free triiodothyronine (FT3), serum free thyroxine (FT4);
- Tumor assessment: the tumor will be evaluated per RECIST 1.1 criteria. Tumor assessment at screening must be performed within 3 weeks prior to enrollment, and chest, abdomen and pelvic cavity CT or MRI scan should be performed. Patients should be clinically staged according to AJCC/UICC Version 8 TNM staging. For patients with cT3-4aN+M0 based on imaging assessment, it is recommended to perform laparoscopic exploration to exclude liver metastasis and peritoneal metastasis (including positive ascites cytology) if the center is qualified. Imaging examinations conducted for routine evaluation of tumors do not need to be repeated if they are done at the same study site and are within 3 weeks prior to enrollment;
- The inclusion/exclusion criteria will be reviewed to evaluate the subject's eligibility;
- Subject enrollment: after checking the inclusion/exclusion criteria and confirming the eligibility of the subject, the subject information should be activated by logging in to the EDC system within 3 days prior to administration;
- EBV blood test: for patients with positive EBER IHC test during the screening period, EBV DNA test should be performed;
- Peripheral blood ctDNA test;
- Peripheral blood ctcPD-L1 test;
- Concomitant medication/therapy: all drug therapies used within 28 days prior to the enrollment must be recorded in the case report form, including the generic name and daily dose, medication reasons, and the start date and the end date.
- Adverse events (AEs): AEs should be collected from the time of signing the informed consent form until 60 days after the last dose and should be recorded on the AE page of the eCRF.

## 5.2 Preoperative and Postoperative Treatment Period Visits

- Physical examination;
- Vital signs;
- ECOG performance status;
- Laboratory tests: laboratory tests during the treatment period should be completed before each dose, except for C1D1. Blood samples should not be collected earlier than 3 days before each dose. The dose can only be started after the laboratory test results meet the criteria as determined by the investigator;
- INR and aPTT: if clinically indicated;
- Cardiac ultrasound: evaluated before surgery in the screening period and within 7 days before surgery, and every 12 weeks after surgery;
- 12‑Lead ECG;
- Thyroid function: once before dosing in each cycle and once at the end-of-treatment visit. In case of any clinically significant changes in thyroid function, endocrinology consultation is recommended to rule out pituitary function impairments;
- Tumor assessment: the tumor will be evaluated per RECIST 1.1 criteria. Imaging assessment will be performed again after 3 courses of preoperative treatment, every 3 months for the first 2 years, every 6 months for the first 3-5 years, and yearly thereafter for tumor recurrence.
- Peripheral blood ctDNA test;
- Peripheral blood ctcPD-L1 test;
- Subjects with response evaluation as progressive disease (PD) may be encouraged to voluntarily participate in the optional biomarker study if tumor lesion tissue is available and provide tumor tissue for exploratory study on the correlation between tumor markers and anti-tumor response.
- Treatment with investigational drug;
- Concomitant medication/therapy;
- Adverse events;

## 5.3 Operative Period

- Informed consent: written ICFs should be obtained from all subjects before surgery and operation;
- Tumor tissue collection: the subject consents to the investigator to obtain the surgical tissue specimen for the subsequent exploratory study;
- Vital signs and physical examination;
- ECOG performance status;
- Laboratory tests: within 7 days before surgery, as routine preoperative preparation;
- INR and aPTT;
- The evaluation of left ventricular ejection function should be specifically noticed in cardiac ultrasonography;
- 12‑Lead ECG;
- Thyroid function: thyroid stimulating hormone (TSH), serum free triiodothyronine (FT3), serum free thyroxine (FT4);
- Tumor assessment: CT or MRI scan of the chest, abdomen and pelvis will be performed after 3 courses of preoperative treatment to evaluate the tumor according to RECIST v1.1. Laparoscopic exploration should be performed first to determine whether there is peritoneal implantation or liver metastasis. Postoperative pathological evaluation should be performed according to AJCC/UICC 8th edition ypTNM staging and TRG grading (Appendix 6);
- Adverse events: the investigator will evaluate the surgical complications within 30 days after the operation, mainly including hemorrhage of digestive tract, anastomotic leakage, pancreatic fistula and incision complications (including infection, bleeding and dehiscence) according to the Clavien-Dindo Classification of Complications (Appendix 7).

## 5.4 End-of-Treatment Visit

Subjects must have a treatment discontinuation visit within 30 days of the last dose of the study drug or prior to the initiation of a new antineoplastic therapy, whichever occurs first. The visit with tumor progression or loss of clinical benefit according to RECIST 1.1 criteria can be considered a treatment discontinuation visit.

- Physical examination;
- Vital signs;
- ECOG performance status;
- Laboratory tests;
- INR and aPTT;
- Serum pregnancy test (females of childbearing age only);
- Cardiac ultrasound;
- 12‑Lead ECG;
- Thyroid function;
- Tumor evaluation: Baseline and subsequent imaging should be conducted by the same imaging method (CT or MRI) and evaluated by the same reader, if possible;
- Peripheral blood ctDNA test;
- When PD occurs, if there are tumor lesions amenable to biopsy and the subject agrees to participate in the optional biomarker study;
- Concomitant medication/therapy;
- Collection of adverse events/serious adverse events.

## 5.5 Treatments after Tumor Recurrence/Metastasis

The investigational drug may be continued if the subject enrolled in the test group can still benefit from the PD-1 antibody treatment after tumor recurrence/metastasis at the discretion of the investigator, with permission from the investigator and the sponsor, or the medical monitor of the sponsor-authorized CRO, but the original chemotherapy must be terminated.

If a subject in the test group experiences re-progression after continuing investigational drug treatment, he/she should withdraw from the study permanently. The investigator should determine re-progression based on RECIST v 1.1, taking the result of tumor evaluation at the time of the first progression as the baseline value.

Subjects cannot continue to receive the investigational drug after PD unless the following criteria are met:

- The benefit of a continued dose of PD-1 antibody overweighs the risk as judged by the investigator;
- ECOG score ≤ 2.

## 5.6 Survival Follow-up

Adverse events should be recorded until 60 days after the last dose of the investigational drug or until the start of any other anticancer therapy, whichever occurs first. After the period, only serious adverse events considered by the investigator to be related to prior study treatment will be recorded. In addition, if there is an unresolved adverse event or abnormal laboratory test result that is considered related to study treatment, the subject will continue to be followed until the event is resolved or returns to baseline and is stable assessed by the investigator, the subject is lost to follow-up, withdraws consent, or it is determined that study treatment or study participation is not attributed to study treatment or study participation.

For subjects who end the treatment for reasons other than disease progression, tumor recurrence assessments will be performed every 3 months for the first 2 years, every 6 months for the first 3-5 years, and yearly thereafter. The assessments are better performed using the same imaging methods and by the same investigator as much as possible, until disease progression, death, initiation of new antineoplastic therapy, or withdrawal of consent, whichever occurs first.

For subjects with PD, the information on subsequent anti-tumor treatment and survival status will be collected every 3 months from the end of the treatment visit.

1. Study Drugs and Study Method

## 6.1 Study Drug Supply

### 6.1.1 Name: Tislelizumab Injection

Dosage form: sterile solution for injection

Strength:100mg/10ml/ vial

Active Composition：Tislelizumab，a humanized monoclonal antibody directed against PD-1.(IgG4 variant).

Excipient Composition：Sodium citrate dihydrate, citric acid monohydrate, L-histidine hydrochloride monohydrate, L-histidine, alginate dihydrate, polysorbate 20 and water for injection.

Storage condition: Refrigerated at 2-8 ℃, protected from light.

Shelf Life: 24 months

The preparation of the drugs used in the trial complies with Good Manufacturer Practices for Pharmaceutical Products and the quality complies with the quality standard of investigational drugs for clinical trials.

### 6.1.2 Chemotherapeutic drugs

Chemotherapeutic drugs: Oxaliplatin, and tegafur/gimeracil/oteracil are provided by the sponsor. Please refer to the package inserts of each drug for the strength and storage conditions.

## 6.2 Management of Investigational Drug

### 6.2.1 Receipt and Storage

The sponsor will provide the study drugs according to the expected enrollment plan of the study site. The study drugs will be shipped to the study site via a third-party logistics company qualified for shipment. Upon receipt of the study drugs, the designated recipient at the site will check the shipment status, confirm the number and status of vials, complete inventory and drug accountability records, and finally fax the signed shipment note to the sponsor or an authorized third party to confirm drug receipt.

The study drugs will only be used for this study and be managed by a person authorized by the investigator. To fully control the dispensing and use of the study drugs, storage will be registered at each subject dosing visit.

### 6.2.2 Disposition

All unused Tislelizumab Injections will be stored under specified storage conditions (refrigerated at 2-8 ℃, protected from light) in the designated storage place by the clinical pharmacology facility at the study site. The sponsor will regularly arrange for third-party personnel to recover the used residual solution and empty vials of Tislelizumab Injection at the study site. If medication is lost or damaged, the occurrence should be documented in detail. At the end of the study, the remaining unused study drugs and the used remaining liquid and empty vials will be returned to the sponsor or a designated third party.

Chemotherapeutic drugs are provided by the sponsor and stored according to the package insert and the procedures of the study site.

### 6.2.3 Preparation Method and Record

#### 6.2.3.1 Tirilizumab Preparation Method and Record

The product is a clarified to slightly opalescent, colorless to light yellow liquid. Careful examination should be performed before use to confirm that each vial of Tirilizumab Injection is not damaged and that the solution in the vial does no appear coagulation, turbidity, precipitation, particles, color anomalies before use.

Tirilizumab is administered at a fixed dose of 200 mg Q3W.

It will be diluted with normal saline (0.9% sodium chloride solution) to a final concentration of 1-5 mg/mL under aseptic conditions. It is recommended to withdraw the corresponding volume of the investigational drug with a syringe and inject 100 mL of normal saline. Gently invert the diluted solution 3-5 times to mix well, avoid forceful shaking, and use an in-line filter (0.2 or 0.22 um) for intravenous drip after mixing for at least 60 minutes. As the product does not contain antimicrobial preservatives, the solution preparation must be carried out under aseptic conditions.

If the prepared diluted solution cannot be used immediately, it may be stored in a refrigerator at 2 ℃ to 8 ℃ for up to 16 hours or at room temperature for up to 4 hours.

**At the end of drip infusion for subjects, it is necessary to flush the tube with 100 mL of normal saline**. The used drug vials and packaging must be handed over to the pharmacist for recovery.

Accurate records of the volume of the investigational drug infused by the subject during the study are required. Each vial of Tirilizumab should only be used by one subject and is not interchangeable. If there is remaining fluid after the dose of Tirilizumab, it should not be used again. The remaining drug needs to be sealed and returned to the pharmacist for subsequent recovery.

#### 6.2.3.2 Preparation Method and Record of Combination Chemotherapy

The chemotherapy treatment will start 1 hour after the end of the infusion of Tirilizumab (see Section 6.3) and after close monitoring of vital signs.

Chemotherapy drugs are prepared according to the package insert used in each center, and the dose is referred to as follows.

- **SOX regimen:**

Oxaliplatin: 130 mg/m^2^, iv drip over 3 hours on d1, once every 3 weeks;

Tegafur/gimeracil/oteracil: initial dose is 40 mg/m^2^/dose (40 mg/dose for body surface area < 1.25 m^2^; 50 mg/dose for body surface area of 1.25 to 1.5 m^2^; 60 mg/dose for body surface area ≥ 1.5 m^2^). It will be administered bid, p.o on d1~14, once every 3 weeks (refer to the package insert).

Each cycle consists of 21 days. The patients will be treated with SOX chemotherapy for 3 courses, and then receive routine surgery. After 4-6 weeks, the patients will receive SOX chemotherapy for 5 courses, until disease recurrence, any unacceptable toxicity, withdrawal of consent, or treatment discontinuation.

Tegafur/gimeracil/oteracil will be considered not administered if a patient vomits within 30 minutes after dosing, and it will be recorded in the patient diary card and eCRF. Patients who experience vomiting must not receive replacement therapy. Tegafur/gimeracil/oteracil should not be administered more frequently than twice daily in any case.

## 6.3 Medication Considerations

1. Prophylactic medication

PD-1 inhibitors should not be administered via intravenous bolus injection or rapid injection and peripheral or central venous access should be established. Prior to infusion, an adequate amount of epinephrine, diphenhydramine hydrochloride for intravenous injection or other anti-allergic drugs, and resuscitation equipment should be available in case of severe anaphylactic reactions. After infusion, the venous access should be left open for administration (if needed). If there are no complications, the infusion of chemotherapeutic drugs can be performed only after 1 hour of observation after the end of the infusion. See Table 1.2 below for details.

Avoid exposure to cold air, and cold water during Oxaliplatin treatment (3-5 days after infusion).

It is recommended that antiemetics, such as 5-HT3 antagonists, dexamethasone or NK1 antagonists, be used prior to the initiation of intravenous chemotherapy as per site practice to prevent vomiting.

1. Monitoring during the intravenous drip

The first dosing of Tirilizumab should be performed under the supervision of an experienced physician. Before, during, and at least 1 hour after the infusion, the vital signs should be closely monitored (body temperature, respiration, blood pressure, and heart rate are monitored using an ECG monitor), and the subject's complexion, sweating, or headache must also be closely observed, so as to early detect signs of infusion reactions. See Table 1.2 below for details.

Some infusion reactions may occur during subsequent administration, even if no infusion reaction of any grade occurs after the first administration, the subsequent administration process should still be carried out under the supervision of a physician.

1. Management of infusion reactions

If the subject has ever experienced an infusion reaction, prophylactic anti-allergic medications should be administered prior to each subsequent dose of the investigational drug. Despite the use of antihistamines and corticosteroids to prevent allergic reactions, infusion reactions may still occur when the antibody is infused. For specific treatment, please refer to 8.1.3.1 Infusion Reaction.

**Table 1.2 First and Subsequent Tirilizumab Infusions**

| First infusion | Subsequent infusions |
| --- | --- |
| Premedication is not allowed | If the subject has experienced an infusion reaction during a previous infusion, appropriate pre-treatment for subsequent infusions should be administered under the guidance of the investigator. |
| The subject's vital signs (heart rate, respiratory rate, blood pressure, and temperature) should be recorded within 60 min prior to infusion | The subject's vital signs should be recorded within 60 minutes prior to infusion |
| PD-1 antibody is infused over at least 60 minutes | If the first infusion is tolerated and there are no infusion-related adverse events, the PD-1 antibody infusion can be administered over 30 (± 10) minutes; if an infusion reaction occurs in the prior infusion, the infusion time for PD-1 inhibitors should be at least 60 minutes. |
| If clinically indicated, vital signs must be closely monitored at 15, 30, 45, and 60 minutes (± 5 minutes) during infusion, and within 30 minutes (± 5 minutes) after infusion | If infusion reactions or clinical indications occur in the prior infusion, the patient's vital signs must also be monitored for 30 min (±5 min) after infusion. |
| Subjects will be informed of possible delayed post-infusion symptoms and asked to contact their study doctor if they experience these symptoms |  |

## 6.4 Concomitant Medications and Concurrent Treatments

Any medications taken by the subject must be approved by the investigator and used under the direction of the investigator.

Symptomatic treatments given due to adverse events should be recorded. If necessary, subjects can be given adequate supportive treatment, including transfusion of whole blood and blood products, antibiotic treatment, anti-allergic treatment, anti-diarrhea treatment, etc. The date, reason, and dose of the treatment should be recorded in detail.

During the study, the subjects are allowed to receive comprehensive supportive treatment, as well as antiemetics or diuretics related to chemotherapy drugs. Other anti-tumor-related radiotherapy, chemotherapy, immunotherapy, hormone therapy (except drugs for anti-allergic reactions), Chinese patent medicines with anti-tumor activity specified in the package insert, surgery, or other clinical trial drugs are not allowed. If the investigator judges that the subject requires any other specific anticancer therapy, the subject should discontinue the investigational drug treatment prematurely before receiving a new anticancer therapy.

Subjects may use topical, ocular, intra-articular, intranasal, and inhaled corticosteroids (with minimal systemic absorption). A brief course of corticosteroids for prevention (e.g., contrast dye allergy) or treatment of non-autoimmune conditions (e.g., delayed-type allergic reactions caused by contact allergens) or management of adverse events caused by the investigational drug is allowed.

## 6.5 Chemotherapy Dose Modifications

The general principles for dose adjustment in the protocol are as follows:

- In the case of toxicity due to only one drug of the study treatments judged by the investigator, this drug treatment should be delayed or modified according to the guidelines below, and the other drugs should be continued if there are no contraindications.
- Any patient who requires a dose reduction will continue to receive the reduced dose administration in subsequent treatment cycles. For any patient who has had 2 dose reductions, the study treatment must be discontinued if the third dose reduction is required due to toxicity. Treatment may be delayed until 21 days after the first day of this treatment cycle to allow sufficient time for the patient to recover from study drug-induced toxicity.
- If concomitant conditions are existing at baseline, the dose will be adjusted according to the corresponding change in toxicity grade as deemed appropriate by the investigator. For example, if Grade 1 asthenia occurs in a subject at baseline, and increases to Grade 2 during treatment, a Grade 1 change occurs and is considered as Grade 1 toxicity for dose adjustment.
- For severe toxicities with different grades that occur at the same time, dose adjustments should be made according to the highest grade toxicity observed.
- In the case of toxicity due to only one chemotherapeutic drug judged by the investigator, no dose adjustment of the other chemotherapeutic drug is required.
- For patients who temporarily discontinue chemotherapy due to adverse reactions, Tirilizumab treatment may be continued if the Tirilizumab treatment requirements are met according to the original plan.
- Subjects who discontinue chemotherapy due to toxicity should not withdraw from all study treatments but continue to complete the treatment with Tirilizumab.
- In addition to the chemotherapy dose adjustment recommended in this section, dose adjustment may also be made according to local product information and standard clinical practice.
- See Section 8.2 for specific chemotherapy toxicity management and dose modifications

## 6.6 Overdose

An overdose is defined as a subject receiving more than 20% of the planned dose for any reason.

Adverse events caused by overdose should be judged as SAE and reported and treated symptomatically according to the provisions in Section 8.3 Serious Adverse Events.

1. Biomarker Tests

In addition to routine testing of PD-L1 expression in tumor tissue during the screening period, this study plans but is not limited to the test of the following biomarkers, and a retrospective analysis will be performed:

- Screening period: expression of EBV virus DNA in tumor tissues, EBER FISH test (each subject should provide at least 3 unstained sections of primary or metastatic tumor tissues);
- Screening and treatment periods: for patients with positive EBER FISH test at screening, EBV DNA test will be performed at screening, 7 days before the surgery, 6 months, and 12 months after the surgery, respectively;
- Screening and treatment periods: peripheral blood ctDNA test;
- Screening period: MSI (PCR) expression in the subject's tumor tissue (each subject should provide at least 3 unstained sections of primary or metastatic tumor tissues);
- Screening period: state of lymphocyte infiltration in tumor tissue;
- Operative period: The subject consents to the investigator to obtain the surgical tissue specimen for the subsequent exploratory study.

Subjects with response evaluation as PD may be encouraged to voluntarily participate in the optional biomarker study (Sub Study, Paired Biopsy) if tumor lesion tissue is available. In the biomarker study, endoscopic sampling of tumor tissue will be performed to observe tumor tissue response to the investigational drug. Each subject is required to provide at least 5 unstained sections of primary or metastatic tumor tissues. Subjects who participate in the sub-study are required to sign a separate sub-study informed consent form before relevant endoscopic procedures can be performed. This optional informed consent for biomarker research may be signed at any time during the study. In addition, the subject may withdraw at any time.

Tumor tissue and/or hematology specimens collected for this study and the optional biomarker study are limited to exploratory research purposes for this study and the optional biomarker study. Approval must be obtained from the site's Ethics Committee prior to tumor tissue and blood specimen collection for PD-L1 and exploratory purposes at the site.

1. Safety Assessments

The safety profile of perioperative chemotherapy plus Tirilizumab in locally advanced gastric cancer is unclear. The following information regarding Tirilizumab is based on the nonclinical and clinical studies, as well as published data on similar molecules. It is the responsibility of the investigator or study site personnel to monitor, record, and report events that meet the definition of an AE or SAE.

## 8.1 Overall Plan to Manage Tirilizumab Safety Issues

Tirilizumab will be administered in the presence of emergency medical facilities and staff trained in emergency monitoring and handling. During the study, all adverse events and serious adverse events will be recorded until 60 days after the last dose of the study drug or initiation of new anticancer therapy, whichever occurs first. After this time point, investigators are asked to report all serious adverse events related to the study treatment, regardless of whether they occurred after the end of the study.

### 8.1.1 Monitoring

In this study, safety will be assessed by monitoring all serious adverse events and adverse events (defined and graded according to NCI CTCAE version 5.0). Subjects will be evaluated for safety (including laboratory test results) according to the SOA in Appendix 4. Laboratory test results must be reviewed prior to each investigational drug infusion.

General safety assessments will include: continuous medical history, physical examinations, and specific laboratory tests including serum chemistry and blood counts (see Appendix 4 for a list and timing of study assessments).

Subjects will be closely monitored for any signs and symptoms of autoimmune disease and infection during the study.

All serious adverse events are reported in Section 8.6.2.

During the study, all adverse events and serious adverse events will be recorded until 60 days after the last dose of the study drug or initiation of new anticancer therapy, whichever occurs first. Thereafter, the investigator will be asked to report all serious adverse events related to the study treatment, regardless of whether they occur after the end of study.

In addition, subjects with ongoing unresolved study drug-related adverse events (including abnormal laboratory test results) after the end of the study or at the time of study treatment discontinuation will be followed until the event is resolved or returns to baseline and is stable assessed by the investigator, the subject is lost to follow-up, withdraws consent, or it is determined that adverse event is not attributed to study treatment or study participation.

### 8.1.2 Tirilizumab Dose Modification

No dose reduction of Tirilizumab is required for the test group in this study. If a subject experiences an adverse event that requires dose interruption, the subject may withhold study treatment and Tirilizumab may be temporarily discontinued after discussion with the investigator to decide whether the chemotherapy regimen is still being administered as planned. If the subject discontinues Tirilizumab for more than 56 days due to an adverse event and the investigator judges that the risks of continuing Tirilizumab treatment outweigh the benefits, permanent withdrawal from study treatment will be considered and safety follow-up will be performed as described in Section 8.1**.**

After permanent discontinuation of Tirilizumab due to adverse reactions, the original chemotherapy regimen can be continued until progression, intolerance, investigator's judgment, or voluntary withdrawal for a maximum of 8 cycles of chemotherapy if the patient can still obtain benefits judged by the investigator.

If the patient is likely to continue to gain clinical benefit from Tirilizumab after discontinuation judged by the investigator, the investigational drug treatment may be resumed after approval by the medical monitor**.**

If a subject needs to taper steroids due to an adverse event, Tirilizumab may be discontinued for a long time until the steroid taper is completed or to a prednisone dose ≤ 10 mg/day (or equivalent). If discontinued for more than 56 days, the investigational drug may be restarted with the approval of the medical monitor based on an overall risk-benefit assessment by the investigator in consultation with the medical monitor**.**

Dosing may be interrupted for reasons other than toxicity (e.g., surgery) with the approval of the medical monitor. The investigator, in consultation with the medical monitor, may resume the investigational drug after approval by the medical monitor based on an overall risk-benefit assessment**.**

The investigator must discuss any decision to permanently discontinue the investigational drug in advance with the investigator and document the outcome of the discussion**.**

Please refer to the Tirilizumab Investigator's Brochure for further details on dose adjustment for Tirilizumab**.**

### 8.1.3 Management of Special Adverse Events

#### 8.1.3.1 Infusion-related adverse reactions

Clinical symptoms of infusion reactions include pyrexia, chills, nausea, pruritus, vasogenic edema, hypotension, headache, tracheospasm, urticaria, rash, vomiting, muscle pains, and somnolence or hypertension. Possible serious reactions include acute respiratory distress syndrome (ARDS), myocardial infarction, ventricular fibrillation and cardiogenic shock. Therefore, the subjects in this study must be closely observed for relevant clinical symptoms. See Appendix 8**.**

ECG monitoring and rescue medication (including but not limited to epinephrine, glucocorticoids, antihistamines, bronchodilators, and oxygen) are required for rescue if a serious reaction occurs. In the event of infusion-related adverse reactions or suspected events of CTCAE Grade 2 or above, the infusion of Tirilizumab must be stopped immediately (see Table 2 for details). During the first intravenous infusion of Tirilizumab, vital signs should also be monitored within 30 minutes (± 5 minutes) after the end of infusion. The subjects will be informed about the possibility of delayed infusion-related adverse reactions and instructed to contact the investigator if developing the symptoms**.**

The subjects must be treated according to local best practices if a serious allergic reaction occurs**.**

**Table 2 Treatment Modification Guidelines for Infusion Adverse Reactions**

| **CTCAE Grade** | **Modification measures** |
| --- | --- |
| Grade 1 - minor | Transient minor reactions, infusion interruption and clinical intervention are not recommended. Slow the infusion rate by 50% and observe closely for any worsening symptoms. Clinical intervention is provided as necessary. |
| Grade 2 - moderate | Withhold PD-1 antibody therapy and immediately administer systemic therapy (e.g., antihistamines, NSAIDs, narcotics, IV fluids); When infusion reaction resolves to Grade 0-1, resume dosing and reduce infusion rate by 50%. Observe closely for any worsening symptoms. Take appropriate therapeutic interventions according to local medical practices. |
| Grade 3 - severe | Immediately stop the infusion and remove the infusion line. The investigator will discuss whether to resume the investigational drug with the investigator according to the actual situation of the subject.  If the treatment is resumed, the infusion should last for at least 2 hours for the subsequent treatment and relevant prophylactic medications (e.g., diphenhydramine and NSAID drugs) should be administered. At the same time, the clinical symptoms of relevant infusion reactions should be closely observed.  Take appropriate therapeutic interventions according to local medical practices |
| Grade 4 - life-threatening; urgent clinical intervention indicated | Subjects who experience Grade 4 infusion reactions must immediately and permanently discontinue treatment and be withdrawn from the study. Take appropriate therapeutic interventions according to local medical practices. |

If an IRR of CTCAE grade 2 or higher occurs again, the subjects must immediately discontinue the drug permanently and withdraw from the study**.**

#### 8.1.3.2 Serious Allergic Reactions

Antibody administration may cause allergic reactions. Appropriate drugs and medical equipment must be provided immediately to treat acute allergic reactions, and the study personnel must be trained to identify and treat allergic reactions. The study site must be equipped with first-aid team and equipment, and can admit the subject into an intensive care unit if necessary. The subject must be given emergent treatment according to local medical practices when a serious allergic reaction occurs. Subjects must be given epinephrine and dexamethasone immediately as well as ECG monitoring. Blood collection could be considered for serum IgE testing. Subjects must immediately discontinue drugs permanently and withdraw from the study treatment. When a subject experiences such symptoms, he/she must inform the investigator immediately**.**

**Allergic reaction**

In the guidelines of National Institute of Allergy and Infectious Diseases (NIAID) and Food Allergy & Anaphylaxis Network (FAAN), anaphylaxis is defined as severe allergic reactions with rapid onset and possible death (Sampson et al 2006). These three types of allergic reactions, as shown below, cover 80% (category 1) to 95% of the cases (all three categories)

1. Acute (minutes to hours) allergic reaction involving skin, mucosal tissue, or both (e.g., generalized urticaria, pruritus or flushing, swollen lips and tongue)

and at least one of the following:

1. Dyspnea (e.g., dyspnea, stridor-bronchospasm, stridor, peak expiratory flow (PEF) decreased, hypoxemia).
2. Reduced blood pressure (BP) or associated symptoms of end-organ dysfunction (e.g., hypotensive shock, syncope, incontinence).
3. The subject may experience two or more of the following after exposure to an allergen (minutes to hours):
4. Involvement of the skin-mucosal tissue (e.g., generalized hives, pruritus, swollen lips and tongue).
5. Dyspnea (e.g., dyspnea, stridor-bronchospasm, stridor, PEF decreased, hypoxemia).
6. Reduced blood pressure or associated symptoms (eg, hypotensive shock, syncope, incontinence).
7. Persistent gastrointestinal symptoms (e.g., abdominal pain, vomiting).
8. Blood pressure decreases in patients exposed to known allergens (minutes to hours):
9. Infants and children: low systolic blood pressure (age-specific) or less than 30% reduction in systolic blood pressure
10. Adults: Systolic blood pressure less than 90 mm Hg or more than 30% lower than the baselin.

#### 8.1.3.3 Immune-related adverse events (irAEs)

Tirilizumab may be associated with the following potentially significant immune-related adverse events: immune-related hepatitis, pneumonitis, colitis, pancreatitis, and endocrine disorders (hypothyroidism, hyperthyroidism, adrenal cortex insufficiency). Refer to the Investigator's Brochure for a detailed description of anticipated safety risk management for Tirilizumab.

In addition, immune-related adverse reactions also include the following clinically significant events: exfoliative dermatitis, uveitis, arthritis, myocarditis, pancreatitis, hemolytic anemia, partial seizures (which may occur in subjects with inflammation of the cerebral hemispheres), adrenal insufficiency, myasthenia gravis, optic neuritis, and rhabdomyolysis.

Suspected immune-related adverse reactions should be closely observed for relevant system functions, and an adequate evaluation should be warranted to determine the etiology and rule out other causes. Overall, Tirilizumab should be suspended or permanently discontinued and/or symptomatic treatment, such as corticosteroids, should be administered depending on the severity of the event. When prednisone 1-2 mg/kg or equivalent corticosteroid is administered, the dose should be gradually tapered after the event resolves to Grade 0-1. Refer to the Investigator's Brochure for a detailed description of risk management. At this time, if irAEs are still Grade 0-1, resume Tirilizumab treatment. The occurrence of Grade 3 or higher irAE (except endocrine disorders) required immediate permanent discontinuation of the investigational drug and withdrawal from the study.

## 8.2 Chemotherapy Safety Management and Dose Modification

General principles for dose adjustment of chemotherapy agents are described in Section 6.5. In addition to the chemotherapy dose adjustment recommended in this section, toxicity management and dose adjustment may also be made according to local product information and standard clinical practice.

### 8.2.1 Dose Modification of SOX Regimen

Dose adjustment for hematologic toxicity

If the subject's absolute neutrophil count (ANC) is ≥ 1.5 × 10^9^/L and platelet count (PLT) is ≥ 100 × 10^9^/L at the start of a course, a new 3-week course can be started. Otherwise, treatment needs to be delayed until hematology parameters have recovered. At the start of subsequent treatment cycles, dose adjustments should be made based on the nadir hematology count in the prior treatment cycle. Treatment may be delayed for up to 3 weeks if the relevant tests are not satisfactory to allow sufficient recovery time. Detailed guidance for dose adjustment based on hematologic toxicity after patient recovery is provided in Table 3.

Table 3 Dose Modification Regimen for Tegafur/Gimeracil/Oteracil (S) plus Oxaliplatin (O) Based on Hematologic Toxicities During Planned Treatment

| Toxicity ^a^ | Dose modifications for tegafur/gimeracil/oteracil and oxaliplatin at the restart of treatment |
| --- | --- |
| ANC ≥ 1.0 and < 1.5 (× 10^9^/L)  and/or  PLT ≥ 50 and < 75 (× 10^9^/L) | First occurrence: S: 100% of dose, O: 130 mg/m^2^  Second occurrence: S: 75% of dose, O: 100 mg/m^2^  Third occurrence: S: 50% of dose, O: 65 mg/m^2^  Fourth occurrence: permanently discontinued |
| ANC ≥ 0.5 and < 1.0 (× 10^9^/L)  and/or  PLT ≥ 25 and < 50 (× 10^9^/L) | First occurrence: S: 75% of dose, O: 100 mg/m^2^  Second occurrence: S: 50% of dose, O: 65 mg/m^2^  Third occurrence: permanently discontinued |
| ANC < 0.5 (×10^9^/L)  and/or  PLT < 25 (×10^9^/L) | First occurrence: S: 50% of dose, O: 65 mg/m^2^  Second occurrence: permanently discontinued |
| Grade 3 febrile neutropenia (ANC < 1.0 (×10^9^/L) with pyrexia ≥ 38.5°C | First occurrence: S: 50% of dose, O: 65 mg/m^2^  Second occurrence: permanently discontinued |
| Grade 4 febrile neutropenia (ANC < 1.0 (×10^9^/L) with fever ≥38.5°C, associated with life-threatening sepsis | Permanently discontinued. |

^a^ The lowest value during the prior treatment courses. S = Tegafur/Gimeracil/Oteracil, O = Oxaliplatin

If hematologic toxicity is found in unscheduled assessments during the treatment, the dose of tegafur/gimeracil/oteracil must be interrupted for this course, and the dose of tegafur should be reduced in subsequent courses, as shown in Table 4.

Table 4 Dose Modifications for Hematologic Toxicity during Treatment Cycles of Tegafur/Gimeracil/Oteracil (X)

| Toxicity ^a^ | Dose Modifications of Tegafur/Gimeracil/Oteracil during the 1-14 Day Treatment Period |
| --- | --- |
| ANC ≥ 1.0 and < 1.5 (× 10^9^/L)  and/or  PLT ≥ 50 and < 75 (× 10^9^/L) | Withhold treatment until recovery to Grade 0-1. Take the original dose during the treatment course, and do not make up for the missed dose. |
| ANC ≥ 0.5 and < 1.0 (× 10^9^/L)  and/or  PLT ≥ 25 and < 50 (× 10^9^/L) | Withhold treatment until recovery to Grade 0-1. Then take 75% of the original dose during the treatment course, and do not make up for the missed dose. |
| ANC < 0.5 (× 10^9^/L)  and/or  PLT < 25 (× 10^9^/L) | Discontinue treatment until recovery to Grade 0-1 as considered by the investigator. It is in the best interest of the patient to take 50% of the original dose during the treatment course |

^a^ The lowest value during the prior treatment courses. S = Tegafur/Gimeracil/Oteracil, O = Oxaliplatin

Dose Modification for Non-Hematologic Toxicity: Tegafur/Gimeracil/Oteracil

Recommendations for dose modifications of tegafur/gimeracil/oteracil apply to toxicities related to capecitabine but not those related to combination therapy. In case of Grade 1 non-hematologic toxicity, treatment may be continued at the original dose. In case of Grade 2, 3, or 4 non-hematologic toxicity, tegafur/gimeracil/oteracil treatment must be immediately interrupted or discontinued. This dose interruption should be counted as a loss of treatment time, and the missed dose should not be taken. Treatment may be delayed for up to 3 weeks in case of unsatisfactory tests to allow sufficient recovery time, as follows:

In case of Grade 2 non-hematological adverse reactions, the initial dose of the next cycle after the first, second and third occurrence is 100%, 75% and 50% of the original dose, respectively. Furthermore, permanent discontinuation is required for the fourth occurrence.

In case of Grade 3 non-hematological adverse reactions, the initial dose of the next cycle after the first and second occurrence is 75% and 50% of the original dose, respectively. Furthermore, permanent discontinuation is required for the third occurrence.

In case of Grade 4 non-hematological adverse reactions, the initial dose of the next cycle after the first occurrence is 50% of the original dose. Furthermore, permanent discontinuation is required for the second occurrence.

The dose modifications for non-hematologic and neurologic toxicities of oxaliplatin

Recommendations for dose modifications of oxaliplatin apply to toxicities related to oxaliplatin but not those related to combination therapy. Dose modifications of oxaliplatin for non-hematologic toxicities are shown in Table 5, and neurotoxicity is shown in Table 6.

Table 5 Dose Modifications of Oxaliplatin for Non-Hematologic Toxicity

| Toxicity | Grade | Dose modification |
| --- | --- | --- |
| Allergic reaction | 3 or 4 | Permanently discontinue treatment |
| Respiratory symptoms suggestive of pulmonary fibrosis | Any | Interrupt treatment and investigate the cause |
| New onset of interstitial lung disease | Any | Permanently discontinue treatment |
| Onset of nausea and vomiting  after preventive use of antiemetic drugs | 3 | 100 mg/m^2^ |
| Nausea and/or vomiting | 4 | 100 mg/m^2^ |
| Diarrhea | 3 or 4 | 100 mg/m^2^ |
| Stomatitis | 3 | No dose modification required |
| Stomatitis | 4 | 100 mg/m^2^ |
| Skin toxicity | 3 or 4 | Extend the rest time of chemotherapy until recovery to Grade 0-1, with no dose modification required |

Table 6 Dose Modifications of Oxaliplatin for Neurotoxicity

| Toxicity | Grade | Duration | | |
| --- | --- | --- | --- | --- |
|  |  | 1 - 7 days | > 7 days | Continuing during the cycle interval ^a^ |
| Paraesthesia skin/dysaesthesia^b^ not affecting the function | 1 | Dose unchanged | Dose unchanged | Dose unchanged |
| Paraesthesia skin/dysaesthesia affecting the function but not activities of daily living (ADL) | 2 | Dose unchanged | Dose unchanged | 100 mg/m^2^ |
| Paraesthesia skin/dysaesthesia with pain, affecting the function and ADL | 3 | Dose unchanged | 100 mg/m^2^ | Permanently discontinue treatment |
| Paraesthesia skin/dysaesthesia,  disabling or  life-threatening | 4 | Permanently discontinue treatment | Permanently discontinue treatment | Permanently discontinue treatment |
| Acute (during or within 2 hours of the end of infusion) laryngeal dysaesthesia ^b^ |  | Next infusion prolonged to 6 hours ^c^ | NA | NA |
| a Not recovered before the next cycle  b May be caused by coldness  c May be pre-treated with benzodiazepines | | | | |

## 8.3 Safety Parameters and Definitions

Safety assessments include monitoring and recording of AEs, including SAEs, protocol-specified laboratory safety assessments, protocol-specified vital sign measurements, and other protocol-specified measurements critical to the safety assessment.

### 8.3.1 Definition of Adverse Events (AEs)

According to the ICH GCP guidelines, an adverse event is any untoward medical occurrence in a subject administered a pharmaceutical product and does not necessarily have a causal relationship with this treatment. Therefore, an AE may be any of the following:

- An AE can be any unfavorable and unintended sign (including an abnormal laboratory finding), symptom, or disease (new or aggravated) that occurs after the administration of a therapeutic product
- Any new disease or pre-existing condition exacerbated (worsening of the manifestation, frequency, or severity of the pre-existing condition); except as described in Section 8.7.10
- Recurrence of an intermittent disease condition (e.g., headache) not manifested at baseline
- Any deterioration in a laboratory finding or other clinical test (e.g., ECG and X-ray) that is associated with symptoms or leads to a change in study treatment or concomitant medication, or leads to discontinuation of the study drug
- Adverse events related to protocol-specified interventions, including AEs that occur prior to assignment to a treatment arm (e.g., biopsy, an invasive procedure during screening)
- Signs, symptoms, or clinical sequelae of a suspected overdose of either a study drug or a concomitant medication (overdose per se is not to be reported as AEs/SAEs);

Events that fail to meet the definition of AEs include:

- Medical or surgical procedures (e.g., endoscopy, and appendicectomy); any untoward medical events leading to these procedures (e.g., appendicitis, stomach ache, and hemorrhage of digestive tract) should be recorded as AEs;
- Hospitalization with no untoward medical event occurred (hospitalization due to social insurance and/or convenience);
- Anticipated daytime fluctuations of a pre-existing disease(s) or condition(s) present or detected at the beginning of the study that does not worsen;

### 8.3.2 Laboratory Abnormalities

The investigator should consider the following guidelines when deciding whether a change in a laboratory value is an AE**:**

- Laboratory abnormalities leading to a change in the investigational drug (e.g., dose interruption or permanent discontinuation);
- Need for concomitant medications and/or surgical intervention to mitigate laboratory abnormalities;
- Laboratory abnormalities are related to clinical symptoms;
- When the investigator considers a laboratory abnormality to be clinically significant.

### 8.3.3 Serious Adverse Events (SAEs)

A serious adverse event is any untoward medical occurrence that meets any one or more of the following criteria:

- Results in death;
- Life-threatening;
- Note: the term "life-threatening" refers to an event in which the subject was at risk of death at the time of the event, it does not refer to an event that might have caused death if it were more severe.
- Requires inpatient hospitalization or prolongation of existing hospitalization;
- Note: In general, hospitalization signifies that the patient has been admitted (usually involving at least an overnight stay) at the hospital or emergency ward for observation and/or treatment that would not have been appropriate in the physician's office or outpatient setting. An untoward medical event that results in hospitalization and prolonged hospitalization is considered a serious adverse event (SAE). If a complication prolongs hospitalization or fulfills any other serious criteria, the event is serious. When in doubt as to whether "hospitalization" occurred or was necessary, the AE should be considered serious.
- Exceptions not considered AEs are specified in Section 8.7.11.
- Results in disability/incapacity;
- Note: The term "disability" refers to a substantial disruption of a person's ability to conduct normal life functions. This definition does not include events of relatively minor clinical significance, such as slight headaches, nausea, vomiting, diarrhea, influenza, and accidental trauma (such as ankle sprain). These events may affect daily function but do not cause significant loss of function.
- Congenital anomaly/birth defects in the neonate/infant after maternal exposure to the study drug;
- A medically significant event in the opinion of the investigator, e.g., an important medical event that may not be immediately life-threatening or result in death or hospitalization but, based upon appropriate medical and scientific judgment, may jeopardize the patient or may require medical or surgical intervention to prevent one of the other outcomes listed in the definition above. These AEs should also be considered serious, e.g., allergic bronchospasm requiring intensive treatment in an emergency room or at home, or convulsions that do not result in hospitalization.
- Protocol-specified SAEs:
  - Potential drug-induced liver injury, including ALT or AST increased with bilirubin increased or clinical jaundice, as defined by Hy's Law (see Section 8.7.7 for details).
  - Adverse events due to overdose, regardless of the CTCAE grade.

Events that are consistent with the expected pattern of progression of the underlying disease should not be recorded as AEs (e.g., dyspnoea associated with disease progression or general physical health deterioration). These data will be collected as efficacy analysis data only. Events that are uncertain whether they are attributable to disease progression should be reported as AEs

The term "serious" is not synonymous with "severe". Severity is defined as the intensity of an AE (graded as mild, moderate, or severe or judged per the National Cancer Institute-Common Terminology Criteria for Adverse Events [NCI CTCAE version 45.0]; see Section 8.5); the medical significance of the event itself may be relatively small (e.g. severe headache without further findings).

For each AE recorded in the eCRF, its severity and seriousness must be evaluated separately.

## 8.4 Causality Assessment

The investigator should determine whether the AE is reasonably related to the study drug, based on his/her knowledge of the subject, the patient's condition before and after the event, and the evaluation of any potential candidate causes.

The correlation between AEs and the investigational drug will be determined by the investigator based on his/her clinical judgment and the following definitions into five levels: related, probably related, possibly related, unlikely related, and unrelated. The first four items are "yes" for the correlation with the drug. The specific judgment criteria are as follows:

Table 7 Correlation between AE and Study Drug

| Unrelated | The study drug is not used, or there is no correlation between the study medication and the time of occurrence of AEs, or the AE has another definite cause. |
| --- | --- |
| Unlikely related | Evidence of the study medication is available. The occurrence of the AE may be better explained with other reasons. Dechallenge is negative or ambiguous. |
| Possibly related | Evidence of the study medication is available. There is a plausible temporal relationship between the occurrence of AE and the use of the study drug. The AE could be explained with other causes. Positive dechallenge. |
| Probably related | Evidence of the study medication is available. There is a plausible temporal relationship between the occurrence of AE and the use of the study drug. The AE is more plausibly explained with the study drug. Positive dechallenge. |
| Definitely related | Evidence of the study medication is available. There is a plausible temporal relationship between the occurrence of AE and the use of the study drug. The AE is more plausibly explained with the study drug. Positive dechallenge and rechallenge (if applicable). |

The investigator should assess the correlation between the study drug with the AE as "Yes" or "No"

If there is a reasonable suspicion of a correlation between the AE and the study drug, i.e., there are facts (signs) or justifications to support it, then the correlation of the AE should be assessed as "Yes".

To obtain this result of "Yes", the following criteria should be met:

- There is a plausible temporal relationship with the medication.
- Following a known pattern of response to the study drug.
- Symptoms resolve or resolve when the dose is interrupted or reduced
- Symptoms recur after the re-challenge.

To obtain the result of "No", the following criteria should be met:

- There is no plausible temporal association with the study medication.
- Not following a known pattern of response to the study drug.
- Symptoms do not recur or worsen after the re-challenge.

## 8.5 Assessment of Severity

The severity of AE will be evaluated with reference to CTCAE 5.0. If the AEs occurred are not within the criteria, the following Table 10 will be used to assess the severity:

Table 10 Severity Grading Scale for Adverse Events Not Specifically Listed in the NCI-CTCAE

| Grade | Severity |
| --- | --- |
| 1 | Mild: asymptomatic or mild symptoms; clinical or diagnostic observations only; or intervention not indicated |
| 2 | Moderate: minimal, local, or non-invasive intervention indicated; or limiting age‑appropriate instrumental activities of daily living ^a^ |
| 3 | Severe or medically significant, but not immediately life-threatening; hospitalization or prolongation of hospitalization indicated; disabling; or limiting self-care activities of daily living ^b,c^ |
| 4 | Life-threatening consequences or urgent intervention indicated ^d^ |
| 5 | Death related to AE ^d^ |

NCI-CTCAE = National Cancer Institute-Common Terminology Criteria for Adverse Events. Note: Based on the latest version of NCI-CTCAE v5.0, full text available at http://ctep.cancer.gov/protocolDevelopment/electronic_applications/ctc.htm

a Instrumental activities of daily living are defined as preparing meals, shopping for groceries or clothes, using telephones, managing money, etc.

b Self-care activities of daily living include bathing, dressing, having a meal, using the toilet, taking medicines, and not bedridden.

c If an event is rated as "clinically significant", the event must be reported as an SAE as described in Section 8.3.3 (see Section 8.6. 2 for reporting guidance).

d Grade 4 and 5 events must be reported as SAEs as described in Section 8.3. 3 (see Section 8.6.2 for reporting guidance)

## 8.6 Recording and Reporting of AEs

At each required follow-up visit after signing of informed consent during the trial, all AEs and SAEs that have occurred since the previous follow-up visit must be recorded until 60 days after the last dose of the study drug or initiation of new anticancer therapy, whichever occurs first. The investigator must determine the severity of each AE and the assessment of the correlation between each AE with the study drug (see Sections 8.4 and 8.5).

### 8.6.1 Recording and Reporting of AEs

For all AEs occurring during the clinical study, regardless of whether they are suspected to be related to the study drug, the following actions should be taken according to the China GCP:

1. The investigator should immediately take appropriate protective measures for subjects to ensure their safety, and experts should make a diagnosis and state the reasons.
2. If the study is discontinued, the investigator should also examine the subject regularly, and complete the date of discontinuation (date of discontinuation of investigational product), the reason for discontinuation, and the detailed process in the eCRF.
3. The investigator should follow up all AEs until any of the following occurs:

- AE resolved or improved to baseline level;
- Confirmation of no further improvement by the investigator;
- Death of the subject;
- Lost contact with the subject;
- Initiation of a new anticancer therapy.

Any AE should be recorded in the CRF and reported in the clinical study report.

If a large number of unexpected adverse reactions related to study medications occur during the study, please they should be immediately reported to the Ethics Committee, National Medical Products Administration, and relevant drug regulatory authorities of the provinces, autonomous regions, and municipalities directly under the central government.

### 8.6.2 Reporting of SAEs

As per the China GCP, for all SAEs occurring during the clinical study, the following measures should be taken in addition to the treatment described in the Overall Plan to Manage Tislelizumab Safety Issues in Section 8.1:

1. The investigator should report SAEs to National Medical Products Administration (NMPA), health administrative authorities, the Sponsor, and the Ethics Committee by telephone within 24 hours, and sign and date the report.
2. The investigator should provide other information (autopsy report, medical records of the termination phase and other necessary information) related to the SAE (including reported death or adverse drug reactions) as required by the sponsor, medical institutions, and the Ethics Committee.
3. The sponsor and the investigator should promptly investigate the SAE that occurred, take necessary measures to ensure the safety, rights and interests of the subjects, and promptly notify the drug regulatory authorities and other investigators involved in the clinical study of the same drug.
4. All SAEs should be followed up until the events are resolved or improved to baseline, death of the subject, loss of contact, or final confirmation by the investigator that the SAE is unrelated to the study treatment.

During the study, all SAEs will be recorded until 60 days after the last dose of the study drug or initiation of new anticancer therapy, whichever occurs first. Thereafter, the investigator will be asked to report all serious adverse events related to the study treatment, regardless of whether they occur after the study medication. In addition, if there is an unresolved AE or abnormal laboratory test result that is considered related to study treatment, the subject will continue to be followed until resolution or return to baseline, the event is assessed as stable by the investigator, the subject is lost to follow-up, withdraws consent, or it is determined that study treatment or study participation is not the cause of the AE.

### 8.6.3 Reporting and Follow-up of Pregnancies

When the investigator becomes aware that a female subject is pregnant, the investigator must withdraw the subject and follow up until the termination of pregnancy or delivery. When the investigator becomes aware that a male subject's partner is pregnant, the investigator must follow the pregnancy as much as possible. The investigator should immediately report all pregnancies to the sponsor

## 8.7 Recording Procedures for AEs

When recording AEs on the eCRF of AEs, investigators should use accurate medical terms/concepts to avoid verbal language or abbreviations.

Only one AE term can be filled in the event field of the eCRF AE form.

### 8.7.1 Infusion-related Adverse Reactions

Adverse events that occur during or within 24 hours of study drug administration and are judged to be related to the infusion of the study drug should be recorded as diagnoses on the eCRF of AEs (e.g., infusion-related adverse reactions). Use of terms of equivocal significance, such as "systemic reactions", should be avoided whenever possible. If a subject experiences both local and systemic reactions after the same dose of the study drug, each reaction should be recorded separately on the eCRF AE form.

### 8.7.2 Diagnosis versus Signs and Symptoms

In addition to the individual signs and symptoms, the diagnosis (if known) should be recorded in the eCRF AE form, e.g., only hepatic failure or hepatitis, rather than jaundice, flapping tremor, and transaminases increased, should be recorded. However, if a single medical diagnosis cannot be made for a group of signs and/or symptoms or diagnosed as a syndrome upon submission, each event should be recorded separately on the eCRF AE form. If a diagnosis is later established, all events based on previous signs and symptoms should be withdrawn and replaced with an AE report based on a single diagnosis. The start date of the event is the start date of the first symptom of the final diagnosis.

### 8.7.3 Adverse Events Secondary to Other Events

In general, AEs secondary to other events (e.g., cascading events or clinical sequelae) should have their primary cause determined, except for severe or serious secondary events. If a secondary AE is medically significant and is temporally independent of each other, it should be recorded as a separate event on the eCRF of AEs. For example:

- If vomiting results in mild dehydration in a healthy adult with no additional treatment indicated, only vomiting should be reported in the eCRF.
- If vomiting results in severe dehydration, these two events should be reported separately in the eCRF.
- If severe hemorrhage of digestive tract results in renal failure, these two events should be reported separately in the eCRF.
- If dizziness results in a fall followed by a fracture, these three events should be reported separately in the eCRF.
- If reduced neutrophil count results in infection, these two events should be reported separately in the eCRF.

If the correlation between events is unclear, all relevant AEs should be recorded separately on the AE page of the eCRF.

### 8.7.4 Persistent or Recurrent Adverse Events

Persistent AEs refer to AEs that persist and do not resolve between assessment time points for each subject. Such events only need to be recorded once on the eCRF of AEs. The initial severity (severity or grade) of the event will be recorded at the time the event is first reported. If a persistent AE worsens, the maximum severity should be recorded on the AE page of the eCRF. If an AE meets the criteria for an SAE, the investigator should report it immediately to the sponsor (i.e., within 24 hours of learning that the event had worsened; see Section 8.5.2 for reporting instructions). The eCRF of AEs should be updated by changing the severity of the event from "non-serious" to "serious", providing the date the event became serious, and completing all data pertaining to the SAE.

A recurred AE refers to an AE that resolves between two assessment time points but recurs later. Each recurrence of an AE should be recorded separately on the eCRF of AEs.

### 8.7.5 Abnormal Laboratory Values

Not every abnormal laboratory value is an AE. Abnormal laboratory findings must be reported as AEs if they meet any of the following criteria:

- Accompanied by clinical symptoms
- Results in a change in study treatment (e.g., dose modification, treatment interruption or treatment discontinuation)
- Results in medical intervention indicated (e.g., potassium supplementation for hypokalaemia) or changes in concomitant medications
- Results considered clinically significant by the investigator

Note: For oncology studies, some abnormal values do not need to be reported as AEs.

The investigator is responsible for reviewing all laboratory test results. Training in medical and scientific judgment should be conducted to properly determine whether an isolated abnormal laboratory finding should be considered an AE.

If a clinically significant abnormal laboratory finding is a sign of a disease or syndrome (e.g., ALP and bilirubin 5 × ULN associated with cholestasis), only the diagnosis (i.e., cholestasis) needs to be recorded on the AE page of the eCRF.

If a clinically significant abnormal laboratory finding is not a sign of a disease or syndrome, the abnormality itself should be recorded on the AE page of the eCRF with a description indicating whether the test result is above or below the normal range (e.g., "high potassium" rather than "potassium abnormal"). If an abnormal laboratory finding can be expressed as a precise clinical term as defined by the criteria, the clinical term should be recorded as the AE. For example, an increase in serum potassium to 7.0 mEq/L should be recorded as “hyperkalemia.”

Observations of the same clinically significant abnormal laboratory finding from a visit to visit should only be recorded once on the AE page of the eCRF (see Section 8.8.4 for details on recording persistent AEs).

### 8.7.6 Abnormal Vital Sign Values

Not every abnormal vital sign value is an AE. Vital sign abnormalities must be reported as AEs if they meet any of the following criteria:

- Accompanied by clinical symptoms
- Results in a change in study treatment (e.g., dose modification, treatment interruption or treatment discontinuation)
- Results in medical intervention indicated or changes in concomitant medications
- Results considered clinically significant by the investigator

The investigator is responsible for reviewing all vital signs. Training in medical and scientific judgment should be conducted to properly determine whether an isolated abnormal vital sign value should be considered an AE.

If a clinically significant abnormal vital sign value is a sign of a disease or syndrome (e.g., hypertension), only the diagnosis (i.e., hypertension) needs to be recorded on the AE page of the eCRF.

If the clinically significant abnormal vital sign value can be observed at each visit, it should not be recorded repeatedly on the AE page of the eCRF unless the etiology changes (see Section 8.7.4 for details on recording persistent AEs).

### 8.7.7 Abnormal Liver Function Test

The finding of an elevated ALT or AST (> 3 × ULN), elevated total bilirubin (> 2 × ULN), or clinical jaundice in the absence of cholestasis or other etiology of hyperbilirubinemia should be considered suggestive of severe liver injury (as defined by Hy's Law). Therefore, the investigator must report any of the following as AEs:

- Treatment-emergent ALT or AST > 3 × baseline with total bilirubin > 2 × ULN (direct bilirubin ≥ 35%)
- Treatment-emergent ALT or AST > 3 × baseline with clinical jaundice

The most appropriate diagnosis or abnormal laboratory findings (if a definitive diagnosis is not available) should be recorded as an SAE on the AE page of the eCRF and reported to the sponsor immediately (i.e., within 24 hours of awareness).

### 8.7.8 Death

The endpoint in this protocol is death. For deaths occurring within the protocol-specified AE reporting period (see Sections 8.6.1 and 8.6.2), they should be recorded in the listing of deaths attributed to disease progression in the eCRF if judged by the investigator to be due to cancer progression. All other deaths, whether or not related to the study, must be entered on the AE page of the eCRF and reported immediately to the sponsor (see Section 8.5.2).

Death should be considered an outcome rather than a separate event. An event or condition leading to the outcome of death should be entered as a single medical concept on the AE page of the eCRF. In general, only one such event should be reported. The term "sudden death" is used only in cases where sudden and unexpected death is presumed to be of cardiac origin. If the cause of death is unknown or cannot be determined at the time of reporting, it should be recorded as "death unexplained" on the AE page of the eCRF. If the cause of death is later specified (e.g., after autopsy), the phrase "death unexplained" should be changed to "death due to a specified cause".

Deaths attributed to cancer progression during survival follow-up should be recorded in the listing of deaths attributed to disease progression in the eCRF.

### 8.7.9 Pre-existing Diseases

A pre-existing disease means that it is present at the screening visit in this study. This disease should be recorded on the General Medical History and Baseline Physical Condition page of the eCRF.

A pre-existing medical condition should be recorded as an AE only if it worsens in frequency, severity, or nature during the study. When recording such events on the AE page of the eCRF, attention should be paid to conveying the concept that a change in pre-existing medical condition has occurred (e.g., "more frequent headaches") with appropriate descriptions.

### 8.7.10 Lack of Efficacy

Events that are consistent with the expected pattern of progression of the pre-existing disease should not be recorded as AEs. These data will be collected as efficacy analysis data only. In most cases, the expected pattern of progression will be assessed based on radiological assessment criteria such as RECIST v1.1. While in a few cases, clinical progression will be based on symptomatic deterioration. However, progression should be confirmed using objective criteria whenever possible. Events that are uncertain whether they are attributable to disease progression should be reported as AEs.

### 8.7.11 In-patient Hospitalization or Prolongation of Existing Hospitalization

Any AE that results in hospitalization (i.e., subject hospitalization) or prolongation of existing hospitalization should be recorded and reported as an SAE (per the definition of SAEs in Section 8.3), with the following exceptions.

The following hospitalizations will not be considered AEs:

- Hospitalizations for temporary medical care
- Protocol-specified scheduled hospitalizations (e.g., for study drug administration or efficacy assessments)
- Hospitalizations for pre-existing conditions provided that all of the following criteria are met: Hospitalization planned before the study, or patients require elective surgery due to the expected normal disease progression without an AE during the study
- Hospitalizations for related cancer progression only

The following hospitalizations will not be considered SAEs, but should be reported as AEs:

- Hospitalizations for outpatient medical treatment beyond the normal working hours

### 8.7.12 Adverse Events Associated with Overdose or Drug Administration Errors

An overdose is defined as an accidental or intentional administration at a higher dose level than that currently under study. Neither overdose nor incorrect administration of the study drug itself is considered an AE, but it may cause an AE. Adverse events associated with an overdose or incorrect administration of the study drug will be recorded as an SAE on the AE page of the eCRF and reported to the sponsor immediately (i.e., within 24 hours of awareness; see Section 8.5.2 for reporting instructions).

1. Data Management

## 9.1 Data Entry

In this study, the subject data will be entered into a designated electronic case report form (eCRF) and transferred to the data system validated by the sponsor for integration with data from other sources.

Clinical data will be managed in accordance with applicable CDISC standards and data cleansing procedures to ensure data integrity, such as the removal of errors and inconsistent data. AEs and concomitant medications will be encoded based on the Medical Dictionary for Regulatory Activities (MedDRA). The eCRFs will be retained by the sponsor and a duplicate copy will be sent to the investigator.

The site staff is responsible for completing the eCRF. The investigator or an authorized staff member must carefully complete the items in the eCRF for all subjects who sign the ICF and are proven to be eligible after screening. No items shall be left blank or missed (UK/NA/ND should be completed according to the actual situation when there is no content available). All data in the eCRF must be checked against the subject's original records to ensure correctness.

The investigator must attach original copies or photocopies of laboratory test reports to the subject's study medical record. For abnormal laboratory data, the investigator must verify and indicate whether it is clinically significant. The investigator should strictly follow the eCRF Completion Instructions.

## 9.2 Database Lock

The database can be locked when the following conditions are met:

1. All data have been entered into the database;
2. All queries have been resolved;
3. The analysis population is defined and determined.

No change will be made to the locked data documents.

1. Statistical Analysis

The Statistical Analysis Plan should be developed after the finalization of the protocol and finalized before the database lock. The Statistical Analysis Plan will specify and describe all the planned statistical analyses in detail according to the main characteristics of the protocol. All statistical analyses will be performed by the Clinical Data Analysis Department using SAS statistical analysis software.

In this study, all variables obtained at each observation time point will be statistically described by dose group, unless it is not required at a specific time point as specified in the protocol. In general, continuous variables (e.g., age) will be described statistically using the number of observations, mean, median, standard deviation, minimum, and maximum; categorical variables will be described statistically using the frequency and percentage of each category. The final analysis of the study will be based on data collected from subjects throughout the study. The statistical methods will be detailed in the Statistical Analysis Plan.

## 10.1 Calculation of Sample Size

The main study endpoint is Major Pathology Remission Rate (MPR). This study presupposes that MPR can be increased from 13% to 33% after receiving neoadjuvant chemotherapy plus PD-1 inhibitor treatment, with Alpha (α) of 0.025 (one-sided) and test power of 80%. The sample size formula for a single sample rate shows that 28 subjects are needed for each group. Considering the drop-out rate of 10%, it is expected that a total of 32 subjects are planned.

## 10.2 Data Analysis Sets

**Full analysis set:** Subjects who have received at least one perioperative treatment and have follow-up data after medication.

**Efficacy evaluable analysis set:** Subjects who have received at least one perioperative treatment, baseline tumor assessment data, and at least one post-baseline tumor assessment data.

**Safety analysis set:** Subjects who have received at least one perioperative treatment. This analysis set will be used for safety analysis.

Protocol Compliance Analysis Set: Subjects included in the Efficacy Evaluable Analysis Set with no significant protocol violations are included. This dataset includes subjects who are compliant, do not take any prohibited medications during the trial, and complete the trial as required.

## 10.3 Analytical Methods

The Statistical Analysis Plan should be developed after the finalization of the protocol and finalized before the database lock. The Statistical Analysis Plan will specify and describe all the planned statistical analyses in detail according to the main characteristics of the protocol. In general, continuous variables (e.g., age) will be described statistically using the number of observations, mean, median, standard deviation, minimum, and maximum; categorical variables will be described statistically using the frequency and percentage of each category. The final analysis of the study will be based on data collected from subjects throughout the study. The statistical methods will be detailed in the Statistical Analysis Plan. Demographic and baseline disease characteristics will be summarized using the analytical methods described above.

### 10.3.1 Safety

All safety data will be summarized based on the Safety Analysis Set.

Adverse events will be summarized using MedDRA preferred term and SOC. The summary of the relatedness of SAEs and AEs, the severity of AEs, immune-related adverse reactions, and some special events will be listed respectively.

The summary of vital signs data, ECG data, laboratory data, and physical examination data will be presented by time listings.

### 10.3.2 Efficacy

Efficacy analyses will be based primarily on the full analysis set and the efficacy evaluable analysis set.

Efficacy Endpoints:

According to AJCC/UICC 8th edition TNM staging, MPR, and TRG, the pathological evaluation will be performed on the gross specimens by the dedicated pathologist. Imaging (CT or MRI) methods will be used to evaluate tumor response in subjects with measurable diseases according to RECIST 1.1, and tumor immunotherapy response assessment information will also be provided. The same measurements as the baseline will be used throughout the study. The primary efficacy variables are defined as follows:

- MPR, major pathological remission rate, defined as residual tumour lesions less than 10%.
- TRG Grade 0/1 is defined as no residual tumor cells (TRG Grade 0) or only a single or small focus of residual cancer cells (TRG Grade 1).
- pCR is defined as the absence of residual tumor cells in gross tumor specimens (i.e., stage ypT0).
- R0 resection is defined as the absence of gross and microscopically positive margins.
- ORR is defined as the proportion of subjects with complete response (CR) or partial response (PR) radiologically. Subjects with no assessments will be considered non-responders.
- DCR is defined as the proportion of subjects with confirmed CR + PR + SD after completion of neoadjuvant therapy.
- RFS is defined as the interval between the date of first cycle of treatment and the date of the first documentation of disease recurrence or metastasis, or death from any cause, whichever occurs first. Subjects without any event during follow-up or study treatment will be censored at the last tumor assessment. Subjects without a post-baseline assessment will be censored on the date of enrolment.
- EFS is defined as the interval between the date of first cycle of treatment and the date of first documented disease progression precluding surgery, distant metastatic disease or local recurrence, or death from any cause, whichever occurs first.
- OS is defined as the time from first cycle of treatment to death due to any cause. Subjects without any event will be censored at the date last known to be alive. Subjects without any follow-up information will be censored at the date of enrollment.
- Quality of life, was investigated in patients using the EORTC QLQ-C30 (version 3), EORTC QLQ-STO22, and EQ-5D-5L questionnaires.

At the end of the study, using the Clopper-Pearson method to calculate 95% confidence intervals for MPR, ORR, etc.

More statistical analysis methods for efficacy endpoints will be specified in the statistical analysis plan.

### 10.3.3 Exploratory Analysis

The following endpoints will be summarized based on the efficacy evaluable analysis set. Including but not limited to:

- Correlation between PD-L1 expression at baseline and efficacy (ORR) of perioperative chemotherapy plus Tislelizumab and postoperative MPR;
- Correlation between EBV expression in tumor tissue and blood at baseline and efficacy (ORR) of perioperative chemotherapy plus Tislelizumab and postoperative MPR;
- Correlation between MSI expression in tumor tissues at baseline and efficacy (ORR) of perioperative chemotherapy plus Tislelizumab and postoperative MPR;
- Correlation analysis of exploring the relationship between other potential immune predictors and anti-tumor activity of chemotherapy plus Tislelizumab
- In addition, subjects who voluntarily undergo tumor tissue sampling for optional biomarker study in case of disease progression will be summarized;

## 10.4 Interim Analysis

Interim analysis was performed when half of the subjects were enrolled. Interim analyses include efficacy analysis and safety analysis. The specific analysis methods and required charts will be detailed in the Interim Analysis Plan.

1. Study Management

This study will be conducted in strict compliance with the Chinese laws and regulations related to clinical studies, including GCP, Declaration of Helsinki (Version 2008) and other regulations, as well as this study protocol. The specific study procedures will be controlled by the standard operating procedures of all parties involved in the study.

## 11.1 Ethical Considerations

The protocol, ICF, eCRF, and other materials must be submitted to the IRB/IEC for approval before the study is initiated. The IRB/IEC will review and approve these materials in strict accordance with the relevant laws and regulations, and issue the approval letter after review. The study will be initiated only after approval from the IRB/IEC has been received.

During the study, any amendments to the protocol must also be reviewed and approved by the IRB/IEC before implementation.

## 11.2 Informed Consent

The investigator or his/her designated representative will be responsible for explaining the study background, pharmacological characteristics of the study drug, the protocol, and benefits and risks of participating in the study to the subject or his/her legally acceptable representative. A written informed consent form signed jointly by the subject or his/her legally authorized representative and the study physician should be obtained before the subject enters the study (prior to screening).

The final ICF text should contain the following: study objectives, study procedures, subject's obligations, foreseeable benefits as well as risks and inconveniences for subjects participating in the study; treatment and appropriate insurance compensation available to be provided for subjects if study-related impairment occurs; access to study data and confidentiality of subjects' information, etc. Written approval of the ICF should be obtained from regulatory authorities in accordance with applicable laws and regulations, and the informed consent form should be written in a language readable to the subjects.

The ICF should be signed and dated by the subject or his/her legally acceptable representative and the investigator performing the informed consent process or his/her representative. Both the investigator and the subject should maintain one copy of the ICF. The ICF should be revised in writing whenever new important study drug-related information becomes available, submitted for approval of regulatory authorities, and re-consenting of the subjects is required.

## 11.3 Compensation for Health Damage

The sponsor will bear the treatment cost and provide corresponding economic compensation according to the relevant national laws and regulations for the injury that is causally related to the study treatment caused by the participation in this study. The sponsor will not compensate for the injury caused by medical malpractice or failure to comply with the study protocol.

## 11.4 Recording and Retention of Study Data

To ensure the evaluation and supervision of NMPA and the sponsor, the investigator should agree to save all study data, including but not limited to confirmation records of all subjects (for effectively checking all records, such as eCRFs and original hospital records), all original signed ICFs, all eCRFs, and drug distribution details. The retention period is 5 years after the end of the study or until the sponsor notifies destruction.

All data generated in this clinical study is a property owned by the sponsor, and should not be provided to a third party by the investigators without the sponsor’s written approval, unless requested by CFDA.

## 11.5 Return or Destruction of Study Drugs/Therapeutic Supplies

After the end of the study, all study drugs/therapeutic supplies should be returned to the sponsor or destroyed at the study site according to written instructions from the sponsor.

Destruction of study drugs/therapeutic supplies at the study site must be performed in the presence of a sponsor representative or by appropriate personnel designated by the sponsor. If it is performed by a designated person, a formally signed certificate of destruction should be completed and delivered to the sponsor.

## 11.6 Quality Control and Quality Assurance

In order to ensure the quality of the study, the sponsor and the investigator jointly discuss and develop a clinical study plan before the official start of the study. GCP training will be provided to all relevant study staff involved in the study.

The study drugs must be managed by the study site in accordance with standard operating procedures (SOPs), including receipt, storage, dispensing and return.

### 11.6.1 Monitoring and Audit

Monitoring:

The clinical monitor authorized by the sponsor has the right to access eCRF, ICF and all source data at any time.

The clinical monitor will be responsible for developing the plan and procedures to be followed for monitoring the study. An on-site visit will be performed prior to the start of the study. Regular visits are required during the study. Contact may be made by phone, fax, or email as needed to supplement on-site visits.

Investigators will be informed of the expected frequency of monitoring visits prior to the start of the study. In addition, the investigator will be notified in advance of each monitoring visit during the study. The purpose of the visit is to ensure: 1) the clinical study is carried out in strict compliance with the study protocol, and 2) the completeness and accuracy of the case report form can be verified against the source documents.

The clinical monitor will verify that all eCRFs are completed correctly and completely and consistent with the original data; all errors or omissions are corrected or noted, signed and dated by the Investigator. At each visit, close cooperation between the investigator and the clinical monitor is required to review and confirm the case report form, drug supply and inventory records, drug distribution and recovery records, and any other arranged additional records.

The sponsor or authorized personnel of the sponsor may review the quality of this study, and the reviewer has the right to inspect all study-related medical records, investigator site file (ISF), communications and ICFs.

Audit:

Auditors may audit this study as appropriate per relevant SOPs to ensure that the study is conducted per relevant national regulations, SOPs and protocol. The scope of the audit includes office documents and site documents. The auditor will provide a written report explaining the problems found and making suggestions after the audit. Relevant personnel (investigator and monitor) should take corresponding corrective measures and record them in writing.

## 11.7 Amendments to the Study Protocol

Any significant amendments to the protocol should be approved by the sponsor and the investigator in written form and submitted to the IRB/IEC for approval and CFDA for filing before implementation.

## 11.8 Protocol Violation

Subjects who clearly deviate from the specified criteria in the protocol but are enrolled inadvertently should be withdrawn from the study.

The investigator should inquire whether the subject has had any concomitant medication since the last visit at each return visit, to determine whether the treatment violates the protocol, and record this information at the same time.

## 11.9 Study Termination

The sponsor reserves the right to terminate the study at any time for medical reasons or any other reason. If the study is terminated prematurely or interrupted, the sponsor should immediately notify the investigator of the study termination or interruption and explain the reasons. The sponsor or investigator should also promptly notify the IRB/IEC of the study termination or interruption and explain the reasons as required by applicable regulations.

The investigator reserves the right to determine whether the study should be terminated. If the investigator terminates or interrupts the study without prior consent from the sponsor, he/she should promptly notify the sponsor and the IRB/IEC and provide them with a detailed written explanation report. Study records must be maintained.

## 11.10 Study Summary Report

After the end of the study, the investigator and the sponsor will objectively summarize the study results, conduct a statistical analysis of the study data with appropriate statistical methods, and make an objective evaluation of the safety of the drug based on the study results. A written summary report of this clinical study will be made upon review and approval by the sponsor.

## 11.11 Confidentiality and Publication of Study Results

The investigator should keep the information and data related to the study confidential, and cannot cite or publish relevant study results or information without the approval of the sponsor.

The sponsor is entitled to make public or publish study-related information or data, or submit it to NMPA. If the sponsor needs to include the investigator's name in a published paper, publication or advertisement, the consent of the investigator should be obtained.

1. References

1. Bray F, Ferlay J, Soerjomataram I et al. Global cancer statistics 2018: GLOBOCAN estimates of incidence and mortality worldwide for 36 cancers in 185 countries. CA Cancer J Clin 2018; 68: 394-424.

2. Yang L, Zheng R, Wang N et al. Incidence and mortality of stomach cancer in China, 2014. Chin J Cancer Res 2018; 30: 291-298.

3. Siegel RL, Miller KD, Jemal A. Cancer statistics, 2020. CA Cancer J Clin 2020; 70: 7-30.

4. Liu K, Yang K, Zhang W et al. Changes of Esophagogastric Junctional Adenocarcinoma and Gastroesophageal Reflux Disease Among Surgical Patients During 1988-2012: A Single-institution, High-volume Experience in China. Ann Surg 2016; 263: 88-95.

5. Colquhoun A, Arnold M, Ferlay J et al. Global patterns of cardia and non-cardia gastric cancer incidence in 2012. Gut 2015; 64: 1881-1888.

6. Pedrazzani C, de Manzoni G, Marrelli D et al. Lymph node involvement in advanced gastroesophageal junction adenocarcinoma. The Journal of thoracic and cardiovascular surgery 2007; 134: 378-385.

7. Feith M, Stein HJ, Siewert JR. Adenocarcinoma of the esophagogastric junction: surgical therapy based on 1602 consecutive resected patients. Surgical oncology clinics of North America 2006; 15: 751-764.

8. Takeuchi H, Kitagawa Y. Adenocarcinoma of the esophagogastric junction: territory of the esophagus or stomach, or an independent region? Ann Surg Oncol 2013; 20: 705-706.

9. Ajani JA, D'Amico TA, Bentrem DJ et al. Esophageal and Esophagogastric Junction Cancers, Version 2.2019, NCCN Clinical Practice Guidelines in Oncology. Journal of the National Comprehensive Cancer Network : JNCCN 2019; 17: 855-883.

10. Wang W, Sun Z, Deng JY et al. Integration and analysis of data related to surgical treatment of gastric cancer based on a multicenter large sample database. Chinese Journal of Gastrointestinal Surgery 2016; 179-185.

11. Cunningham D, Allum WH, Stenning SP et al. Perioperative chemotherapy versus surgery alone for resectable gastroesophageal cancer. N Engl J Med 2006; 355: 11-20.

12. Ychou M, Boige V, Pignon JP et al. Perioperative chemotherapy compared with surgery alone for resectable gastroesophageal adenocarcinoma: an FNCLCC and FFCD multicenter phase III trial. J Clin Oncol 2011; 29: 1715-1721.

13. Sasako M, Sakuramoto S, Katai H et al. Five-year outcomes of a randomized phase III trial comparing adjuvant chemotherapy with S-1 versus surgery alone in stage II or III gastric cancer. J Clin Oncol 2011; 29: 4387-4393.

14. Noh SH, Park SR, Yang H-K et al. Adjuvant capecitabine plus oxaliplatin for gastric cancer after D2 gastrectomy (CLASSIC): 5-year follow-up of an open-label, randomised phase 3 trial. The Lancet Oncology 2014; 15: 1389-1396.

15. Songun I, Putter H, Kranenbarg EM et al. Surgical treatment of gastric cancer: 15-year follow-up results of the randomised nationwide Dutch D1D2 trial. Lancet Oncol 2010; 11: 439-449.

16. Sasako M, Sano T, Yamamoto S et al. D2 lymphadenectomy alone or with para-aortic nodal dissection for gastric cancer. N Engl J Med 2008; 359: 453-462.

17. Keir ME, Butte MJ, Freeman GJ, Sharpe AH. PD-1 and its ligands in tolerance and immunity. Annu Rev Immunol 2008; 26: 677-704.

18. Pardoll DM. The blockade of immune checkpoints in cancer immunotherapy. Nat Rev Cancer 2012; 12: 252-264.

19. Ribas A, Wolchok JD. Cancer immunotherapy using checkpoint blockade. Science 2018; 359: 1350-1355.

20. Muro K, Chung HC, Shankaran V et al. Pembrolizumab for patients with PD-L1-positive advanced gastric cancer (KEYNOTE-012): a multicentre, open-label, phase 1b trial. The Lancet Oncology 2016; 17: 717-726.

21. Fuchs CS, Doi T, Jang RW et al. Safety and Efficacy of Pembrolizumab Monotherapy in Patients With Previously Treated Advanced Gastric and Gastroesophageal Junction Cancer. JAMA Oncology 2018; 4: e180013.

22. Kang YK, Boku N, Satoh T et al. Nivolumab in patients with advanced gastric or gastro-oesophageal junction cancer refractory to, or intolerant of, at least two previous chemotherapy regimens (ONO-4538-12, ATTRACTION-2): a randomised, double-blind, placebo-controlled, phase 3 trial. Lancet 2017; 390: 2461-2471.

23. Shitara K, Ozguroglu M, Bang YJ et al. Pembrolizumab versus paclitaxel for previously treated, advanced gastric or gastro-oesophageal junction cancer (KEYNOTE-061): a randomised, open-label, controlled, phase 3 trial. Lancet 2018.

24. Janjigian YY, Shitara K, Moehler M et al. First-line nivolumab plus chemotherapy versus chemotherapy alone for advanced gastric, gastro-oesophageal junction, and oesophageal adenocarcinoma (CheckMate 649): a randomised, open-label, phase 3 trial. The Lancet 2021; 398: 27-40.

25. Boku N, Ryu MH, Oh DY et al. Nivolumab plus chemotherapy versus chemotherapy alone in patients with previously untreated advanced or recurrent gastric/gastroesophageal junction (G/GEJ) cancer: ATTRACTION-4 (ONO-4538-37) study. Annals of Oncology 2020; 31: S1192.

26. Forde PM, Chaft JE, Smith KN et al. Neoadjuvant PD-1 Blockade in Resectable Lung Cancer. N Engl J Med 2018; 378: 1976-1986.

27. Kojima T, Shah MA, Muro K et al. Randomized Phase III KEYNOTE-181 Study of Pembrolizumab Versus Chemotherapy in Advanced Esophageal Cancer. J Clin Oncol 2020; 38: 4138-4148.

28. Huang J, Xu J, Chen Y et al. Camrelizumab versus investigator's choice of chemotherapy as second-line therapy for advanced or metastatic oesophageal squamous cell carcinoma (ESCORT): a multicentre, randomised, open-label, phase 3 study. The Lancet Oncology 2020; 21: 832-842.

29. Luo H, Lu J, Bai Y et al. Effect of Camrelizumab vs Placebo Added to Chemotherapy on Survival and Progression-Free Survival in Patients With Advanced or Metastatic Esophageal Squamous Cell Carcinoma: The ESCORT-1st Randomized Clinical Trial. JAMA 2021; 326: 916-925.

30. Kim ST, Cristescu R, Bass AJ et al. Comprehensive molecular characterization of clinical responses to PD-1 inhibition in metastatic gastric cancer. Nat Med 2018; 24: 1449-1458.

31. McDermott DF, Huseni MA, Atkins MB et al. Clinical activity and molecular correlates of response to atezolizumab alone or in combination with bevacizumab versus sunitinib in renal cell carcinoma. Nat Med 2018; 24: 749-757.

Appendix 1 AJCC/UICC TNM Staging Criteria Version 8 for Gastric Cancer

| T stage | Tx | Primary tumor cannot be evaluated |
| --- | --- | --- |
|  | T0 | No evidence of primary tumor |
|  | Tis | Carcinoma in situ: intraepithelial neoplasm without reaching the lamina propria |
|  | T1 | Tumor invades lamina propria or muscularis mucosae (T1a) or submucosa (T1b) |
|  | T2 | Tumor invades muscularis propria |
|  | T3 | Tumor penetrates the subserosal connective tissue without invasion of the visceral peritoneum or adjacent structures* |
|  | T4 | Tumor invades serosa (T4a) or adjacent structures(T4b) **, *** |
| N stage | Nx | Regional lymph nodes cannot be evaluated |
|  | N0 | No regional lymph node involvement# |
|  | N1 | Metastasis in 1 to 2 regional lymph nodes |
|  | N2 | Metastasis in 3 to 6 regional lymph nodes |
|  | N3 | Metastasis in 7 or more (N3b) regional lymph nodes:  N3a: Metastasis in 7-15 regional lymph nodes  N3b: Metastasis in 16 or more regional lymph nodes |
| M stage | M0 | No distant metastasis |
|  | M1 | Distant metastasis |

* A tumor may penetrate the muscularis propria with extension into the gastrocolic or gastrohepatic ligaments, or into the greater or lesser omentum, without perforation of the visceral peritoneum covering these structures. In this case, the tumor is classified T3. If there is perforation of the visceral peritoneum covering the gastric ligaments or the omentum, the tumor should be classified T4.

**Clinical Stage (cTNM)**

| 0 | Tis | N0 | M0 |
| --- | --- | --- | --- |
| I | T1-2 | N0 | M0 |
| IIA | T1-2 | N1-2 | M0 |
| IIB | T3-4a | N0 | M0 |
| III | T3-4a | N1-3 | M0 |
| IVA | T4b | Any N | M0 |
| IVB | Any T | Any N | M1 |

**Staging after neoadjuvant therapy (ypTNM)**

| I | T1-2 | N0 | M0 |
| --- | --- | --- | --- |
|  | T1 | N1 | M0 |
| II | T1 | N2-3 | M0 |
|  | T2 | N1-2 | M0 |
|  | T3 | N0-1 | M0 |
|  | T4a | N0 | M0 |
| III | T2 | N3 | M0 |
|  | T3 | N2-3 | M0 |
|  | T4a | N1-3 | M0 |
|  | T4b | N0-3 | M0 |
| IV | Any T | Any N | M1 |

Appendix 2 CT Staging of Gastric Cancer

**T stage:**

T1a Invasion of mucosal layer CT shows relatively high enhancement on the mucosal side and/or thickening relative to adjacent normal mucosa with complete hypoattenuating submucosal stripe under the mucosa.

T1b Invasion of submucosa CT shows invasion of hypoattenuating submucosal stripe with a depth of less than 50%.

T2 Invasion of muscularis propria CT shows invasion of the submucosal low-density band with a depth of more than 50%, but not reaching the high enhancement layer of the serosal side.

T3 Invasion of the subserous layer Highly enhanced cancer and outer gastric wall can be distinguished, and mildly blurred but generally smooth outer gastric wall, with a few small linear areas of stranding.

T4a Invasion of serosa Irregular nodular protrusion or spiculated cord strip invasion on the serosal side, or diffusely increased density in the surrounding peritoneal fat.

T4b Invasion of adjacent organs the fat space between the lesion and adjacent organ structures disappeared, and signs of impaction, infiltration and extension of adjacent structures or cord traction appeared.

**N stage**

At present, there is no uniformly accepted standard for lymph node metastasis. In this study, "lymph node short axis ≥ 8 mm, especially round, and arterial phase enhancement or necrosis" is used to diagnose lymph node metastasis.

Appendix 3 NCI Response Evaluation Criteria in Solid Tumors (RECIST Version 1.1)

At baseline, tumor lesions/lymph nodes will be categorized as measurable or non-measurable as follows:

1. Measurable: tumor lesions: at least one diameter that can be accurately measured (recorded as the largest diameter); malignant lymph nodes: pathologically enlarged and measurable, and an individual lymph node must be ≥ 15 mm in the short axis by CT scan (CT scan slice thickness recommended to be no greater than 5 mm).

2. Non-measurable: All other lesions, including small lesions (longest diameter < 10 mM or pathological lymph nodes with ≥ 10 mM to < 15 mM short axis) as well as truly non-measurable lesions. Lesions considered non-measurable include: leptomeningeal disease, ascites, pleural or pericardial effusion, inflammatory breast disease, carcinomatous lymphangitic of skin or lung, abdominal masses/abdominal organomegaly identified by a physical exam that is not measurable by reproducible imaging techniques, and cystic lesions.

3. Methods of Measurement:

(1) CT and MRI: CT is currently the best available and reproducible method for response assessment. This guideline has defined the measurability of lesions on CT scan based on the assumption that CT slice thickness is ≤ 5 mm. When CT scans have a slice thickness greater than 5 mm, the minimum size for a measurable lesion should be twice the slice thickness. MRI is also acceptable in certain situations (e.g., for body scans).

(2) Ultrasound (US): US should not be used as a method of measurement to measure lesion size.

(3) Endoscopy, laparoscopy: The utilization of these techniques for objective tumor evaluation is not advised. However, they can be useful to confirm CR when biopsies are obtained or to determine relapse in trials where recurrence following CR or surgical resection is an endpoint.

(4) Tumor markers: Tumor markers alone cannot be used to assess objective tumor response. If markers are initially above the upper normal limit, however, they must normalize for a patient to be considered in complete response.

4. Lesion baseline recording:

(1) Target lesions: When more than one measurable lesion is present at baseline, all lesions up to a maximum of five lesions total (and a maximum of two lesions per organ) representative of all involved organs should be identified as target lesions and will be recorded and measured at baseline. Target lesions should be selected based on their size (lesions with the longest diameter), and be representative of all involved organs, but in addition, be those that lend themselves to reproducible repeated measurements. Lymph nodes merit special mention since they are normal anatomical structures that may be visible by imaging even if not involved in the tumor. Pathological nodes which are defined as measurable and may be identified as target lesions must meet the criterion of a short axis of P15 mm by CT scan. All other pathological nodes (those with short axis P10 mm but < 15 mm) should be considered non-target lesions. Nodes that have a short axis < 10 mm are considered non-pathological and should not be recorded or followed.

(2) Non-Target Lesions: A sum of the diameters (longest for non-nodal lesions, short axis for nodal lesions) for all target lesions will be calculated and reported as the baseline sum diameters. All other lesions (or sites of disease) including pathological lymph nodes should be identified as non-target lesions and should also be recorded at baseline.

5. Response Criteria:

(1) Target Lesion Assessment

Complete response (CR): Disappearance of all target lesions. Any pathological lymph nodes (whether target or non-target) must have a reduction in short axis to <10 mm.

Partial Response (PR): At least a 30% decrease in the sum of diameters of target lesions, taking as reference the baseline sum diameters.

Progressive disease (PD): At least a 20% increase in the sum of diameters of target lesions, taking as reference the smallest sum in the study (this includes the baseline sum if that is the smallest in the study). In addition, the sum of diameters must also demonstrate an absolute increase of at least 5 mm (the appearance of one or more new lesions is also considered progression).

Stable disease (SD): Neither sufficient shrinkage to qualify for PR nor sufficient increase to qualify for PD, taking as reference the smallest sum diameters while in the study.

(2) Assessment of non-target lesions

While some non-target lesions may actually be measurable, they need not be measured and instead should be assessed only qualitatively at the time points specified in the protocol.

Complete Response (CR): Disappearance of all non-target lesions and normalization of tumor marker level. All lymph nodes are non-pathological in size (< 10 mm short axis).

Non-CR/Non-PD: Persistence of one or more non-target lesion(s) and/or maintenance of tumor marker level above the normal limits.

Progressive Disease: Unequivocal progression of existing non-target lesions. Note: the appearance of one or more new lesions is also considered progression.

|  | **Screening Period** | **Preoperative Treatment Period**  **Q3W (± 3 days)** | | | | | | **Operative period ^17^** | | | **Postoperative Treatment Period ^18^**  **Q3W (± 3 days)** | | **End-of-Treatment Visit** | **Follow-up ^19^** |
| --- | --- | --- | --- | --- | --- | --- | --- | --- | --- | --- | --- | --- | --- | --- |
|  |  | **Cycle 1** | | **Cycle 2** | | **Cycle 3** | | **Preoperative assessment** | | **Surgical assessment** | **Cycle 4** | | **± 3 days** | **Every 3 months** |
|  | **Within -28 days** | **C1D1** | **C1D8** | **C1D1** | **C1D8** | **C1D1** | **C1D8** | | **Within -7 days** | **Within 30 days** | **C1D1** | **C1D8** |  |  |
| **Informed consent ^1^** | X |  |  |  |  |  |  | | X |  |  |  |  |  |
| **Demographic information** | X |  |  |  |  |  |  | |  |  |  |  |  |  |
| **Prior medical history** | X |  |  |  |  |  |  | |  |  |  |  |  |  |
| **Prior tumor history ^2^** | X |  |  |  |  |  |  | |  |  |  |  |  |  |
| **Tumor tissue collection ^3^** | X |  |  |  |  |  |  | |  | X |  |  |  |  |
| **Physical examination ^4^** | X | X | X | X | X | X | X | | X |  | X | X | X |  |
| **Vital signs** | X | X | X | X | X | X | X | | X |  | X | X | X |  |
| **ECOG score** | X | X |  | X |  | X |  | | X |  | X |  | X |  |
| **Laboratory tests ^5^** | X |  | X | X | X | X | X | | X |  | X | X | X |  |
| **Serum Pregnancy test (if applicable)** | X |  |  |  |  |  |  | |  |  |  |  | X |  |
| **INR and aPTT** | X | **As clinically indicated** | | | | | | X | |  |  |  | X |  |
| **Cardiac ultrasound ^6^** | X |  |  |  |  |  |  | | X |  |  |  | X |  |
| **12-lead ECG** | X |  |  | X |  | X |  | | X |  | X |  | X |  |
| **Virological tests ^7^** | X |  |  |  |  |  |  | |  |  |  |  |  |  |
| **Thyroid function ^8^** | X |  |  | X |  | X |  | | X |  | X |  | X |  |
| **Tumor assessment ^9^** | X |  |  |  |  |  |  | | X | X |  |  | X | X |
| **Inclusion/exclusion criteria** | X |  |  |  |  |  |  | |  |  |  |  |  |  |
| **Subject enrollment ^10^** | X |  |  |  |  |  |  | |  |  |  |  |  |  |
| **EBV blood test ^11^** | X |  |  |  |  |  |  | |  |  |  |  |  |  |
| **Blood biomarker tests ^12^** |  | X |  | X |  | X |  | |  |  | X |  | X |  |
| **PD-1 antibody treatment ^13^** |  | X |  | X |  | X |  | |  |  | X |  |  |  |
| **Oxaliplatin ^14^** |  | X |  | X |  | X |  | |  |  | X |  |  |  |
| **Tegafur/gimeracil/oteracil ^14^** |  | **Day 1-14** | | **Day 1-14** | | **Day 1-14** | |  | |  | **Day 1-14** | |  |  |
| **Concomitant Medications/Treatments ^15^** | X | X |  | X |  | X |  | |  |  | X |  | X | X |
| **Adverse Events ^16^** |  | X |  | X |  | X |  | |  | X | X |  | X | X |

Appendix 4 Schedule of Activities (SOA)

**Comments:**

1. The informed consent form must be obtained before all the study-specific procedures, and the surgery-related informed consent form must be additionally signed before surgery;
2. Prior tumor history includes date of tumor diagnosis, start/stop date of prior treatment regimen. Previous significant procedures (such as gastroscopy, puncture biopsy and other diagnostic or therapeutic invasive procedures) should be recorded in the case report form, including start and end dates, name and site of operation;
3. Subjects must provide eligible tumor tissue specimens before enrollment (fresh tumor tissue specimens biopsied before enrollment are preferred) for use in subsequent exploratory studies. Subjects should be tested for PD-L1, EBV, and MSI. The subject consents to the investigator to obtain the surgical tissue specimen for the subsequent exploratory study. Subjects with response evaluation as progressive disease (PD) may be encouraged to voluntarily participate in the optional biomarker study if tumor lesion tissue is available and provide tumor tissue for exploratory study on the correlation between tumor markers and anti-tumor response.
4. Physical examination: including height (only at screening), weight, head, eyes, ears, nose, throat, neck, heart, chest (including lungs), abdomen, extremities, skin, lymph nodes, nervous system and general condition;
5. Laboratory test indicators include hematology, blood chemistry, coagulation function test and urinalysis (hematology: red blood cell count, hemoglobin, hematocrit, white blood cell count and differential [neutrophils, lymphocytes, eosinophils, monocytes, basophils and other cells] and platelet count. Blood chemistry: Blood chemistry includes total protein, albumin, globulin, blood glucose, total cholesterol, low-density lipoprotein, high-density lipoprotein, triglyceride, urea, creatinine, alkaline phosphatase, lactate dehydrogenase, total bilirubin, direct bilirubin, indirect bilirubin, AST, ALT, calcium, phosphorus, magnesium, potassium, sodium, chloride, serum amylase and uric acid. Urinalysis: specific gravity, PH, urine glucose, protein, cast, ketones, blood cells; if urine protein + + or above is tested or the result is abnormal and clinically significant as judged by the doctor, 24-hour urine protein quantitative determination is required. Baseline laboratory tests should be completed within 7 days prior to enrollment. Laboratory tests during the treatment period should be completed before each administration, and the blood collection time should not be earlier than 3 days before administration. The administration can only be started after the laboratory test results meet the criteria for continued administration as determined by the investigator. If the screening hematology and serum chemistry tests are within 3 days (72 hours) of C1D1, the corresponding tests on C1D1 can be exempted.
6. Echocardiography is used to check the left ventricular ejection fraction. They are assessed preoperatively at screening and within 7 days before surgery, and every 12 weeks postoperatively.
7. Virology test includes 5 markers of Hepatitis B (HBsAg, anti-HBs, HBeAg, anti-HBe, anti-HBc) (HBV DNA copies should be detected in case of positive HBsAg), HCV antibody, HIV antibody;
8. Thyroid function test: thyroid stimulating hormone (TSH), serum free triiodothyronine (FT3) and serum free thyroxine (FT4) will be tested once before administration of each cycle and at the end of treatment visit. If clinically significant changes in thyroid function occur, endocrinology consultation and relevant pituitary function tests are recommended.
9. Tumors will be evaluated according to RECIST version 1.1. Tumor assessment at screening must be performed within 3 weeks before enrollment, and chest, abdomen and pelvic cavity CT or MRI scan should be performed. Patients should be clinically staged according to AJCC/UICC Version 8 TNM staging. For patients with cT3-4aN + M0 based on imaging assessment, it is recommended to perform laparoscopic exploration to exclude liver metastasis and abdominal implantation (including positive ascites cytology) if the center is qualified. Imaging examinations conducted for routine evaluation of tumors do not need to be repeated if they are done at the same study site and are within 3 weeks prior to enrollment. Baseline and subsequent imaging should be conducted by the same imaging method (CT or MRI) and evaluated by the same reader, if possible. Imaging assessment will be performed again after 3 courses of preoperative treatment, every 3 months for the first 2 years, every 6 months for the first 3-5 years, and yearly thereafter for tumor recurrence. Postoperative pathological evaluation should be performed according to AJCC/UICC 8th edition ypTNM staging and TRG grading;
10. Subject enrollment: after checking the inclusion/exclusion criteria and confirming the eligibility of the subject, the subject information should be activated by logging in to the EDC system within 3 days prior to administration;
11. EBV blood test: for patients with positive EBER IHC examination at screening period, EBV DNA examination will be performed at screening period, 7 days before the surgery, 6 months, and 12 months after the surgery, respectively.
12. During the PD-1 antibody study treatment period, 3mL of whole blood will be collected and serum will be separated within 30 minutes prior to each dose until the subject completes the end-of-treatment visit.
13. For patients in the test group, PD-1 antibody is administered Q3W. If a subject has discontinued treatment for more than 56 days and the risks of continuing treatment with PD-1 antibody outweigh the benefits as judged by the investigator, permanent withdrawal from study treatment should be considered.
14. The preparation of chemotherapeutic drugs should be conducted according to the package inserts of the drugs used in each center, see 6.2. 3.2 for details. Refer to Section 6.3 for prophylaxis, Section 6.5 for drug dose modification principles, and Section 9.2 for toxicity management and specific drug dose modifications.
15. All drug therapies used within 28 days prior to the enrollment must be recorded in the case report form, including the generic name and daily dose, medication reasons, and the start date and the end date.
16. Adverse events (AEs) should be collected from the time of signing the informed consent form until 60 days after the last dose, and should be recorded on the AE page of the CRF. According to the Clavien-Dindo Classification of Complications, the investigator will evaluate the surgical complications within 30 days after surgery, mainly including hemorrhage of the digestive tract, anastomotic leakage, pancreatic fistula and incision complications (including infection, bleeding and dehiscence).
17. Surgery should be performed within 2-4 weeks after the third course of preoperative chemotherapy or chemotherapy plus PD-1 antibody.
18. Postoperative adjuvant therapy should be started within 4-6 weeks after surgery. Postoperative adjuvant therapy should be given Q3W (see Section 3.2 for details). The total duration of preoperative neoadjuvant therapy and postoperative adjuvant therapy is half a year.
19. Tumor recurrence assessment will be performed every 3 months for the first 2 years after surgery, every 6 months for the first 3-5 years, and annually thereafter.

Appendix 5 Patient Performance Status (ECOG) Scoring Criteria

| **Scores** | **Scoring criteria** |
| --- | --- |
| **0** | Fully active, able to carry on all pre-disease performance without restriction |
| **1** | Restricted in physically strenuous activity but ambulatory and able to carry out work of a light or sedentary nature (eg, light housework, office work). |
| **2** | Ambulatory and capable of all self-care activities but unable to carry out any work activities. Can get up and move around for not less than 50% of waking hours |
| **3** | Capable of only limited self-care, confined to bed or chair for more than 50% of waking hours |
| **4** | Bedridden and unable to carry out any self-care activities |
| **5** | Death |

Appendix 6 Evaluation of Preoperative Adjuvant Therapy for Tumor (Tumor Regression Grade)

| Tumor Regression Grade (TRG) | Microscopic findings |
| --- | --- |
| 0 (complete regression) | No residual tumor cells |
| 1 (Moderate regression) | Only single cells or small groups of residual tumor cells |
| 2 (minor regression) | Presence of residual tumor cells but less than fibrotic stroma |
| 3 (no regression) | Extensive residual tumor with no or a small amount of tumor cell necrosis |

Note: 1)Tumor cells refer to viable tumor cells, excluding degenerative and necrotic cells;

2) Large acellular mucus lakes may appear after radiotherapy/chemotherapy, which cannot be considered as tumor remnants.

Appendix 7 Clavien-Dindo Classification of Complications

| **Classification** | **Definition** |
| --- | --- |
| Grade I | Any deviation from the normal post-operative course not requiring surgical, endoscopic or radiological intervention. This includes the need for certain drugs (e.g. antiemetics, antipyretics, analgesics, diuretics and electrolytes), treatment with physiotherapy and wound infections that are opened at the bedside; |
| Grade II | Complications requiring drug treatments other than those allowed for Grade I complications; this includes blood transfusion and total parenteral nutrition (TPN) |
| Grade III | Complications requiring surgical, endoscopic or radiological intervention |
| Grade IIIa | Intervention not under general anaesthetic |
| Class IIIb | Intervention under general anaesthetic |
| Class IV | Life-threatening complications; this includes CNS complications (e.g. brain haemorrhage, ischaemic stroke, subarachnoid haemorrhage) which require intensive care, but excludes transient ischaemic attacks (TIAs) |
| Grade IVa | Single-organ dysfunction (including dialysis) |
| Grade IVb | Multi-organ dysfuncton |
| Grade Ⅴ | Death |

Appendix 8 Recommendations for Replacement Therapy for Hypothyroidism

Levothyroxine sodium tablets (commonly known as Euthyrox) are recommended for patients who develop study treatment-related hypothyroidism.

The recommended dose in this appendix follows the general principle, and the individual daily dose should be determined according to the laboratory tests and clinical examinations. The basal concentration of thyroid-stimulating hormone in the serum is a reliable basis for determining treatment due to the elevated T4 and fT4 in many patients. Generally, thyroid hormone therapy should be started at the lowest dose and gradually increased every 2-4 weeks until the full dose is reached. Overall, patients with hypothyroidism require lifelong medication.

In elderly patients, patients with coronary artery disease, and patients with severe or prolonged hypothyroidism, particular care should be taken during the initial treatment with thyroid hormone, with a lower initial dose (e.g. 12.5 μg/day). Then, the dose is slowly increased at a longer interval (e.g. increasing the dose by 12.5 μg/day every two weeks).

If a patient's final maintenance dose is below the optimal dose, their TSH level cannot be completely corrected.

Experience has shown that patients with low body weight and those with goiter nodular respond to the low doses.

Levothyroxine sodium tablets should be taken once a day with an appropriate liquid (for example, half a glass of water) under the fasting condition half an hour before breakfast.

**Recommended Dosage Table**

|  | Dose | 50 μg tablets | 100 μg tablets | Frequency of administration |
| --- | --- | --- | --- | --- |
| Initial dose (increase by 25-50 μg every 2-4 weeks after initial dose until maintenance dose) | 25-50ug | 1/2 - 1 tablet | 1/4 - 1/2 tablets | Once daily |
| Maintenance dose | 100-200ug | 2-4 tablets | 1-2 tablets | Once daily |

For secondary hypothyroidism, the cause of the disease must be determined before replacement therapy with this product and glucocorticoid supplementation should be given if necessary. Once levothyroxine therapy is established, it is recommended that its dose be adjusted according to the patient's clinical response and the laboratory tests in the event of a drug switch.

For the others, please refer to the relevant product instructions.

Appendix 9 Blood Volume Estimation

| **Time** | **Parameters to be assessed** | **Total blood volume (mL)** |
| --- | --- | --- |
| Screening Period | Serum Pregnancy test (if applicable) | 2 |
|  | Hematology, coagulation function | 4 |
|  | Blood chemistry | 5 |
|  | Hepatitis B, C, and HIV testing | 5 |
|  | HBV DNA copies (if applicable) | 3 |
|  | Thyroid function | 3 |
|  | EBV blood test (EBER positive) | 4 |
|  | ctDNA | 5 |
|  | Total | 31 |
| **Treatment Period** | | |
| CXD1 | Hematology, coagulation function | 4 |
|  | Blood chemistry | 5 |
|  | Hepatitis B, C, and HIV testing | 5 |
|  | HBV DNA copies (if applicable) | 3 |
|  | Thyroid function | 3 |
|  | EBV blood test (EBER positive) | 4 |
|  | ctDNA | 5 |
|  | Total | 29 |
| **End-of-Treatment Visit** | Hematology, coagulation function | 4 |
|  | Blood chemistry | 5 |
|  | Hepatitis B, C, and HIV testing | 5 |
|  | HBV DNA copies (if applicable) | 3 |
|  | Thyroid function | 3 |
|  | EBV blood test (EBER positive) | 4 |
|  | ctDNA | 5 |
|  | Total | 29 |
